# Red-Light-Active *N,C,N-*Pincer Bismuthinidene: Excited State Dynamics and Mechanism of Oxidative Addition into Aryl Iodides

**DOI:** 10.1021/jacs.4c16815

**Published:** 2025-02-10

**Authors:** Alexios Stamoulis, Mauro Mato, Paolo Cleto Bruzzese, Markus Leutzsch, Alejandro Cadranel, Marcos Gil-Sepulcre, Frank Neese, Josep Cornella

**Affiliations:** † Max-Planck-Institut für Kohlenforschung, Kaiser-Wilhelm-Platz 1, Mülheim an der Ruhr 45470, Germany; ‡ Max-Planck-Institut für Chemische Energiekonversion, Stiftstrasse 34–36, Mülheim an der Ruhr 45470, Germany; § Universidad de Buenos Aires, Facultad de Ciencias Exactas y Naturales, Departamento de Química Inorgánica, Analítica y Química Física, Pabellón 2, Ciudad Universitaria, C1428EHA Buenos Aires, Argentina; ∥ CONICET—Universidad de Buenos Aires, Instituto de Química Física de Materiales, Medio Ambiente y Energía (INQUIMAE), Pabellón 2, Ciudad Universitaria, C1428EHA Buenos Aires, Argentina; ⊥ Department Chemie und Pharmazie, Physikalische Chemie I, Friedrich-Alexander-Universität Erlangen-Nürnberg (FAU), Erlangen 91058, Germany; # Interdisciplinary Center for Molecular Materials, Friedrich-Alexander-Universität Erlangen-Nürnberg (FAU), Erlangen 91058, Germany

## Abstract

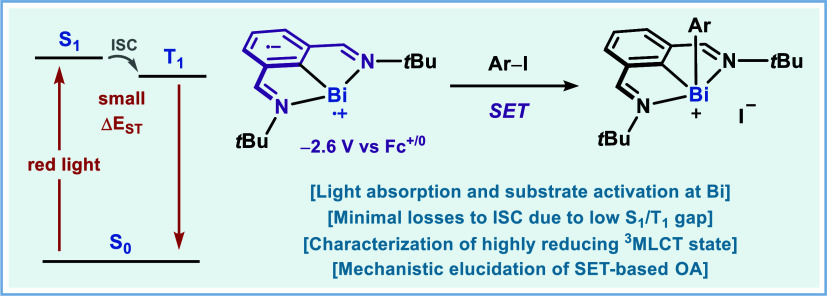

Despite the progress made in the field of synthetic organic photocatalysis over the past decade, the use of higher wavelengths, especially those in the deep-red portion of the electromagnetic spectrum, remains comparatively rare. We have previously disclosed that a well-defined *N,C,N*-pincer bismuthinidene (**1a**) can undergo formal oxidative addition into a wide range of aryl electrophiles upon absorption of low-energy red light. In this study, we map out the photophysical dynamics of **1a** and glean insights into the nature of the excited state responsible for the activation of aryl electrophiles. Transient absorption and emission techniques reveal that, upon irradiation with red light, the complex undergoes a direct S_0_ → S_1_ metal-to-ligand charge transfer (MLCT) transition, followed by rapid intersystem crossing (ISC) to a highly reducing emissive triplet state (−2.61 V vs Fc^+/0^ in MeCN). The low dissipative losses incurred during ISC (∼6% of the incident light energy) help rationalize the ability of the bismuthinidene to convert low-energy light into useful chemical energy. Spectroelectrochemical and computational data support a charge-separated excited-state structure with radical-anion character on the ligand and radical-cation character on bismuth. Kinetic studies and competition experiments afford insights into the mechanism of oxidative addition into aryl iodides; concerted and inner-sphere processes from the triplet excited state are ruled out, with the data strongly supporting a pathway that proceeds via outer-sphere dissociative electron transfer.

## Introduction

Photoredox catalysis has emerged as a privileged reaction platform for accessing novel reactivity,^1−7^ unlocking a wealth of novel retrosynthetic disconnections.^8,9^ Upon light excitation, the generated photoactive species can interact directly with a substrate or couple with catalytic cycles of transition metals,^10,11^ facilitating challenging mechanistic steps through photoinduced electron- and energy-transfer processes.^12^ Within this context, the scope of photoactive species has largely been dominated by Ru^13^ and Ir^14^ polypyridyl complexes, which feature large extinction coefficients (ε), long-lived excited states, and large redox windows of operation due to the dual reducing and oxidizing nature of their excited states. It is well-known that the *modus operandi* of these privileged photocatalyst scaffolds involves initial excitation to access a S_0_ → S_1_ transition concomitant with metal-to-ligand charge transfer (MLCT). This is generally followed by a spin-forbidden intersystem crossing (ISC) to the salient triplet state (T_1_), facilitated by spin–orbit coupling (SOC) at the heavy-atom center. For the majority of the common photocatalysts based on Ir and Ru, however, accessing the S_1_ excited states requires highly energetic blue or near-ultraviolet (UV) light, which features low penetration through reaction media and living tissue, higher scattering of shorter wavelengths (proportional to λ^–4^),^15^ and can lead to unwanted side reactivity with chromophore-bearing moieties. All these issues limit the innate sustainability and scalability of such methods.^16,17^

**Figure 1:**
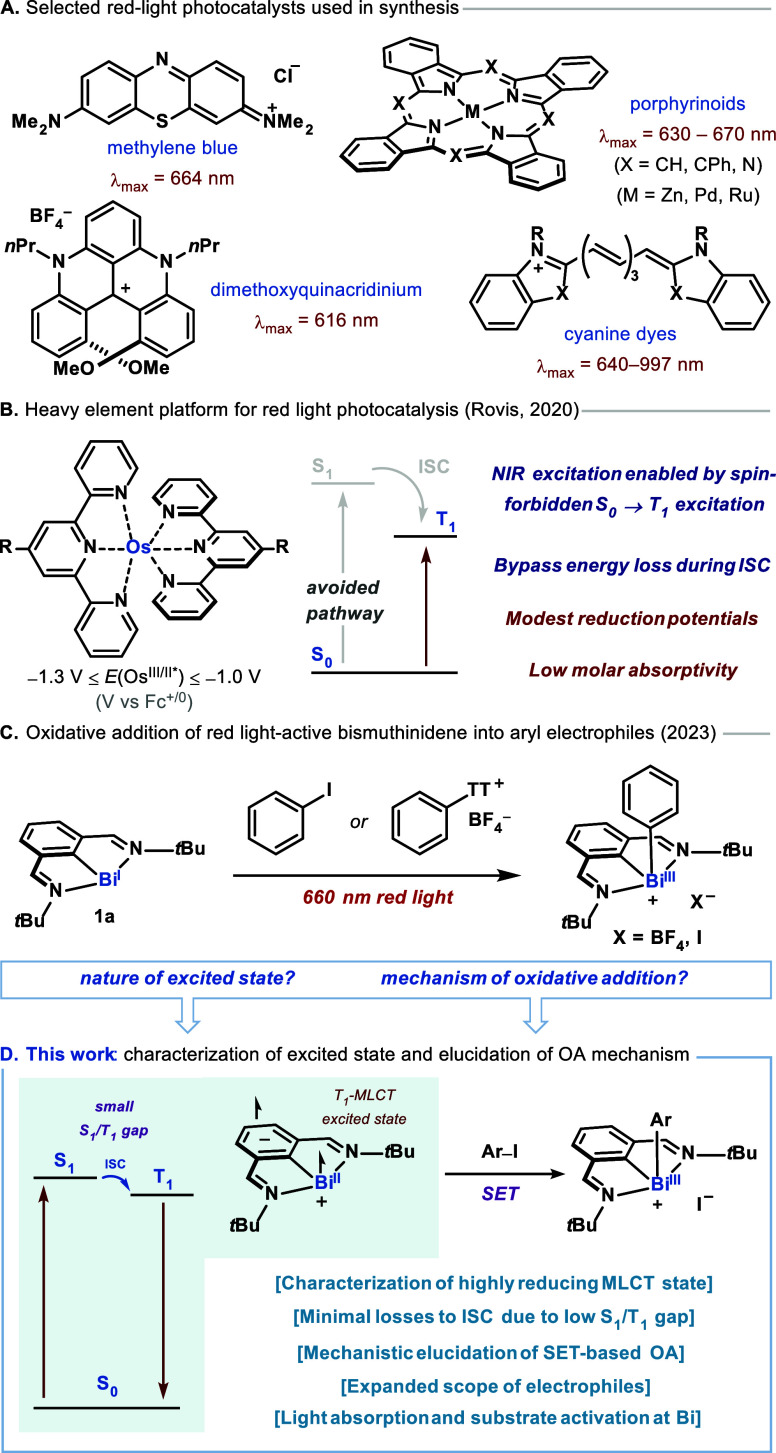
(A) Compilation of key red-light-active photocatalysts in the literature. (B) Previously reported NIR-active Os complexes featuring a direct S_0_ → T_1_ transition which bypasses dissipative losses during ISC. (C) Precedent for red-light-active bismuthinidene complex for the oxidative addition into aryl electrophiles. (D) Photophysical characterization of a red-light photoactive bismuthinidene **1a** and elucidation of the mechanism of oxidative addition into aryl iodides.

To bypass the aforementioned limitations, the last four years have witnessed a flurry of research activity aimed toward translating the arsenal of photoredox catalysis to lower-energy wavelengths, specifically in the deep red (DR) and near-infrared (NIR) regions of the electromagnetic spectrum.^18−20^ Breaking ground in this area could have applications across a range of fields, such as (batch-scalable) photocatalysis, photon upconversion, and photodynamic therapy, owing to the high penetration of red light through living tissue.^21^ Organic dyes,^22^ dimethoxyquinacridinium species,^23−26^ (aza)porphyrinoids,^27^ and cyanines^28^ are some examples of red-light-active photocatalysts that have recently emerged and found applications in synthesis (Figure 1A). Notably, a seminal report by Rovis and co-workers demonstrated that Os-based complexes ([Os(tpy)_2_]^2+^) are active photocatalysts in the 660–800 nm range (Figure 1B),^29^ due to their ability to undergo direct spin-forbidden S_0_ → T_1_ excitation facilitated by the large SOC at Os.^30,31^ The authors remark that this photophysical modality circumvents energy losses associated with ISC, which usually reach around ∼25% of the absorbed energy for commonly employed Ru and Ir bipyridyl complexes. Subsequently, several reports have shown that analogous Os polypyridyl complexes are competent red-light photocatalysts in metallaphotoredox-catalyzed C–N couplings,^32^ Cu-catalyzed difunctionalization of alkenes,^33^ light-controlled Ru-catalyzed olefin metathesis,^34^ and protein proximity labeling in tumor tissue cells.^35^ However, despite an energetically efficient excitation, the Os complexes present low molar absorptivity in the NIR (415 M^–1^ cm^–1^ at 740 nm),^29,36^ potentially requiring higher light intensities to promote desired reactivity. Furthermore, the most reducing Os(L)_2_ and Os(L)_3_ complexes capable of absorbing red light feature modest excited-state redox potentials (*E*°(Os^III/II*^)) of ca. −1.3 V vs Fc^+/0^,^36^ limiting their ability to reductively activate substrates or organometallic species with more negative *E*_red_ values.

The rich redox chemistry of Bi is brought to bear in recent reports that showcase its ability to engage in both 1e^–^ and 2e^–^ redox manifolds, featuring both analogous and complementary reactivity to that exhibited by transition-metal species.^37−43^ Recently, our group reported that (*N,C,N*)-bismuthinidenes^44,45^ undergo oxidative addition into aryl iodides and aryl thianthrenium (ArTT^+^) species under red-light irradiation, thus mimicking one of the fundamental organometallic steps in metal-catalyzed reactions (Figure 1C).^46^ Notably, the photoactive bismuthinidene harnesses low-energy red light (660 nm, 43.3 kcal mol^–1^) to enable the activation of aryl iodides with exquisite selectivity over other carbon–(pseudo)halogen bonds. Unlike most photoredox catalysts employed in the literature, the Bi species is not only the photoactive species but is also responsible for orchestrating the activation and downstream coupling of aryl iodides through elementary steps at the Bi center, thus merging aspects of photoredox and transition-metal catalysis in a single main-group species.^47^

Prompted by the unique reactivity of **1a** under red-light irradiation and buoyed by the unique photophysical behavior of other heavy atom complexes,^29^ we sought to characterize the nature of the excited-state species responsible for the observed reactivity and delineate the mechanism of the downstream oxidative addition into aryl iodides. In this study we report that the red-light absorption of bismuthinidene **1a** is attributed to an intense S_0_ → S_1_ MLCT transition (ε = 3620 M^–1^ cm^–1^ @ 630 nm), which rapidly undergoes ISC to a highly reducing triplet state (−2.61 V vs Fc^+/0^ in MeCN) (Figure 1D). However, unlike most transition-metal chromophores, the bismuthinidene features a remarkably small S_1_–T_1_ gap, leading to minimal dissipative losses during ISC. The subsequent oxidative addition into aryl iodides is interrogated using kinetic studies as well as intra- and intermolecular competition experiments. When considered holistically, the data suggest that the key ^3^MLCT state engages with aryl iodides via an outer-sphere single-electron-transfer (SET)-based mechanism, thus discarding concerted S_N_Ar-type, inner-sphere SET, or halogen-atom transfer (XAT) pathways.

## Results and Discussion

### Characterization of the Excited State

**Figure 2:**
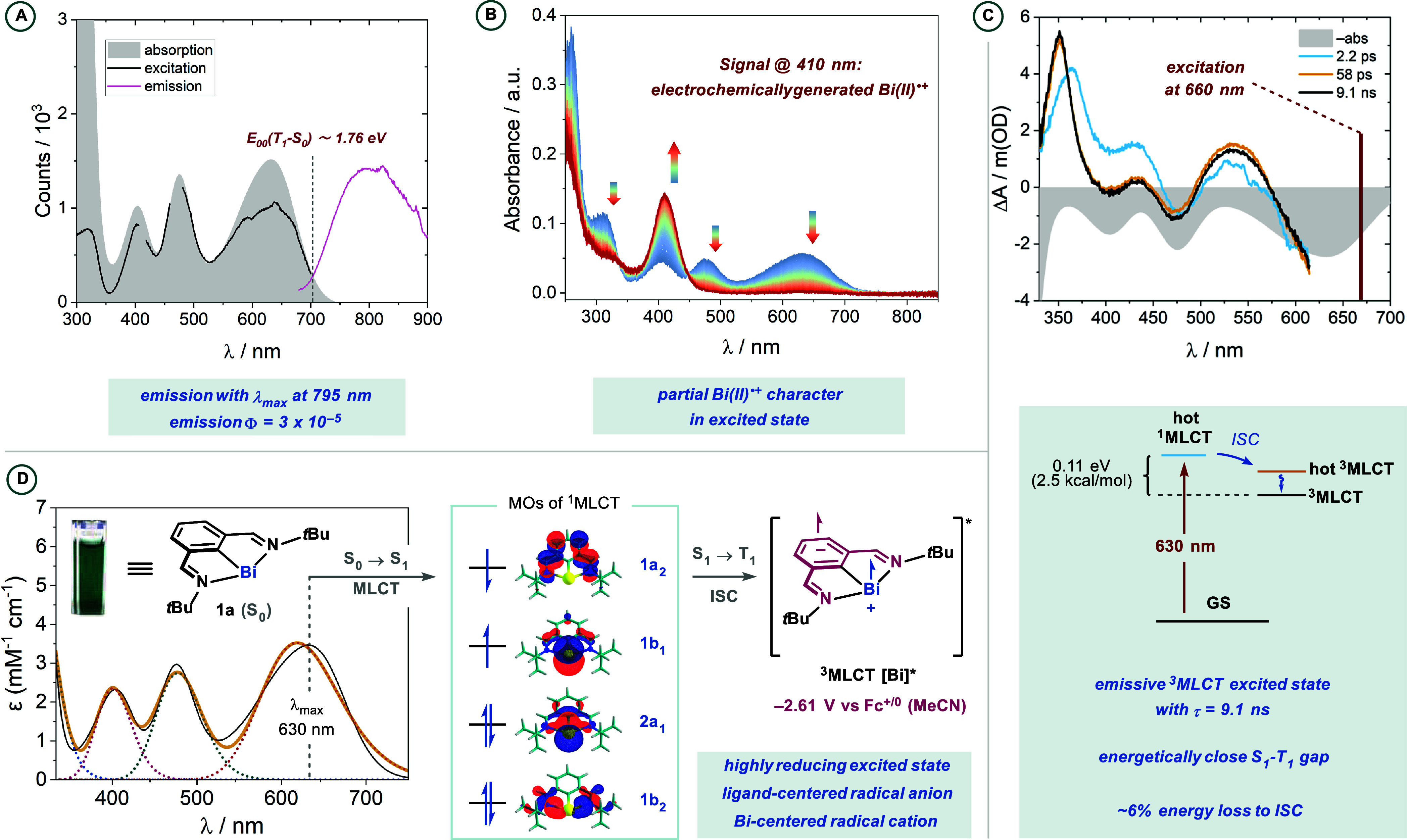
(A) Steady state UV–vis absorption (gray insert), excitation (black trace, emission detected at 795 nm), and emission (purple, excitation at 475 nm) spectra for **1a**. The dashed line shows the intersection of absorption and emission spectra, used to estimate *E*_00_(S_0_/T_1_). (B) Spectroelectrochemical studies on the bismuthinidene, tracking the evolution of the absorption spectrum during a bulk oxidation of the Bi(I) to a Bi(II) radical cation over the course of 5 min (0.11 mM solution of **1a** in acetonitrile). (C) Top: Femtosecond transient absorption spectroscopy (fsTAS) with excitation at 660 nm, revealing evolution of spectra over time. mOD = milli-optical density. Bottom: Simplified Jablonski diagram of **1a**, showing minimal losses to ISC after initial S_0_ → S_1_ transition. GS = ground state. (D) Left: UV−vis spectrum of **1a** (black trace) and spectral fitting (yellow trace) with deconvolution into individual bands represented with dashed lines. Middle: Computationally determined molecular orbital description of the ^1^MLCT state of **1a** after net HOMO–LUMO excitation, showing radical cation character on Bi and radical anion character on the *N,C,N* pincer ligand backbone. Right: Schematic representation of the ^3^MLCT state responsible for the observed reactivity.

We began our study by interrogating the photophysical behavior of **1a** via steady-state absorption and emission spectroscopies. **1a** showcases four absorption bands at 314, 405, 476, and 631 nm, which explains the intense dark green color observed in MeCN solutions. The 631 nm band is thought to arise from a Bi(I) → (*N,C,N*) MLCT transition, an assignment that is supported by its pronounced solvatochromic behavior (Figure S29). On the other hand, the remaining bands show little to no solvatochromism, suggesting they arise from predominantly metal-centered transitions. **1a** is weakly emissive in MeCN at 25 °C, with a maximum at 795 nm (12,580 cm^–1^, 1.6 eV) and a shift of only ∼3250 cm^–1^ or 0.40 eV from the lowest absorption maximum at 630 nm (15,830 cm^–1^, 1.95 eV) (Figure 2A). The λ_max_ and emission bandshapes were independent of the excitation wavelength (see Figure S31 in the Supporting Information), and the excitation spectrum tracks well with the absorption spectrum. This suggests that, irrespective of the excitation wavelength, the most important deactivation channels of high-energy excited states funnel to the same emissive excited state, in accordance with Kasha’s rule.^48^ This helps rationalize previous observations that **1a** displays the same reactivity with wavelengths ranging from 450 to 660 nm.^46,47^ Broadness of the emission band shape suggests a {Bi^II^(NCN^•–^)} MLCT origin for this excited state. An emission quantum yield of 3 × 10^–5^ was determined via comparative actinometry with [Ru(bpy)_3_]^2+^ using 475 nm excitation. This indicated that nonradiative deactivation to the ground state outcompeted emission, in line with the energy-gap law for low-energy MLCT excited states.^49^

The charge-transfer nature of the emissive excited state was examined using spectroelectrochemistry and femtosecond transient-absorption spectroscopy (fsTAS). This conceptually rests on interpreting the transient absorption spectrum of the {Bi^II^(NCN^•–^)} MLCT excited state as a linear combination of the oxidized and reduced forms of the ground state.^50,51^ In order to interrogate the potential Bi(II) character of the excited state, the spectroscopic fingerprints of Bi(I) upon oxidation were investigated. The cyclic voltammogram (CV) of **1a** in MeCN, recorded under anaerobic conditions reveals a quasi-reversible redox couple centered at *E*_1/2_ = −0.85 V vs Fc^+/0^, attributed to the Bi(I)/Bi(II) redox process (see Figures S21 and S30 in the Supporting Information). Subsequently, spectroelectrochemical experiments were performed via controlled potential electrolysis (CPE) until a stable spectrum for Bi(II) was obtained. Figure 2B illustrates the *in situ* evolution of the UV–visible (UV–vis) spectrum during an anodic applied potential, with the spectral progression monitored simultaneously. During oxidation to Bi(II), a clear increase in absorbance at 410 nm is observed, accompanied by a significant decrease in the feature at 314 nm and the disappearance of bands at 476 and 631 nm. Additionally, isosbestic points are noted at 335 and 450 nm, indicating a clean Bi(I)-to-Bi(II) transformation. The application of a reductive potential cleanly restores the original Bi(I) spectrum, confirming the chemical reversibility of the redox process and the stability of both species (see Figure S30 in the Supporting Information for more details). Next, fsTAS in MeCN at 25 °C was utilized to study the excited-state dynamics (Figure 2C). Excitation of **1a** at 600 nm afforded differential spectra with minima at 405, 475, and 635 nm corresponding to ground-state bleaching (see also Figure S32 in the Supporting Information). Additionally, maxima at 360, 430, and 530 nm were observed. During the observation time window, differential spectra evolved slightly, with a minor hypsochromic shift of the λ_max_ at 360 to 350 nm. This indicated that no major changes in the electronic configuration take place during excited-state decay. We posit that the spectral feature at 430 nm observed using fsTAS corresponds to the feature at 410 nm observed during anodic spectroelectrochemistry experiments. The minimal energy difference stems from the presence of the ligand radical anion in the MLCT state, which is absent in the Bi(II) species generated via spectroelectrochemistry. Therefore, this band is diagnostic of the formation of a species with Bi(II) character in the excited state, confirming its {Bi^II^(NCN^•–^)} MLCT nature. Global fitting of the data afforded three exponential decays, with lifetimes of 2.2 ps, 58 ps and 9.1 ns. An all-sequential target model was considered to fit the data, and the corresponding species-associated differential spectra are shown in Figure 2C. Upon excitation, a vibrationally hot ^1^MLCT state is populated (Figure 2C, blue line), which undergoes intersystem crossing to a vibrationally hot ^3^MLCT with τ = 2.2 ps (Figure 2C orange line). This state cools down in 58 ps populating the emissive ^3^MLCT, which showed a lifetime of 9.1 ns (Figure 2C, black line). The lifetime of the key emissive excited state is consistent with the ability of **1a** to engage in bimolecular reactivity. In line with steady-state spectroscopy measurements, excitation at 400, 475, or 660 nm afforded very similar results (Figures S32–S35 in the Supporting Information). This revealed that all the processes originating from Franck–Condon excited states to hot ^1^MLCT are faster than our time resolution (ca. 150 fs). Furthermore, the concentration of **1a** played no role in the range of 0.1–0.4 mM, indicating the absence of self-quenching processes in this concentration range.

Experimentally, a low-energy limit of E_00_ for ^3^MLCT can be estimated as 700 nm (14,276 cm^–1^, 1.76 eV) from the intersection of the absorption and emission spectra recorded in MeCN. Therefore, excitation with 660 nm light (15152 cm^–1^, 1.88 eV) implies dissipation of only 120 meV, equivalent to only 6% of the incident light energy (Figure 2C). There is growing interest in antidissipative strategies to improve solar-energy conversion or any photocatalytic efficiencies.^52^ Intersystem crossing has been identified as a major source of dissipation with losses of 20–30% of the incident light energy, thus limiting the efficiency of light harvesting strategies.^53,54^ In this context, the (*N,C,N*)-Bi(I) complexes can provide interesting opportunities due to the small S–T energy gaps and intense ground-state absorptions of S_0_ → S_1_ transitions (molar absorptivity of around 3620 M^–1^ cm^–1^ at 630 nm) with minimal energy losses associated with ISC, affording, for example, synthetically salient excited-state redox potentials. In fact, Rehm–Weller methods were employed to estimate the redox potential of the excited state species.^55,56^ Combining the low-energy limit of E_00_ for ^3^MLCT of 1.76 eV and the ground state redox potential of the Bi^II/I^ redox couple (*E*_1/2_ = −0.85 V vs Fc^+/0^ in MeCN) an excited state redox potential of −2.61 V vs Fc^+/0^ in MeCN can be obtained.^57^

### Electronic Structure of **1a**

Quantum chemical calculations performed with the ORCA program package^58,59^ provided insights into the electronic structure of **1a**. Scalar relativistic complete active space self-consistent field (CASSCF) with *n*-electron valence second-order perturbation theory (NEVPT2) calculations were conducted on the density functional theory (DFT)-optimized geometry of **1a** (see Section 11 of the Supporting Information for computational details) with inclusion of SOC via quasi-degenerate perturbation theory (QDPT). The calculated ground state is dominated to 95% by a totally symmetric closed shell singlet state ^1^A_1_. The highest occupied molecular orbital (HOMO) is dominated by the contributions from the Bi 6p_*z*_ orbital (orbital 1b_1_ in Figure 2D). According to our calculations, the first electronically excited state arises from an excitation of the HOMO to the lowest-unoccupied molecular orbital (LUMO) (1a_2_ in Figure 2D). This excitation gives rise to a pair of singlet and triplet states of B_2_ symmetry (^1,3^B_2_) with negligible admixture induced by SOC. This state is best characterized as a charge transfer state since the HOMO is mostly localized at the Bi center while the LUMO is dominated by a π* orbital of the ligand. The energy of the singlet ^1^B_2_ excited state is calculated to be 15,022 cm^–1^ (1.86 eV, 666 nm) in excellent agreement with the spectral feature with λ_max_ = 630 nm in the UV–vis spectrum (Figure 2D). SOC splits the corresponding ^3^B_2_ state into three pure triplet states almost degenerate (splitting of the *M*_s_ = 0 magnetic sublevel from *M*_s_ = ±1 by only 0.41 cm^–1^). This is likely an underestimate because the direct electron spin–spin coupling will also contribute to the excited state zero-field splitting (ZFS). However, given the charge-transfer nature of the excitation, it is likely that these contributions are also smaller than 1 cm^–1^ and would not change any of our conclusions. Notably, the calculated ^1^B_2_–^3^B_2_ energy gap is only 0.10 eV, again in close agreement with the experimental value. This value remains unperturbed by the SOC-induced splitting of the magnetic sublevels. The small S–T splitting is therefore amenable to the efficient charge-transfer character of MLCT(B_2_) state, which maximizes the distance between the charge centers in 1b_1_ and 1a_2_ orbitals reducing the exchange interaction of the unpaired electrons.^60^ Calculations show that excited states with bismuth-ligand antibonding character occur in the Vis regime at energy values too high (starting from 17703 cm^–1^, 2.20 eV, see also Table S4) to interfere with the ^3^B_2_ state which is therefore free to persist long enough to exploit the chemical potential stored in this charge-separated state.

### Bimolecular Quenching Studies

**Figure 3:**
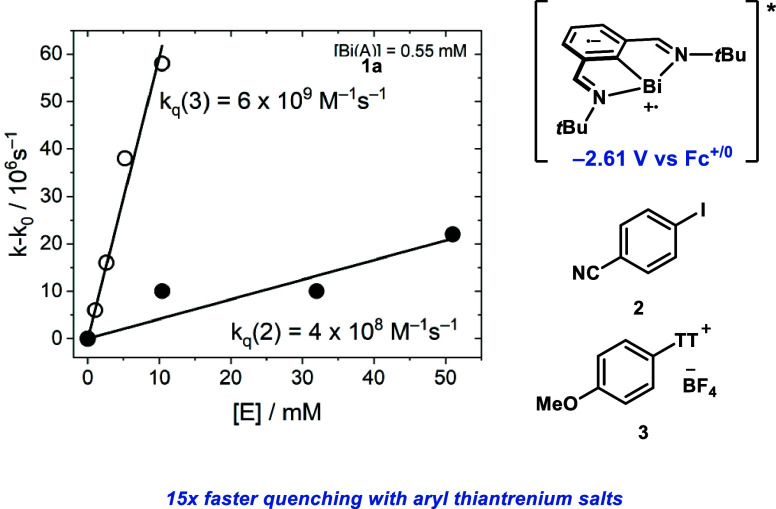
Stern–Volmer quenching studies of the excited state bismuthinidene.

Stern–Volmer quenching studies were conducted using fsTAS to study the rate of bimolecular quenching using 0.55 mM solutions of **1a** in MeCN and increasing concentrations of either 4-iodobenzonitrile **2** or arylthianthrenium **3**. In both cases, only the ^3^MLCT lifetime was significantly affected by the presence of the electrophiles. Evaluation of the rate constants derived from global analysis afforded bimolecular quenching constants of 4 × 10^8^ and 6 × 10^9^ M^–1^ cm^–1^ for **2** and **3**, respectively (Figure 3). Given that **3** (*E*_p/2_ ≈ −1.6 V vs Fc^+/0^)^61^ is a better electron acceptor than **2** (*E*_p/2_ = −2.12 V vs Fc^+/0^), and considering the calculated excited-state oxidation potential of −2.61 V vs Fc^+/0^ for **1a**, we wondered whether the order of magnitude differences in quenching constants originates merely from the driving forces for electron transfer, which probably falls in the Marcus normal region for both substrates (ca. 0.5 and 1 V overpotential for substrates **2** and **3**, respectively). Unfortunately, no differential signals arising from the products of the bimolecular reactions were detected, indicating that products react faster than their production rate. Even though it is reasonable to posit an electron-transfer/mesolytic cleavage pathway for the activation of aryl thianthrenium species, aryl iodides can be activated by several different pathways. Spurred by these large differences in quenching constants and in the overall rate of oxidative addition,^46^ a study was conducted to gain further insight into the mechanism of oxidative addition into aryl iodides.

### Concerted vs Stepwise Aryl Iodide Oxidative Addition

**Figure 4:**
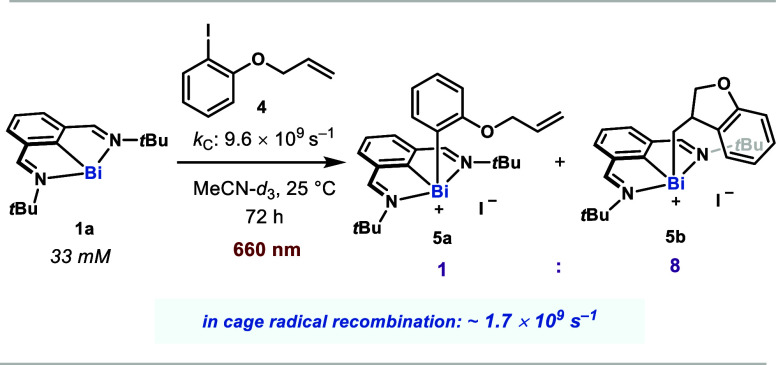
Radical clock experiment to probe concerted vs stepwise oxidative addition pathways.

Efforts toward probing the mechanism of oxidative addition started by differentiating between concerted (S_N_Ar-type) and stepwise (SET, XAT) pathways. Since the latter pathways feature aryl radicals as obligate intermediates, we conducted the oxidative addition of **1a** into 1-(allyloxy)-2-iodobenzene **4** as an aryl radical trap (Figure 4). Under red light irradiation, the reaction afforded a ca. 1:8 mixture of the noncyclized (**5a**) to cyclized (**5b**) oxidative addition products. It is worth noting that the known stability of *N,C,N*-biaryl species under red light irradiation precludes conversion of **5a** to **5b**. This result strongly supports the existence of an aryl radical intermediate during the mechanism of formal oxidative addition. Since the nascent aryl radical derived from this substrate is known to undergo a 5-exo-trig cyclization with a rate constant of 9.6 × 10^9^ s^–1^,^62^ the ratio of products allowed us to clock the effective first-order rate constant of radical recombination between the solvent-caged aryl radical and the putative Bi(II) radical at ca. 1.7 × 10^9^ s^–1^, which is likely approaching the diffusion limit.^63^ Additionally, UV–vis and ^1^H NMR experiments do not suggest the formation of a photoactive electron donor–acceptor (EDA) complex between **1a** and **2** (see Sections 8.1 and 10.3 in the Supporting Information), thereby strongly suggesting that the observed reactivity arises from an interaction of the excited ^3^MLCT state of **1a** with the aryl iodide.

**Figure 5:**
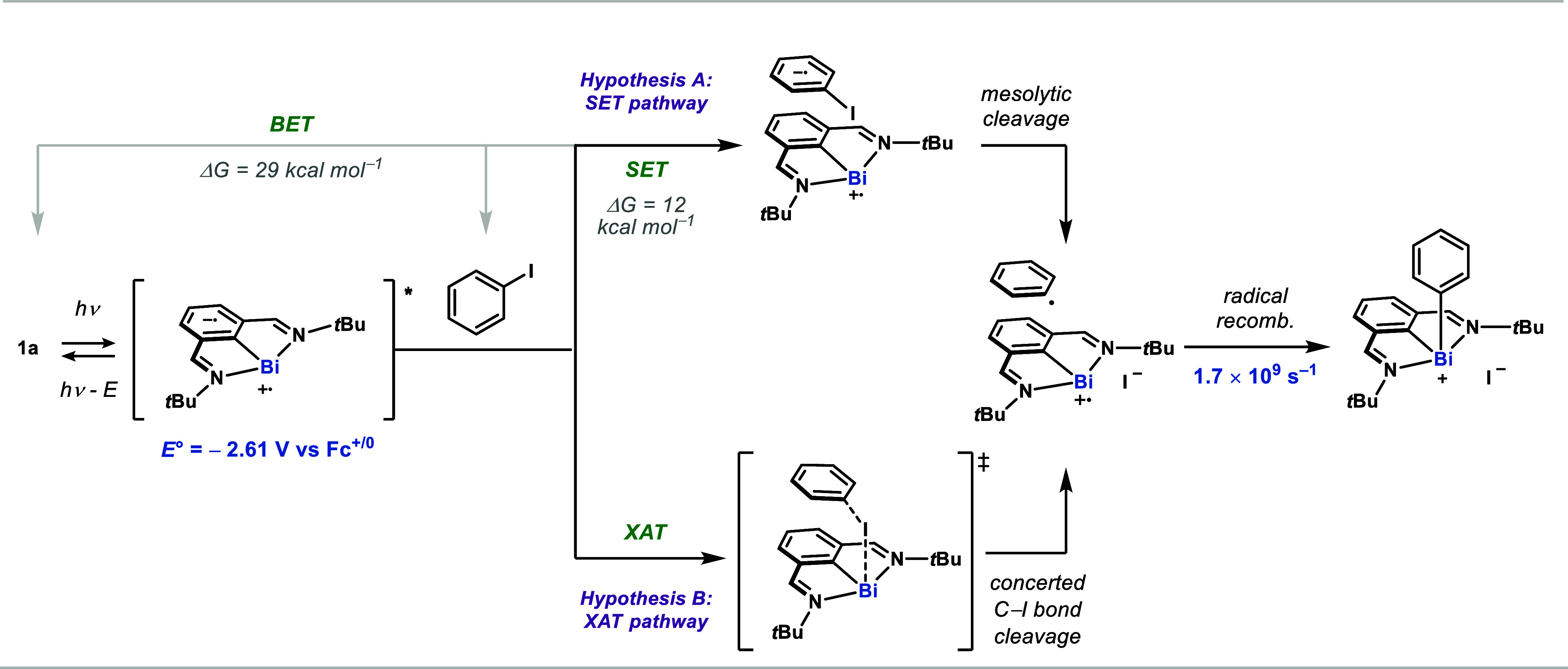
SET vs XAT. *Hypothesis A* invokes an inner or outer sphere electron transfer process from the excited bismuthinidene to the aryl iodide, leading to an aryl radical after C–I mesolytic cleavage. *Hypothesis B* features an irreversible and concerted halogen atom transfer between the excited-state bismuthinidene and the aryl iodide to afford the solvent-caged radical pair. Gray lines denote back electron transfer processes (BET).

Taking the above data altogether, two main stepwise mechanistic scenarios were considered. The first scenario invokes single-electron transfer from the bismuthinidene excited state to the aryl iodide (Figure 5, *hypothesis A*). Given the redox potentials of the excited state (*E*°(Bi^II/*^) = −2.61 V vs Fc^+/0^) and aryl iodide **2** (*E*_p/2_ ≈ – 2.10 V vs Fc^+/0^), this amounts to an exergonic electron-transfer event (12 kcal mol^–1^), which most likely falls in the Marcus normal region. The downstream productive mesolytic cleavage competes with back-electron transfer (BET), which has a driving force of ca. 29 kcal mol^–1^ (*E*_1/2_(Bi^II/I^) = −0.85 V vs Fc^+/0^) and returns the ground state bismuthinidene and neutral aryl iodide. It is worth noting that the dissociative electron transfer can also occur in a concerted fashion.^64−70^ The second main mechanistic pathway considered involves a concerted, irreversible XAT event between the excited-state bismuthinidene and the aryl iodide (Figure 5, *hypothesis B*). Unlike the first mechanistic scenario, the XAT pathway would not be subject to competing back-electron transfer events.

### Reaction Orders and Hammett Studies

**Figure 6:**
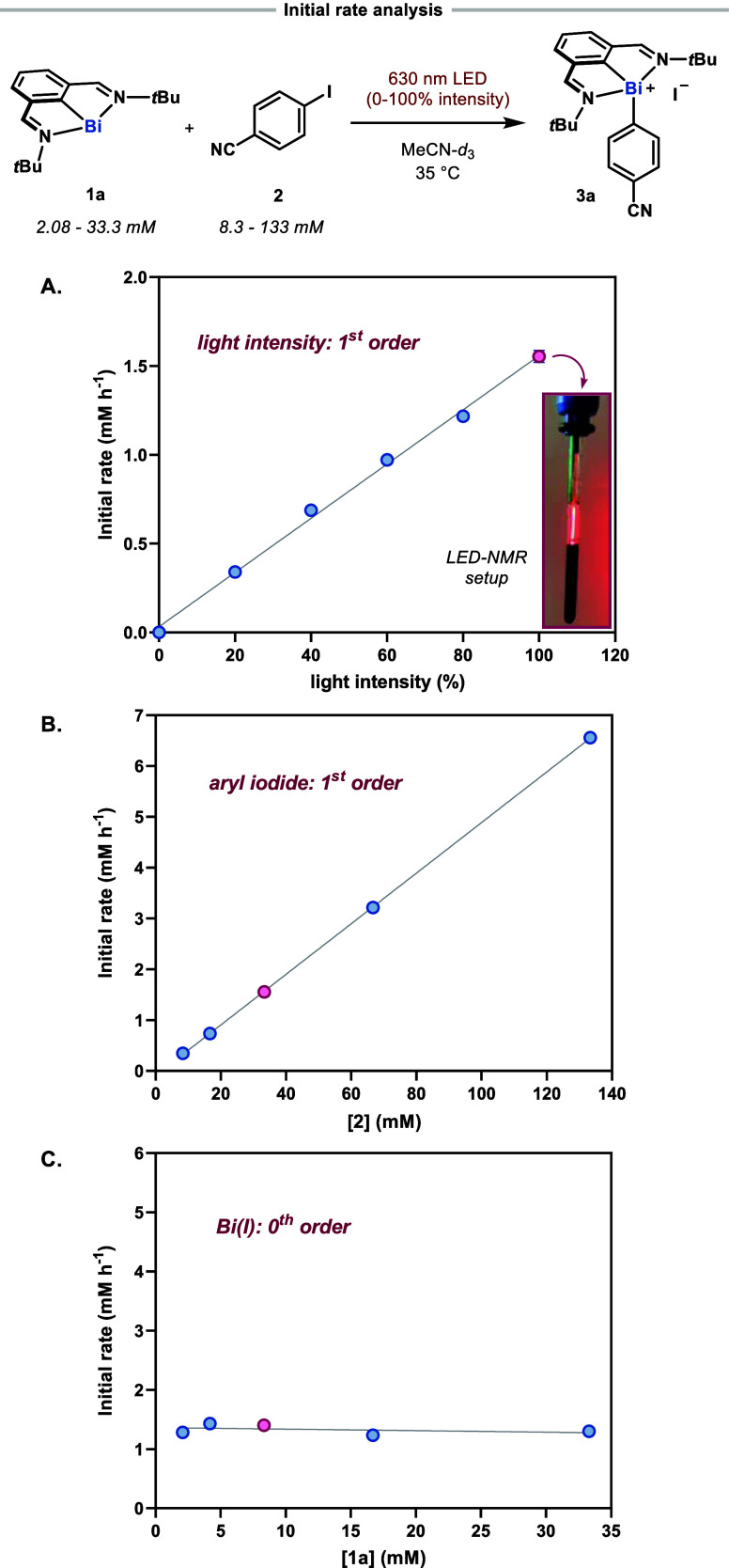
Kinetic dependence of Bi(I), aryl iodide, and light intensity obtained via initial rate analysis. Picture refers to the LED-NMR setup used in these studies. Data points in purple denote “standard” conditions employed in kinetic studies: 8.3 mM **1a**, 33.3 mM **2**, 100% light intensity, MeCN-*d*_3_, 35 °C.

Kinetic studies were conducted to gain more insight into the mechanism of oxidative addition of Bi(I) into the C–I bond of aryl iodides. Reactions were carried out in an NMR spectrometer for *in situ* monitoring of the reaction progress, using the fiber optic-coupled LED setup as previously described by Gschwind and co-workers (see Section 5.1 in the Supporting Information for more experimental details).^71−74^ Initial rates were obtained from the plotted concentration profiles at ≤10% conversion. To accurately mimic the experimental conditions during Kessil light irradiation, a constant temperature of 35 °C was maintained during the *in situ* LED-NMR time courses using the active temperature control capabilities of the NMR instrument (see Section 5.1 in the Supporting Information). The rate of oxidative addition of **1a** into 4-iodobenzonitrile exhibits a first-order dependence on the light intensity and the aryl iodide concentration (Figure 6A, 6B, and Sections 5.3 and 5.4 in the Supporting Information). Somewhat surprisingly, the initial rates were independent of the Bi(I) concentration within the range of 2–33 mM (Figure 6C and Section 5.5 in the Supporting Information). The latter can be rationalized by invoking a light-limited formation of the excited-state bismuthinidene, likely due to the high molar absorptivity for the wavelength of light used (ε = 3620 M^–1^ cm^–1^ @ 630 nm). Overall, the kinetic dependence on the various reaction components suggests a rate-limiting interaction of the excited-state bismuthinidene and the aryl iodide.

**Figure 7:**
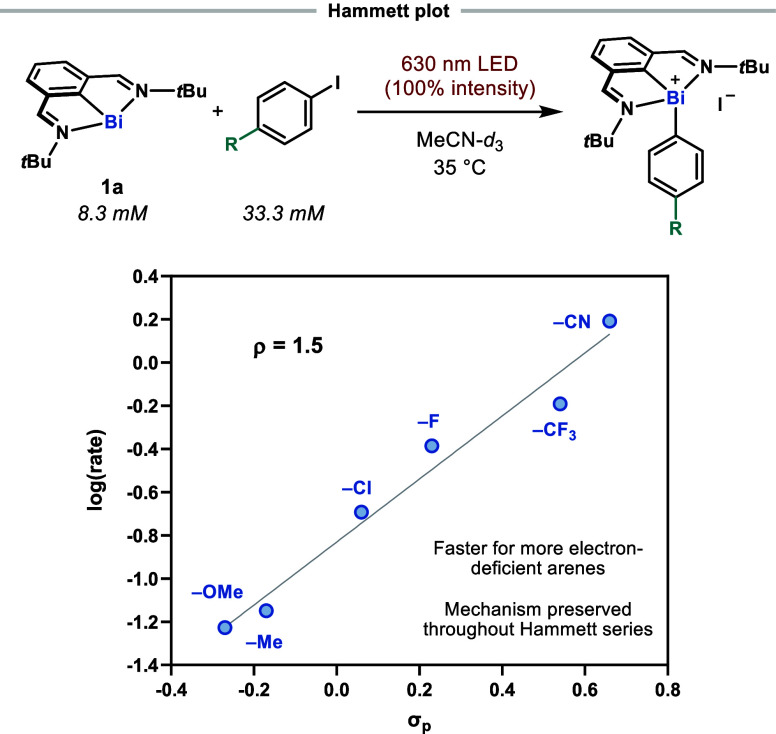
Hammett plot obtained via initial rate analysis of red light-promoted reactions of **1a** with various *para*-substituted aryl iodides. Log of the initial rates are plotted against the σ_p_ values of the substituents. Conditions: 8.3 mM **1a**, 33.3 mM aryl iodide, 100% light intensity, MeCN-*d*_3_, 35 °C.

A Hammett plot was constructed from initial rates of oxidative addition with a series of *para*-substituted aryl iodides (Figure 7). Plotting the initial-rate data vs standard Hammett σ_p_ values gave the best fit of the data, revealing a positive correlation with ρ = 1.5. This is consistent with a net buildup of negative charge on the aryl iodide in the rate-limiting step. Furthermore, the linearity of the plot also suggests no change in mechanism across the series of aryl iodides tested. Although smaller ρ values have typically been used to support the existence of XAT- over SET-based mechanisms for oxidative additions at transition-metal centers,^75^ we hypothesized that an attenuated ρ value could also arise from a concerted dissociative electron transfer mechanism or a stepwise mechanism featuring rate-limiting contributions from both electron transfer and mesolytic cleavage steps (whose rates will increase and decrease, respectively, for more electron-deficient arenes). With this in mind, we sought to probe this mechanistic dichotomy further by conducting a series of intra- and intermolecular competition studies.

### Intra- and Intermolecular Competition Studies

**Figure 8:**
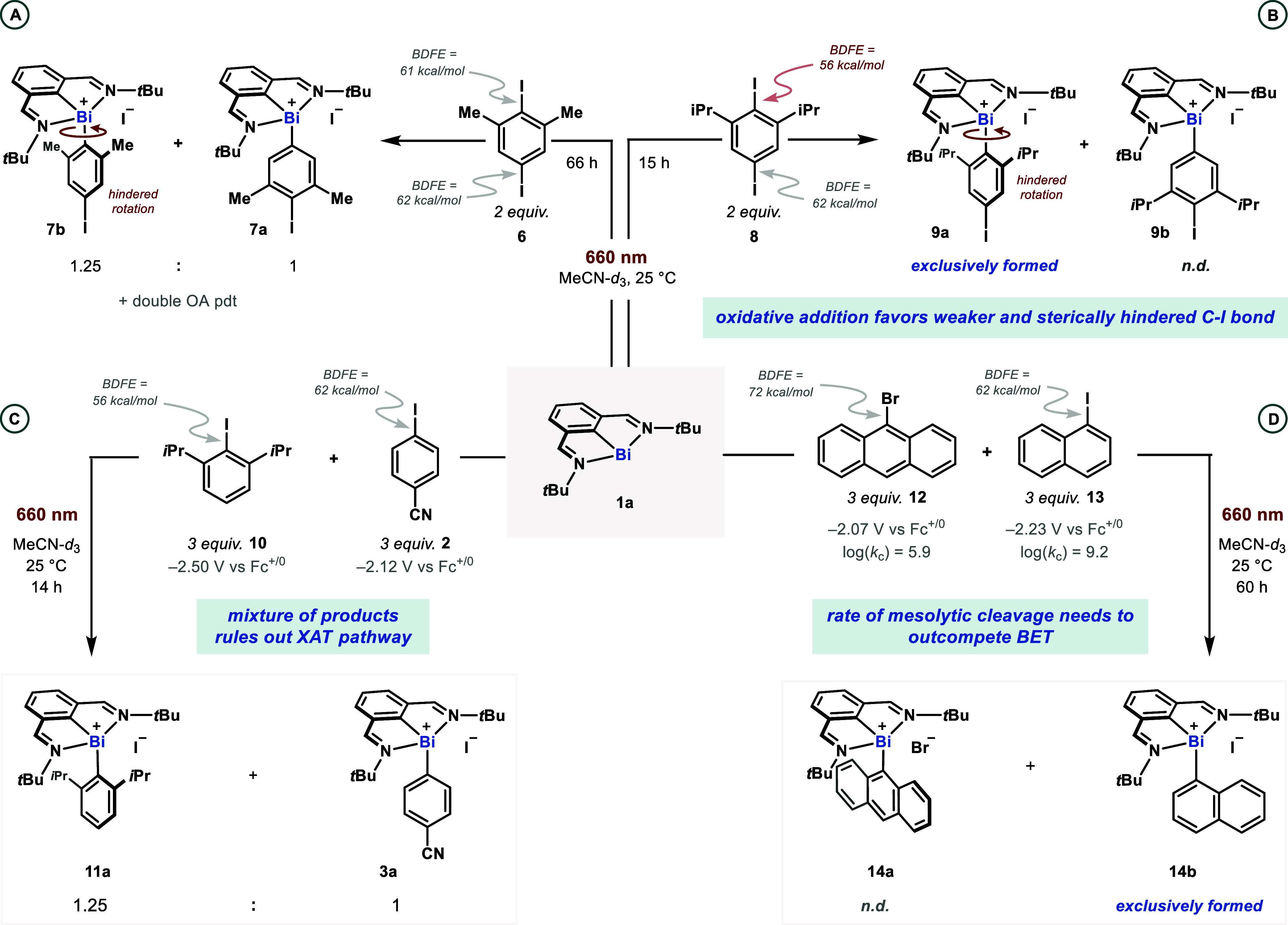
Intra- and intermolecular competition experiments to probe the preference for SET vs XAT reactivity. All competition experiments were performed with bismuthinidene **1a** as the limiting reagent. (A) Intramolecular competition experiment with 2,5-diiodo-1,3-dimethylbenzene **6** under standard reaction conditions. (B) Intramolecular competition experiment with 2,5-diiodo-1,3-diisopropylbenzene **8**. (C) Intermolecular competition experiment between 2-iodo-1,3-diisopropylbenzene **10** and 4-iodobenzonitrile **2**. (D) Intermolecular competition between 9-bromoanthracene **12** and 1-iodonaphthalene **13**.

To distinguish between SET and XAT pathways, a series of intra- and intermolecular competition reactions were designed. In a previous study, Galli and co-workers reported that *in situ* generated tributyltin radical (Bu_3_Sn^•^) exclusively abstracts the iodine atom from the more sterically hindered C–I bond in 2,5-diiodo-1,3-dimethylbenzene (**6**).^76^ This counterintuitive selectivity was attributed to the pinching effect of the *ortho*-methyl groups, which the authors purport leads to a weakening of the neighboring C–I bond. By analogy to an “effective” bond dissociation energy (BDE) of the C–I bond in iododurene, obtained via extrapolation to a linear free energy relationship (LFER) plot of other iodoarenes,^77^ the BDE of the crowded C–I bond in **6** is assumed by the authors to be 49 kcal mol^–1^. This is significantly weaker than the BDE of the unhindered C–I bond, which is assumed to have the same C–I bond BDE as found in iodobenzene (69 kcal mol^–1^).^76^ In our case, the intramolecular competition between **1a** and **6** leads to a 1:1.25 mixture of products (**7a** and **7b**), with a minor side-product arising from double oxidative addition into the diiodoarene (**7c**, see Section 6.2 in the Supporting Information) (Figure 8A). This result reveals an approximately equal preference for oxidative addition into either C–I bond. According to these previously reported assumptions on BDE differences, this lack of a clear preference for oxidative addition into the more crowded C–I bond already serves as evidence against an XAT pathway. However, in contrast with these previously purported energies of the two C–I bonds of **6**, optimization of this substrate *in silico* revealed that both C–I bonds feature almost identical bond dissociation free energies (BDFEs) of ∼62 kcal mol^–1^. Therefore, although simple ground-state energies of the bonds are not enough to rationalize the previously reported selectivity with tributyltin radical, it is still possible that the *o,o*′-dimethyl substituents kinetically favor elongation of the C–I bond in the transition state for XAT, leading to a transition-state-stabilizing effect that largely outweighs any unfavorable steric clash between the methyl groups and the organotin species. To further solidify this notion, we synthesized 2,5-diiodo-1,3-diisopropylbenzene (**8**), to exacerbate the difference in BDFEs and steric hindrance between the competing C–I bonds (Figure 8B). Optimization of this substrate revealed a significantly lower BDFE for the more sterically crowded C–I bond (56 kcal mol^–1^ vs 62 kcal mol^–1^), which also featured significant out-of-plane bending to alleviate the steric strain with the neighboring isopropyl substituents (see Section 11.3 in the Supporting Information). Remarkably, employing this substrate under standard conditions *exclusively* afforded **9a**, arising from the oxidative addition into the more sterically hindered C–I bond. Indeed, the ^1^H NMR spectrum of the only product in the latter experiment featured sharp, distinct signals for magnetically nonequivalent isopropyl groups and arene C–H bonds. In addition, the expected signals for homotopic protons on the pincer backbone were observed, thus indicating highly hindered rotation about the newly forged Bi–C bond. Yet, this result could still arise from either an XAT mechanism that favors a halogen abstraction of the weaker and more sterically hindered C–I bond, or an SET mechanism with a product-determining mesolytic cleavage step that favors fragmentation of the weaker, out-of-plane C–I bond.

To conclusively differentiate between these two possibilities, an intermolecular competition experiment between 2-iodo-1,3-diisopropylbenzene (**10**) and 4-iodobenzonitrile (**2**) was conducted (Figure 8C). This led to the formation of a 1.25:1 mixture of products **11a** and **3a**, with the reaction only slightly favoring oxidative addition into the former substrate. This result is inconsistent with an XAT pathway, where an almost exclusive oxidative addition into the weaker C–I bond would be expected, as seen in the intramolecular competition in Figure 8B. Instead, a mixture of products is suggestive of an SET-based oxidative addition mechanism featuring a competition between a substrate that is easier to reduce (− 2.12 V vs Fc^+/0^) but has a slower rate of mesolytic cleavage (**2**) and a substrate that is harder to reduce (−2.50 V vs Fc^+/0^) but undergoes rapid mesolytic cleavage (**10**). This also helps rationalize the results of the intramolecular competition experiment with **8**, where the faster rate of a product-determining mesolytic cleavage step from the more sterically crowded C–I bond leads to the exclusive selectivity for product **9a**. Indeed, the increased rate of mesolytic cleavage from the radical anions of *ortho*-substituted aryl halides has been documented in the literature,^68,78^ and was recently exploited very elegantly in a report by Pierson and Hartwig to elucidate the mechanism of oxidative addition of low-valent Ni species into aryl halides.^79^ In their report, the authors disclosed increasing rates of oxidative addition of low-valent Ni species into 4-(*tert*-butyl)-1-iodobenzene <1-iodo-2-isopropylbenzene <2-iodo-1,3-diisopropylbenzene, which was taken as preference for an outer- over an inner-sphere mechanism. Along the same lines, the ability of **1a** to perform oxidative addition into more sterically hindered C–I bonds is also evidence against the existence of an inner-sphere SET or XAT mechanism that requires a close interaction of the iodoarene and the bulky excited-state triplet bismuthinidene. Additional circumstantial evidence in favor of the SET pathway between **1a** and aryl iodides includes the 7-fold reduction in reaction yield when the reaction is conducted in pentane vs MeCN (see Section 10.1 of the Supporting Information). This dramatic difference in rate is more easily attributed to the destabilization of the ion pair formed after an electron-transfer event, since a similar destabilization would not be expected for a neutral, concerted transition state for XAT.

To further probe the importance of the rate of mesolytic cleavage, an intermolecular competition was conducted between 9-bromoanthracene (**12**) and 1-iodonaphthalene (**13**) (Figure 8D),^79^ both of which are known to go through stepwise dissociative electron transfer pathways. While the former has a more positive reduction onset potential (− 2.07 V for **12** vs −2.23 V for **13**), the radical anion of the latter is known to mesolytically cleave 2000-fold faster.^80^ The reaction afforded exclusive formation of **14b**, arising from oxidative addition into 1-iodonaphthalene. Together with the experiment in Figure 8B, this suggests that mesolytic cleavage is *at least* product-determining in the mechanism due to the competition with unproductive back-electron transfer. However, the combined data from the Hammett plot and the competition reaction in Figure 8C suggest that the rate of mesolytic cleavage (vs back-electron transfer) and the rate of electron transfer may both contribute to the observed rate of the reaction. Indeed, this combined influence of the electron transfer (favored for more electron-deficient arenes) and the rate of mesolytic cleavage (favored for more electron-rich arenes) may also help rationalize the low ρ value observed in the Hammett plot obtained for a mechanism proceeding via a SET mechanism (Figure 7). It is worth pointing out that the Hammett series obtained points against mesolytic cleavage being the sole rate-determining step, since a correlation with a negative ρ value would be expected. The mechanistically grounded importance of the mesolytic cleavage step can rationalize the exquisite selectivity of this system for oxidative addition into aryl thianthrenium species and aryl iodides,^46^ which have significantly faster rates of radical anion fragmentation relative to other aryl (pseudo)halides.^65,81^

**Figure 9:**
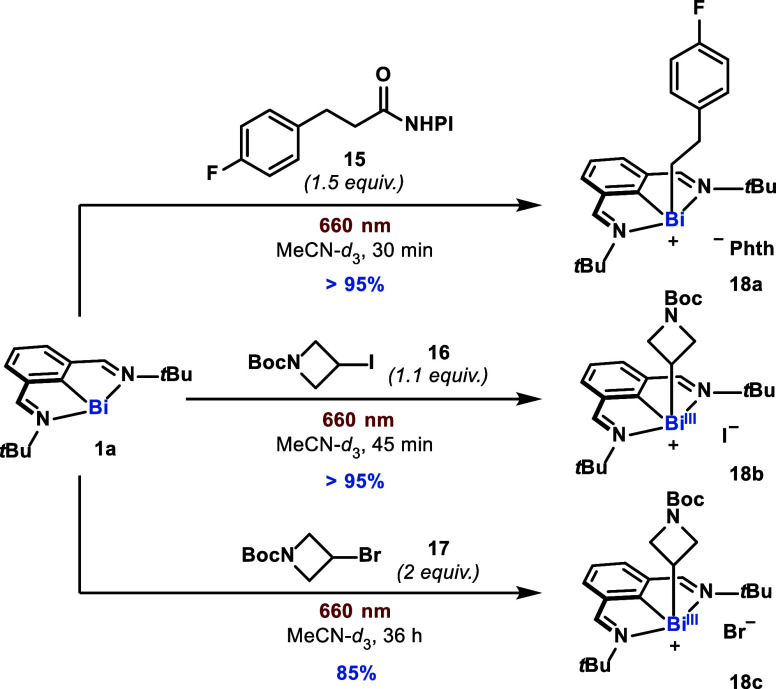
Red-light-promoted oxidative addition of bismuthinidene **1a** into redox-active esters (**15**), secondary alkyl iodides (**16**), and secondary alkyl bromides (**17**).

Based on the mechanistic picture established for the oxidative addition process into aryl iodides, we entertained the possibility of expanding the scope of electrophiles that can be reductively activated by the combination of **1a** and red light. We found that several classes of alkyl electrophiles that are unreactive in the presence of **1a** in the ground state (dark), can be cleanly activated in the presence of red light. The red-light-active **1a** could readily undergo SET-based oxidative additions into nonchlorinated redox active esters, as well as secondary alkyl iodides and bromides (Figure 9). These preliminary results lay the foundation for expanding the repertoire of substrates that can be activated, and subsequently coupled, using *N,C,N*-bismuthinidene scaffolds.

## Conclusions

In summary, we have mapped out the excited state nature and dynamics of red-light-active bismuthinidene **1a** and elucidated the mechanism by which the key ^3^MLCT excited state can effect net oxidative addition into aryl iodides. The photophysics were mapped out using fsTAS, revealing a red-light absorption attributed to a S_0_ → S_1_ transition, which is promptly followed by an ISC and vibrational cooling to populate an emissive triplet excited state with a lifetime of 9.1 ns. The ability of the Bi complex to undergo ISC with dissipative losses as low as 6% of the energy of the incoming light is noteworthy in the context of light harvesting strategies. The nature of the excited state was determined using CASSCF/NEVPT2, which calculated a p_*z*_-MLCT state with radical anion character on the ligand and radical cation character on bismuth. Spectroelectrochemical measurements support Bi(II)^•+^ character in the excited state. For the investigation of the mechanism of oxidative addition into aryl iodides, kinetic studies revealed a first-order dependence on light intensity and aryl iodide concentration, and a zeroth order dependence on the concentration of **1a**, indicating that the formation of the excited state under the reaction conditions is in a light-limited regime. Intra- and intermolecular competition studies allowed us to dismiss inner-sphere pathways for the activation of the aryl iodide (either via XAT or inner-sphere electron transfer) and suggest an outer-sphere SET-based activation of the aryl iodide as the operant mechanism. The combination of the kinetic and competition studies suggests a complex rate law, where the rate of the reaction is influenced by both the reduction potential of the aryl iodide, as well as the rate of mesolytic cleavage of the C–I bond from the corresponding radical anion. We anticipate that this mechanistic understanding of excited-state reactivity will act as a foundation for the development of next-generation red-light-active bismuth photocatalysts and aid in expanding the synthetic repertoire of bismuth redox catalysis. Furthermore, the recognition of the low dissipative losses incurred during ISC will spur efforts to explore the application of related bismuth complexes for light-harvesting energy conversion and photon upconversion processes. Such efforts are currently underway in our laboratories and will be reported in due course.

## Supporting Information Available

Additional experimental details; materials, methods, and equipment details; photographs of experimental setups; synthesis and characterization of all relevant ligands, metal complexes, and substrates; cyclic voltammetry data; spectroelectrochemistry data; spectroscopic data; computational methods, results, and Cartesian coordinates (PDF)
