# Supplementary material for: Red-Light-Active N,C,N-Pincer Bismuthinidene: Excited State Dynamics and Mechanism of Oxidative Addition into Aryl Iodides
Source: J Am Chem Soc. 2025 Feb 10;147(7):6037–48. doi: 10.1021/jacs.4c16815 (PMC11848931; doi:10.1021/jacs.4c16815)
Supplement: Supplementary file 1 — ja4c16815_si_001.pdf [file ja4c16815_si_001.pdf]

Supporting Information

Red-Light-Active *N,C,N*-Pincer Bismuthinidene: Excited State Dynamics and Mechanism of Oxidative Addition into Aryl Iodides

Alexios Stamoulis,[a] Mauro Mato,[a] Paolo Cleto Bruzzese,[b] Markus Leutzsch,[a] Alejandro Cadranel,[c,d,e,f] Marcos Gil-Sepulcre,[b] Frank Neese,[a] Josep Cornella[a*]

[a] Max-Planck-Institut für Kohlenforschung, Kaiser-Wilhelm-Platz 1, Mülheim an der Ruhr, 45470, Germany.

[b] Max-Planck-Institut für Chemische Energiekonversion, Stiftstrasse 34–36, Mülheim an der Ruhr, 45470, Germany.

[c] Universidad de Buenos Aires, Facultad de Ciencias Exactas y Naturales, Departamento de Química Inorgánica, Analítica y Química Física, Pabellón 2, Ciudad Universitaria, C1428EHA, Buenos Aires, Argentina

[d] CONICET – Universidad de Buenos Aires. Instituto de Química Física de Materiales, Medio Ambiente y Energía (INQUIMAE), Pabellón 2, Ciudad Universitaria, C1428EHA, Buenos Aires, Argentina

[e] Department Chemie und Pharmazie, Physikalische Chemie I, Friedrich-Alexander-Universität Erlangen-Nürnberg (FAU), 91058 Erlangen, Germany

[f] Interdisciplinary Center for Molecular Materials, Friedrich-Alexander-Universität Erlangen-Nürnberg (FAU), 91058 Erlangen, Germany

cornella@kofo.mpg.de

Supporting Information

1. General considerations 4

2. Light sources and photoreaction set-ups 6

3. Synthesis of bismuthinidene **1a** 7

4. Synthesis of aryl iodides 10

4.1. Synthesis of 1-(allyloxy)-2-iodo-benzene (**4**) 10

4.2. Synthesis of 2,5-diiodo-1,3-dimethylbenzene (**6**) 10

4.3. Synthesis of 2,5-diiodo-1,3-diisopropylbenzene (**8**) 12

4.4. Synthesis of 2-iodo-1,3-diisopropylbenzene (**10**) 13

5. Kinetic Experiments 15

5.1. LED-NMR Kinetics Measurements 15

5.2. Representative time course (**1a** + **2**) 16

5.3. Dependence on light intensity (0 – 100%) 19

5.4. Dependence on aryl iodide **2** (8.3 – 133.3 mM) 21

5.5. Dependence on **1a** (2.1 – 33.3 mM) 23

5.6. Hammett plot 26

6. Radical clock and competition experiments 28

6.1. Intramolecular competition experiment with 2,5-diiodo-1,3-dimethylbenzene (**6**) 28

6.2. Intramolecular competition experiment with 2,5-diiodo-1,3-dimethylbenzene (**6**) 35

6.3. Intramolecular competition experiment with 2,5-diiodo-1,3-diisopropylbenzene (**8**) 38

6.4. Intermolecular competition experiment between 2-iodo-1,3-diisopropylbenzene (**10**) and 4-iodobenzonitrile (**2**) & independent synthesis of **11a** 40

6.5. Intermolecular competition experiment between 2-iodo-1,3-diisopropylbenzene (**10**) and 4-iodobenzonitrile (**2**) 43

7. Electrochemical data 45

8. Photophysical properties 49

8.1. UV-Vis absorption and emission spectroscopy 49

8.2. Spectroelectrochemistry 52

8.3. Transient absorption spectroscopy 54

8.4. Rehm-Weller estimation of excited-state redox potentials 58

9. Oxidative additions into alkyl electrophiles 59

10. Additional experimental data 62

10.1. Oxidative addition reactions in MeCN vs pentane 62

10.2. Influence of wavelength on oxidative addition 66

10.3. Testing for a ground-state interaction via ^{1}H NMR 67

11. Computational analysis 68

11.1 Computational studies of **1a** 68

11.2 Computational details 70

11.3 Cartesian coordinates 74

12. NMR spectra 86

12. References 101

1. General considerations

Unless otherwise stated, all manipulations were performed under argon using standard Schlenk-line techniques or in an argon-filled glovebox.

Instruments

NMR data were recorded using Bruker AVIII HD 300 MHz, Bruker AVIII HD 400 MHz, Bruker AVIII 500 MHz, or Bruker AVNeo 600 MHz NMR spectrometers (at 298-300 K, unless stated otherwise). ^{1}H and ^{13}C chemical shifts are reported in ppm relative to the solvent residual peaks as an internal reference. For ^{1}H NMR the following residual proton peaks of the deuterated solvents were used: $CDCl_3$, δ_H($CHCl_3$) 7.260; CD_3CN, δ_H(CHD_2CN) 1.940. For ^{13}C NMR: $CDCl_3$, δ 77.16; CD_3CN, δ 1.32. ^{13}C spectra were acquired with broadband ^{1}H decoupling unless mentioned otherwise. Chemical shifts (δ) are given in ppm, relative to deuterated solvent residual peak, and coupling constants (*J*) provided in Hz. ^{19}F NMR shifts are reported relative to the ^{19}F resonances of $CFCl_3$. ^{19}F data at 282 MHz NMR is generally reported with ^{1}H decoupling. ^{15}N NMR shifts are reported relative to the ^{15}N resonances of $MeNO_3$. Chromatographic purifications were performed by flash column chromatography using Merck silica gel 60 (40-63 µm).

Absorption spectra were collected with a Shimadzu UV-1900i spectrometer and a Cary 6000i UV-Vis-NiR spectrophotometer. Steady-state emission spectroscopy was performed using an Edinburgh FS5 spectrofluorimeter, using solutions with absorbance of 0.06 at 630 nm. Ultrafast transient absorption experiments were conducted using an Astrella-F-1K amplified Ti:sapphire femtosecond laser system from Coherent, operating at a repetition rate 1kHz, 5.5 W power (5 mJ pulse energy), pulse duration of 80 fs, with TA pump / probe Helios detection system from Ultrafast Systems. White light was generated focusing a fraction of the fundamental 800 nm output onto a 2 mm CaF_2 mounted on a translating crystal holder. A 1.2 mJ fraction of the fundamental is used for pump beam generation by a TOPAS Prime from Light Conversion with standard NirUVis extension. A magic angle configuration between pump and probe polarization directions was used to avoid rotational dynamics. Bandpass filters with ± 5 or ± 10 nm were used to ensure low spectral width and to exclude 800 nm photons. Transient absorption measurements were conducted in a 2 mm quartz cuvette under argon atmosphere, using solutions with absorbances of 0.5-0.7 under stirring.

Spectroelectrochemical study was conducted in an optically transparent quartz spectroelectrochemical cuvette (Ocean Optics, path length ca. 1 mm) inside a N_2-filled glovebox (< 1 ppm O_2). This cell contains a Pt grid electrode (working), a Pt wire electrode (counter) and a silver wire pseudo reference electrode (*ca.* -0.1 V respect to $Fc^{+/0}$). The cuvette was filled with ca. 0.5 ml of a 0.11 mM complex solution in MeCN containing 0.1 M of tetrabutylammonium hexafluorophosphate ($TBAPF_6$) inside a N_2-filled glovebox. The UV-Vis evolution during an applied potential was measured using a standard Ocean optics sample stage for 1 cm cuvettes and a fiber optic setup calibrated for the measurements. The absorbance spectra were collected using a USB2000+UV-VIS-ES spectrometer from Ocean Optics (1.5 nm resolution) equipped with a DH-2000 Deuterium-Halogen Light Source and the electrodes were connected to a Biologic SP-300 potentiostat.

Solvents and reagents

Anhydrous MeCN was purchased from Sigma-Aldrich, opened and stored in an Ar-filled glovebox. Smaller amounts of MeCN-d_3 and THF-d_8 were purchased and submitted to 3 freeze-pump-thaw cycles, vacuum distilled over CaH_2, introduced into an Ar-filled glovebox, and stored under activated 3 Å molecular sieves. We found this treatment to be enough for carrying out the low-valent bismuth chemistry described herein. Molecular sieves were activated at 250 °C under high vacuum for 1 day (pressure reading of vacuum line remained steady at 2×10^{-3} mbar for at least 5 h). Unless otherwise stated, solvents used for the preparation of the ligands, catalysts or starting materials were also anhydrous, but not degassed nor stored over molecular sieves prior to use. Dry ice/acetone baths were always prepared by adding a slow stream of acetone (via a squeeze / wash bottle stored in air) to a Dewar of dry ice, such as to avoid vigorous and uncontrolled bubbling of the solution.

Anhydrous $BiCl_3$ (99.9%, trace metal basis) was purchased from Alfa Aesar and stored in the glovebox. Unless otherwise noted, all reagents were obtained from commercial suppliers and used without further purification.

2. Light sources and photoreaction set-ups

Red-light irradiation

Red-light irradiation (for stoichiometric oxidative additions) was performed with two 660 nm Kessil lamps at full intensity. Unless mentioned otherwise, the reactions carried out under red-light irradiation were performed using two standardized 660 nm LED PR160L lamps purchased from Kessil.

The inside of the reactor box was fully covered with aluminum foil. The two Kessil lamps were assembled opposite to one another through two holes in the walls of the reactor. As suggested by the manufacturer, the two lamps were assembled at ca. 12 cm from each other. The reaction vessels will be then located at ca. 6 cm from each lamp, receiving an average intensity of 159 mW/cm^2 (see https://kessil.com/products/science_PR160L.php for light-intensity maps and other details). Unless stated otherwise, both PR160L Kessil lamps were used at the 100% intensity setting. The reactor was set up on top of a stirring plate, and the temperature of the reactions was maintained around 35 °C by using a cooling fan placed on top of the reactor.

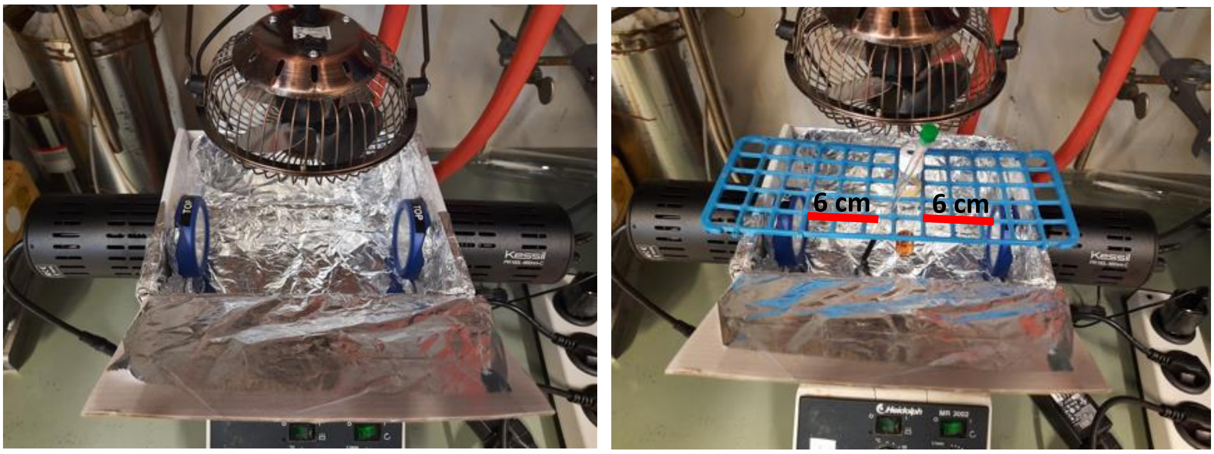

Figure S1. Setup with 2 × Kessil 660 nm LED PR160L lamps. The reactions sit at ca. 6 cm from each LED.

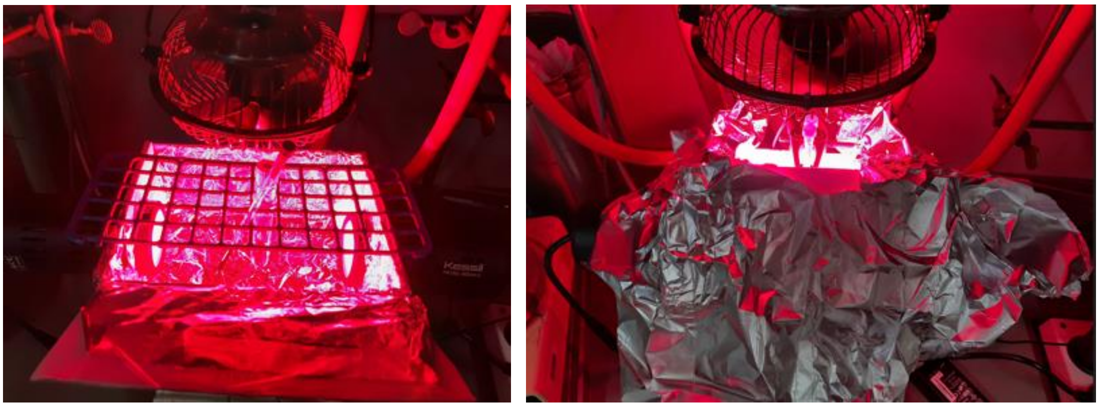

Figure S2. Kessil LED setup with lights on, uncovered (left) or covered with aluminum foil.

3. Synthesis of bismuthinidene **1a**

The bismuthinidene used in this study (**1a**) was prepared according to a scaled-up and modified version of a reported procedure, in three steps from the corresponding 2-bromoisophthalaldehyde **S1**.[1,2]

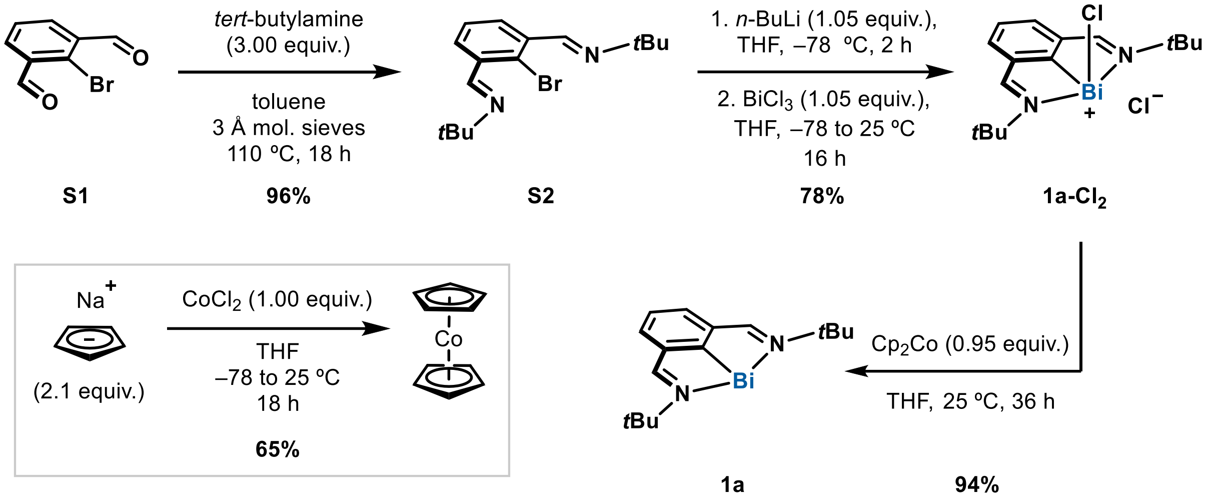

Synthesis of 2-bromo-1,3-bis(*N-tert*-butylmethanimino)benzene (S2)

In an argon-filled glovebox, a 500 mL flame-dried Schlenk bomb equipped with a Teflon-coated stir bar was charged with 5 g of activated 3Å mol sieves. The flask was ported out of the glovebox, connected to an argon/vacuum Schlenk line, and placed under an atmosphere of argon. To the flask was added 2-bromoisophthalaldehyde (4.00 g, 18.78 mmol, 1.00 equiv.) as a solid against a positive counterflow of argon. Anhydrous and degassed toluene (100 mL) and *tert*-butylamine (5.90 mL, 56.33 mmol, 3 equiv.) were introduced against a positive counterpressure of argon. The Teflon pin on the Schlenk bomb was sealed and the flask was lowered into an oil bath previously heated to 110 °C on a magnetic stirplate. The reaction was stirred vigorously (1200 rpm) at this temperature for 18 h. The reaction was allowed to cool to room temperature before being filtered over Celite, ensuring to wash the filter cake with 2 × 20 mL toluene. The filtrate was concentrated, first via rotary evaporation and then under high vacuum, to afford the desired product as an off-white powder (5.81 g, 96% yield). **^{1}H NMR** (300 MHz, $CDCl_3$) δ 9.61 (s, 2H), 8.17 (d, *J* = 7.5 Hz, 2H), 7.86 (m, 1H), 1.60 (s, 18H).

Synthesis of dichlorobismuthine (1a-Cl$_2$)

A flame-dried 250 mL Schlenk flask equipped with a Teflon-coated stir bar was charged with **S2** (3.00 g, 9.28 mmol, 1.00 equiv.) as a solid against a positive counterflow of argon. This was complemented with anhydrous and degassed THF (150 mL), and the contents of the flask were stirred to afford a homogeneous pale-yellow solution. The resulting solution was cooled to −78 °C with the aid of a dry ice/acetone bath, and to the stirring, cooled solution was added *n*-BuLi (3.90 mL of a 2.50 M solution in hexanes, 9.74 mmol, 1.05 equiv.) in a dropwise fashion over 5 min. During this addition, the solution turned orange. The resulting solution was allowed to stir at −78 °C for 2 h, during which a white precipitate started to form. A separate flame-dried 500 mL Schlenk flask equipped with a Teflon-coated stir bar was charged with anhydrous $BiCl_3$ (3.07 g, 9.74 mmol, 1.05 equiv.) against a positive counterflow of argon and complemented with anhydrous and degassed THF (90 mL) before being

cooled to −78 °C under an atmosphere or argon with the aid of a dry ice/acetone bath. The aryl lithium suspension (−78 °C) was transferred via cannula to the $BiCl_3$ solution (−78 °C) in one portion. Upon mixing, the resulting suspension turned bright yellow. After allowing this solution to stir at −78 °C for 5 min, the cooling bath was removed and the reaction was allowed to stir at room temperature for 16 h. *Note*: after removal of the cooling bath, the suspension changed from bright yellow to lime over the course of 1 h. The resulting solution was transferred to a 500 mL flask under air and concentrated via rotary evaporation. The residue was resuspended in 150 mL HPLC-grade CH_2Cl_2 and filtered over a pad of Celite that was pre-wetted with CH_2Cl_2. The residue was washed with 2 × 50 mL of CH_2Cl_2 and concentrated to dryness. The residue was dissolved in ca. 80 mL CH_2Cl_2 and the product crashed out by adding 100 mL of hexane. The suspension was filtered over a medium-fritted funnel and the residue washed with 100 mL of hexane. Drying the residue on high vacuum afforded the desired product as a white amorphous powder (3.79 g, 78% yield). **^{1}H NMR** (300 MHz, $CDCl_3$) δ 9.61 (s, 2H), 8.17 (d, *J* = 7.5 Hz, 2H), 7.85 (dd, *J* = 7.8, 7.2 Hz, 1H), 1.60 (s, 18H).[1,2]

Synthesis of bismuthinidene (1a)

While working in an argon-filled glovebox, a flame-dried 100 mL Schlenk flask equipped with a Teflon-coated stir bar under argon was added **1a-Cl$_2$** (1.00 g, 1.91 mmol, 1.00 equiv.) against a positive counterflow of argon. This was complemented with anhydrous and degassed THF (25 mL), and the contents of the flask were stirred vigorously to form a fine suspension. To the stirring suspension was added cobaltocene (343 mg, 1.82 mmol, 0.95 equiv.) in one portion. The Schlenk flask was hermetically sealed, ported out of the glovebox, and connected to an argon/vacuum Schlenk double manifold. The contents of the flask were kept under an atmosphere of argon and vigorously stirred (> 1200 rpm) for 36 h at room temperature. *Note*: during this time, the fluid of the suspension changed from a dark purple to a dark green color. The solvent was removed under high vacuum (high vacuum applied until pressure reading on vacuum gauge stayed at 3×10^{-3} mbar for at least 1 h), and the Schenk bomb was sealed and ported into an argon-filled glovebox. The crude material was suspended in anhydrous and degassed pentane (15 mL) and filtered through a pad of Celite (pre-wetted with pentane) over a fritted glass funnel under the protection of argon into a flame-dried Schlenk bomb, ensuring to rinse the reaction flask and the Celite pad with anhydrous and degassed pentane until the eluent was colorless (total ~50 mL of pentane). The receiving Schlenk bomb was hermetically sealed by closing the Teflon plug valve and ported out of the glovebox. The dark green pentane solution was cooled to −78 °C before subjecting the cooled, quiescent solution to high vacuum to slowly remove the pentane, using the latent heat of vaporization to keep the solution cold. *Note*: the solution should be slowly introduced to high vacuum to prevent vigorous degassing of the solution. Crystallization of the desired dark green compound accompanies the removal of the pentane. After complete removal of pentane, the resulting crude material was heated to 70 °C in a sand bath and left under high vacuum until the pressure reading on the vacuum gauge stayed at 1×10^{-3} mbar for at least 30 min, thus ensuring complete removal of any excess traces of pentane and Cp_2Co. This afforded **1a** as a dark green crystalline solid that was pure by ^{1}H NMR (1.16 g, 94%). The solid was stored at room temperature in an argon-filled glovebox. **^{1}H NMR** (300 MHz, THF-*d$_8$*) δ 9.81 (s, 2H), 7.94 (d, *J* = 7.4 Hz, 2H), 7.08 (t, *J* = 7.4 Hz, 1H), 1.57 (s, 18H). **^{1}H NMR** (300 MHz, MeCN-*d$_3$*) δ 9.88 (s, 2H), 8.05 (d, *J* = 7.4 Hz, 2H), 7.20 (t, *J* = 7.4 Hz, 1H), 1.57 (s, 18H).[1,2]

The above protocol could be scaled up to 2 g of **1a-Cl₂** (extending the reaction time to 48 h) to give 86% yield of the desired product. A synthesis of cobaltocene is detailed below (*vide infra*).

Synthesis of cobaltocene

A flame-dried 300 mL Schlenk flask equipped with a Teflon-coated stir bar was charged with NaCp (7.10 g, 80.9 mmol, 2.10 equiv.), followed by anhydrous and degassed THF (200 mL). The resulting mixture was stirred to afford a homogeneous light brown solution. A separate flame-dried 1 L 2-neck flask equipped with a Schlenk adaptor and a Teflon-coated stir bar was charged with $CoCl_2$ (5.00 g, 38.5 mmol, 1.00 equiv.) and anhydrous and degassed THF (250 mL). The resulting suspension was stirred vigorously and cooled to −78 °C with the aid of a dry ice/acetone bath. The NaCp solution was transferred via cannula to the cooled $CoCl_2$ solution as a gentle trickle over 5 min. After complete transfer of the NaCp solution, the receiving flask was removed from the dry ice/acetone bath and the reaction was stirred at room temperature overnight (18 h). The contents of the flask were concentrated to dryness on high vacuum and the flask refilled with argon. The flask was then fitted with a cold finger inserted through one of the necks of the flask against a positive counterflow of argon. The contents of the flask were placed under high vacuum for 5 min at room temperature, after which the cold finger was cooled to −35 °C (with the aid of a recirculating chiller) and the flask lowered into a 70 °C oil bath. The sublimation was allowed to proceed for 6 h, after which the flask was hermetically sealed, ported into the glovebox and the dark purple prismatic crystals of cobaltocene (4.70 g, 65%) were carefully scraped off the sublimation finger and stored at room temperature in an argon-filled glovebox.

4. Synthesis of aryl iodides

4.1. Synthesis of 1-(allyloxy)-2-iodo-benzene (**4**)

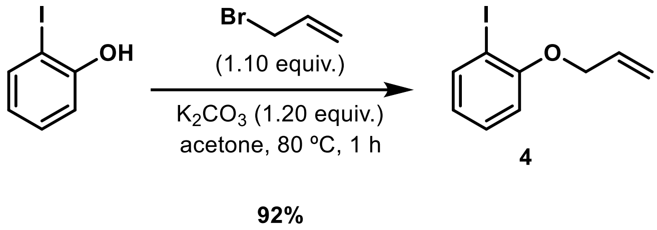

A flame-dried 10 mL Schlenk bomb equipped with a Teflon-coated magnetic stir bar was charged with 2-iodophenol (660 mg, 3.00 mmol, 1.00 equiv.), anhydrous K_2CO_3 (498 mg, 3.6 mmol, 1.2 equiv.), anhydrous acetone (2 mL), and allyl bromide (399 mg, 3.30 mmol, 1.10 equiv.) against a positive counterflow of argon. The Schlenk bomb was hermetically sealed by closing the Teflon plug valve, after which the flask was placed in an oil bath set to 80 °C, with stirring set to 1000 rpm. After 1 h, TLC of a quenched reaction aliquot (100% pentane) revealed complete consumption of the starting material and formation of one main species. The reaction was allowed to cool to room temperature and concentrated to dryness under high vacuum. The desired product was extracted with MTBE (2 × 50 mL) and the combined organic layers washed with 10% KOH (2 × 50 mL), water (50 mL), and brine (100 mL), before being dried over $MgSO_4$. The solvent was removed via rotary evaporation (40 °C, 20 mbar) to afford the desired product as a clear oil (780 mg, 92%). **^{1}H NMR** (300 MHz, $CDCl_3$) δ 7.78 (dd, *J* = 7.8, 1.6 Hz, 1H), 7.34 – 7.22 (m, 1H), 6.81 (dd, *J* = 8.3, 1.3 Hz, 1H), 6.71 (td, *J* = 7.6, 1.4 Hz, 1H), 6.06 (ddt, *J* = 17.3, 10.7, 4.8 Hz, 1H), 5.52 (dq, *J* = 17.3, 1.6 Hz, 1H), 5.31 (dq, *J* = 10.5, 1.4 Hz, 1H), 4.60 (dt, *J* = 4.9, 1.7 Hz, 2H).

4.2. Synthesis of 2,5-diiodo-1,3-dimethylbenzene (**6**)

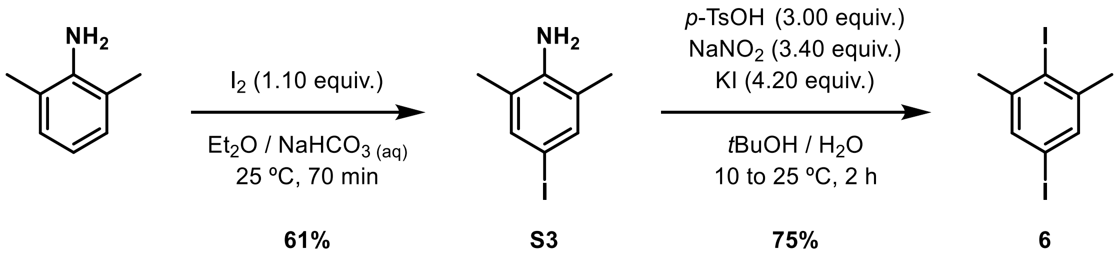

Synthesis of 4-iodo-2,6-dimethyl aniline (**S3**)

A 250 mL round-bottom flask open to air and equipped with a Teflon-coated stir bar was charged with 2,6-dimethylaniline (2.00 g, 16.50 mmol, 1.00 equiv.), inhibitor-free diethyl ether (15 mL), and a saturated aqueous solution of $NaHCO_3$ (45 mL). The resulting biphasic mixture was stirred vigorously at room temperature (> 1200 rpm) before being complemented with iodine (4.60 g, 18.2 mmol, 1.10 equiv.) in one portion. TLC analysis (15% EtOAc in pentane) after 70 min revealed full conversion of

the starting material (R_f = 0.65) and formation of one major species (R_f = 0.50). The reaction mixture was poured into a separatory funnel containing a saturated aqueous solution of sodium thiosulfate (30 mL). The aqueous phase was extracted with MTBE (2 × 100 mL), and the combined organic layers were washed with water (2 × 100 mL), brine (1 × 150 mL), and dried over $MgSO_4$ before being concentrated via rotary evaporation to afford a dark red oil. The crude was dissolved in a minimal amount of pentane and cooled to −20 °C for 15 min, after which a precipitate formed at the bottom of the flask. The mother liquor was decanted and the residue triturated with cold pentane (2 × 5 mL) to afford the desired product as a light burgundy solid (2.74 g, 61%) that was used in the subsequent step without further purification. **^{1}H NMR** (300 MHz, $CDCl_3$) δ 7.24 (s, 2H), 3.57 (s, 2H), 2.13 (s, 6H).

Synthesis of 2,5-diiodo-1,3-dimethylbenzene (**6**)

A 250 mL round-bottom flask equipped with a Teflon-coated stir bar was charged with *p*-TsOH·H_2O (4.62 g, 24.30 mmol, 3.00 equiv.) and *t*BuOH (32 mL). The contents of the flask were stirred at room temperature to afford a homogeneous solution. The stirring solution was complemented with 2,6-dimethyl-4-iodoaniline (2.00 g, 8.10 mmol, 1.00 equiv.) in one portion at room temperature. The resulting suspension of anilinium tosylate was cooled to 10–15 °C with the aid of an ice/water bath. To the stirring reaction was added a solution of $NaNO_2$ (1.12 g, 16.2 mmol, 2 equiv.) and KI (3.36 g, 20.2 mmol, 2.5 equiv.) in H_2O (5 mL) in a dropwise fashion over 10 min. After complete addition the reaction mixture was stirred at 10 °C for 10 min before being warmed up to room temperature. After stirring at room temperature for 1 h, TLC analysis revealed incomplete conversion of the starting material. A solution of $NaNO_2$ (0.77 g, 11.1 mmol, 1.38 equiv.) and KI (2.3 g, 13.8 mmol, 1.7 equiv.) in H_2O (5 mL) was added to the flask in a dropwise fashion over 5 min at room temperature. The reaction mixture was allowed to react for an additional 1 h, after which TLC analysis revealed complete consumption of the starting material. The reaction mixture was complemented with a saturated aqueous solution of $NaHCO_3$ (100 mL) and $Na_2S_2O_3$ (45 mL), and the product was extracted with EtOAc (2 × 150 mL). The combined organic phases were washed with 1 M HCl (1 × 100 mL), $NaHCO_3$ (2 × 100 mL), a saturated aqueous solution of NH_4Cl (1 × 150 mL), before being dried over $MgSO_4$ and concentrated to give a reddish/brown amorphous solid. The crude solid was dissolved in a minimal amount of pentane, loaded on a short plug of silica (ca. 20 g) and flushed with *ca.* 250 mL of pentane to elute the desired product. The eluent was concentrated to dryness to afford the desired product as a crystalline off-white solid (2.18 g, 75%). **^{1}H NMR** (300 MHz, $CDCl_3$) δ 7.38 (h, *J* = 0.6 Hz, 2H), 2.41 (t, *J* = 0.6 Hz, 6H).

4.3. Synthesis of 2,5-diiodo-1,3-diisopropylbenzene (**8**)

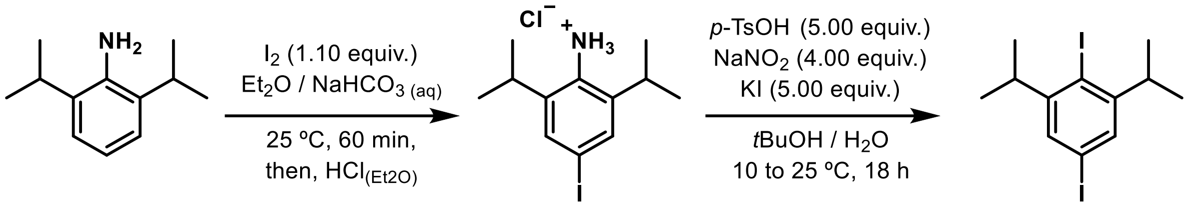

89% **S4** **89%** **8**

Synthesis of 4-iodo-2,6-diisopropylanilinium chloride (**S4**)

A 250 mL round-bottom flask open to air and equipped with a Teflon-coated stir bar was charged with 2,6-diisopropylaniline (2.00 g, 11.3 mmol, 1.00 equiv.) inhibitor-free diethyl ether (15 mL), and a saturated aqueous solution of $NaHCO_3$ (30 mL). The resulting biphasic mixture was stirred vigorously at room temperature (> 1200 rpm) before being complemented with iodine (3.15 g, 12.4 mmol, 1.10 equiv.) in one portion. TLC analysis (5% EtOAc in pentane) after 1 h showed full conversion of the starting material. The reaction mixture was poured into a separatory funnel containing a saturated aqueous solution of sodium thiosulfate (30 mL). The aqueous phase was extracted with MTBE (2 × 100 mL), and the combined organic layers were washed with water (2 × 100 mL), brine (1 × 150 mL), and dried over $MgSO_4$ before being concentrated via rotary evaporation to afford a dark red oil. The oil was subjected to high vacuum complemented with anhydrous Et_2O (10 mL), followed by HCl (8.5 mL of a 2M solution in Et_2O, 1.5 equiv.), leading to precipitation of the anilinium chloride. The solution was complemented with anhydrous pentane (10 mL) and cooled to 0 °C. The resulting suspension was filtered and the residue washed with 50 mL of a 1:1 mixture of Et_2O and pentane. The resulting solid was dried under high vacuum to yield the desired anilinium chloride salt as an amorphous white powder (3.42 g, 89%). **^{1}H NMR** (300 MHz, $CDCl_3$) δ 10.39 (s, 3H), 7.53 (s, 2H), 3.61 (hept, J = 6.4 Hz, 2H), 1.27 (d, J = 6.7 Hz, 12H).

Synthesis of 2,5-diiodo-1,3-diisopropylbenzene (**8**)

A 250 mL round-bottom flask equipped with a Teflon-coated stir bar was charged with *p*-TsOH·H_2O (4.57 g, 24.0 mmol, 3.00 equiv.) and *t*BuOH (32 mL). The contents of the flask were stirred at room temperature to afford a homogeneous solution. The stirring solution was complemented with 2,6-diisopropyl-4-iodoaniline (2.40 g, 8.00 mmol, 1.00 equiv.). The resulting suspension of anilinium tosylate was cooled to 10–15 °C with the aid of an ice/water bath. To the stirring reaction was added a solution of $NaNO_2$ (1.10 g, 16.0 mmol, 2.00 equiv.) and KI (3.32 g, 20.0 mmol, 2.50 equiv.) in H_2O (5 mL). After complete addition the reaction mixture was stirred at 10 °C for 10 min before being warmed up to room temperature. After stirring at room temperature for 1 h, TLC analysis revealed incomplete consumption of starting material. A solution of $NaNO_2$ (0.77 g, 11.1 mmol, 1.38 equiv.) and KI (2.30 g, 13.8 mmol, 1.70 equiv.) in H_2O (5 mL) was added to the flask in a dropwise fashion over 10 min at room temperature. The resulting reaction was allowed to stir overnight, after which TLC analysis (100%

pentane) of an aliquot worked up with aqueous $NaHCO_3$ and extracted with ethyl acetate revealed no remaining starting material (R_f = 0.08), and formation of one major product (R_f = 0.8), corresponding to the desired product. The crude reaction was poured into a separatory funnel containing a saturated aqueous solution of sodium thiosulfate (100 mL), and the reaction flask rinsed with water and pentane. The product was extracted with pentane (2 × 150 mL), and the combined organic layers were washed with 1 M HCl (1 × 100 mL), saturated aqueous $NaHCO_3$ (2 × 100 mL), water (1 × 100 mL), and brine (1 × 100 mL), before being dried over $MgSO_4$ and concentrated to give a viscous red oil. This residue was purified by flash chromatography on silica gel (100% pentane) and the appropriate fractions concentrated, first via rotary evaporation and then high vacuum, to afford the desired product as a colorless viscous oil (2.96 g, 89%). **^{1}H NMR** (300 MHz, $CDCl_3$) δ 7.35 (s, 2H), 3.34 (hept, *J* = 6.8 Hz, 2H), 1.21 (d, *J* = 6.8 Hz, 12H).

4.4. Synthesis of 2-iodo-1,3-diisopropylbenzene (**10**)

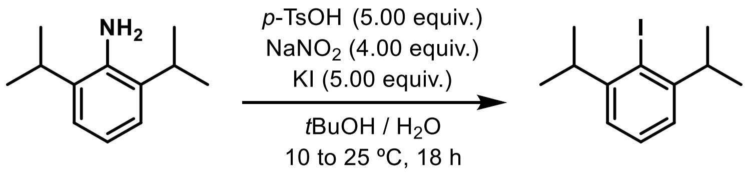

67% **10**

Synthesis of 2-iodo-1,3-diisopropylbenzene (**10**)

A 250 mL round-bottom flask equipped with a Teflon-coated stir bar was charged with *p*-TsOH·H_2O (4.57 g, 24.0 mmol, 3.00 equiv.) and *t*BuOH (32 mL). The contents of the flask were stirred at room temperature to afford a homogeneous solution. The stirring solution was complemented with 2,6-diisopropylaniline (1.42 g, 8.00 mmol, 1.00 equiv.). The resulting suspension of anilinium tosylate was cooled to 10-15 °C with the aid of an ice/water bath. To the stirring reaction was added a solution of $NaNO_2$ (1.10 g, 16.0 mmol, 2.00 equiv.) and KI (3.32 g, 20.0 mmol, 2.50 equiv.) in H_2O (5 mL). After complete addition the reaction mixture was stirred at 10 °C for 10 min before being warmed up to room temperature. After stirring at room temperature for 1 h, TLC analysis revealed incomplete consumption of starting material. A solution of $NaNO_2$ (1.10 g, 16.0 mmol, 2.00 equiv.) and KI (3.32 g, 20.0 mmol, 2.50 equiv.) in H_2O (5 mL) was added to the flask in a dropwise fashion over 10 min at room temperature, followed by additional *p*-TsOH·H_2O (3.01 g, 16.0 mmol, 2.00 equiv.). The resulting reaction was allowed to stir overnight, after which TLC analysis (100% pentane) revealed no remaining starting material. The crude reaction was poured into a separatory funnel containing a saturated aqueous solution of sodium thiosulfate (100 mL), ensuring to rinse out the reaction flask with water and pentane. The product was extracted with pentane (2 × 150 mL), and the combined organic layers were washed with 1 M HCl (1 × 100 mL), saturated aqueous $NaHCO_3$ (2 × 100 mL), water (1 × 100 mL), and brine (1 × 100 mL), before being dried over $MgSO_4$ and concentrated to give a reddish/brown solid. Purification of the crude material by flash chromatography on silica gel (100% pentane), followed by concentration of the desired fractions, first via rotary evaporation and then high vacuum, afforded the

desired product as a colorless oil that solidified upon cooling to room temperature (1.56 g, 67%). **^{1}H NMR** (300 MHz, $CDCl_3$) δ 7.24 (dd, *J* = 8.2, 7.0 Hz, 1H), 7.08 (d, *J* = 7.6 Hz, 2H), 3.41 (hept, *J* = 6.8 Hz, 2H), 1.24 (d, *J* = 6.8 Hz, 12H).

5. Kinetic Experiments

5.1. LED-NMR Kinetics Measurements

The Ultra-High-Power red LED (UHP-T-630-SR, 630 nm) was controlled using a UHPTLCC-02 Benchtop Current Controller. A High NA Optical Fiber (plastic optical fiber (POF), core diameter 1000 μm, 6 m length), was attached to the LED using a UHPTLCC-02 Fiber Coupling Adaptor. All components were produced by Prismatix and purchased through Mountain Photonics. The other end of the fiber optic cable inserted into a Wilmad 5 mm Screw Cap NMR equipped with a coaxial insert, the latter of which houses the fiber optic cable. The power at the tip of the fiber optic can be controlled between 0 and 950 mW at the collimation point. This setup is portable and can be easily moved between different spectrometers.

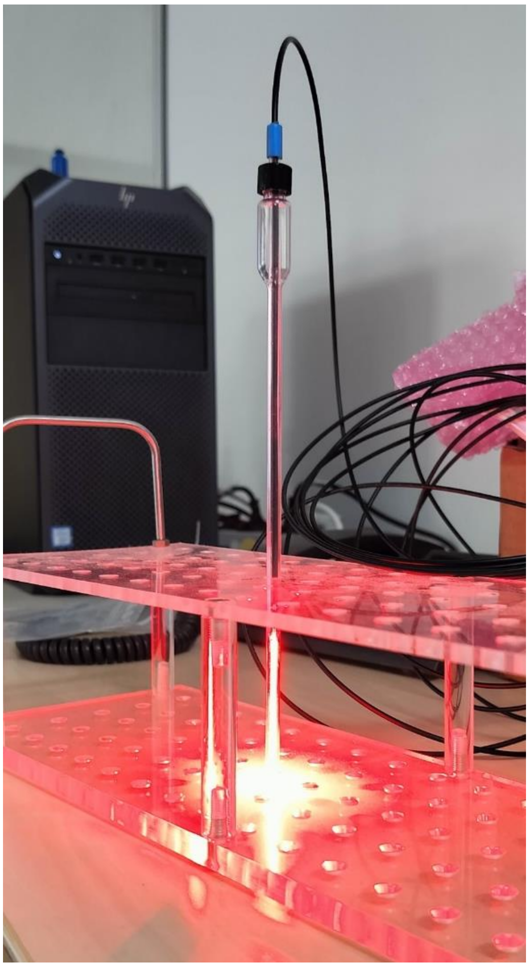

Figure S3: Fully assembled LED-NMR apparatus (without sample), with light intensity at 50% power.

The last few centimeters of the coaxial glass insert are tapered to allow for more sample to be contained in the sample tube. The last few centimeters of the fiber optic cable are sandblasted to ensure radially uniform light irradiation from the center of the sample. Despite the advantages of this setup, calculating an exact photon flux through the sample is unfeasible. Thus, light intensity was modulated from the power unit, assuming a linear relationship between the power at the collimation point and the number

of photons reaching the sample. The coaxial glass insert is passed through a rubber-lined screw cap at the top of the sample tube. Upon closure of the screw cap, an airtight rubber/glass seal is formed during expansion of the rubber in the septum.

A red light-promoted reaction was conducted between **1a** and **2** in a culture tube using the Kessil light setup described in Section 2. At t = 2, 4, and 6 h, a dual laser-targeted infrared thermometer was used to measure the temperature of the outside glass of the culture tube. This gave readings of 34.4, 35.1, and 34.1 °C (average = 34.5 ± 0.42 °C). To better emulate the experimental conditions during Kessil light irradiation, a constant temperature of 35.0 °C was maintained during the *in situ* LED-NMR time courses using the active temperature control capabilities of NMR instrument.

5.2. Representative time course (**1a** + **2**)

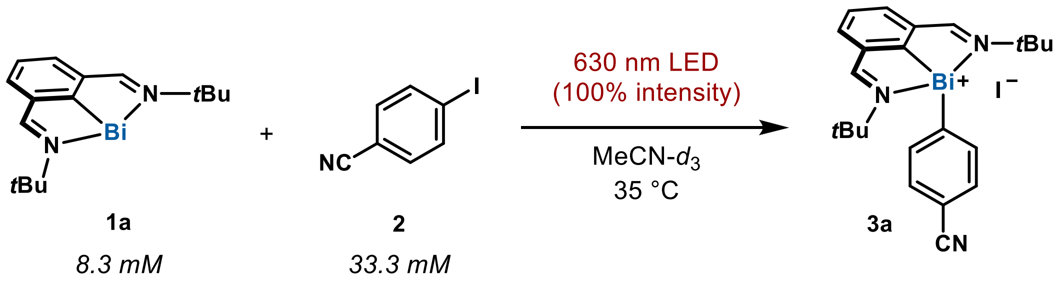

For kinetic studies, *p*-iodobenzonitrile (**2**) was purified as follows: a commercial sample of compound **2** (2 g) was recrystallized from a saturated hot solution of 4:1 EtOH/MeOH (12 mL total, heated to just below boiling point). After allowing the hot solution to cool down to room temperature, the product was obtained as clear, colorless, rod-like crystals whose edges diffracted incident light. The mother liquor was decanted and the crystals washed with cold (~ 0 °C) 4:1 EtOH/MeOH (2 × 10 mL). The crystals were collected and dried under high vacuum (final pressure reading stayed at 2×10^{-3} mbar for 1 h).

General procedure for LED-NMR kinetics experiments

While working in an argon-filled glovebox, a 4 mL vial was charged with **1a** (7.50 mg, 1.00 equiv.), **2** (15.3 mg, 4.00 equiv.), and trimethoxybenzene (5.60 mg, 2.00 equiv.) in MeCN-d_3 (2 mL). Using a precision pipette, a 400 μL aliquot of the resulting solution was transferred to an NMR tube fitted with a screw-cap top. The NMR tube was sealed with a rubber-lined screw cap, through which was inserted a coaxial inner glass insert, thus creating an air-tight seal between the outer wall of the glass insert and the rubber lining of the screw cap. The connection at the NMR tube / screw cap / insert junction was further secured with parafilm and the setup was removed from the glovebox. The coaxial inner cell was fitted with a fiber optic cable featuring a sandblasted tip. The opposite end of the fiber optic cable was connected to a high-power LED light source and the sample placed in the NMR instrument. The sample was actively heated to 35 °C, after which the instrument was locked to the deuterated solvent signal, tuned, and shimmed. Irradiation of the sample (Ultra-High Power LED light, 630 nm) and data collection were commenced simultaneously.

Characterization data for **3a**:

^{1}H NMR (600 MHz, CD_3CN) δ 9.76 (s, 2H), 8.34 (d, *J* = 7.6 Hz, 2H), 8.28 – 8.23 (m, 2H), 8.09 (dd, *J* = 7.8, 7.4 Hz, 1H), 7.81 – 7.75 (m, 2H), 1.30 (s, 18H).

^{13}C NMR (151 MHz, CD_3CN) δ 188.98, 185.53, 168.91, 149.51, 139.62, 137.70, 135.81, 131.82, 119.51, 113.44, 62.43, 31.01.

Illustrative NMR stack plot of an LED-NMR kinetics run

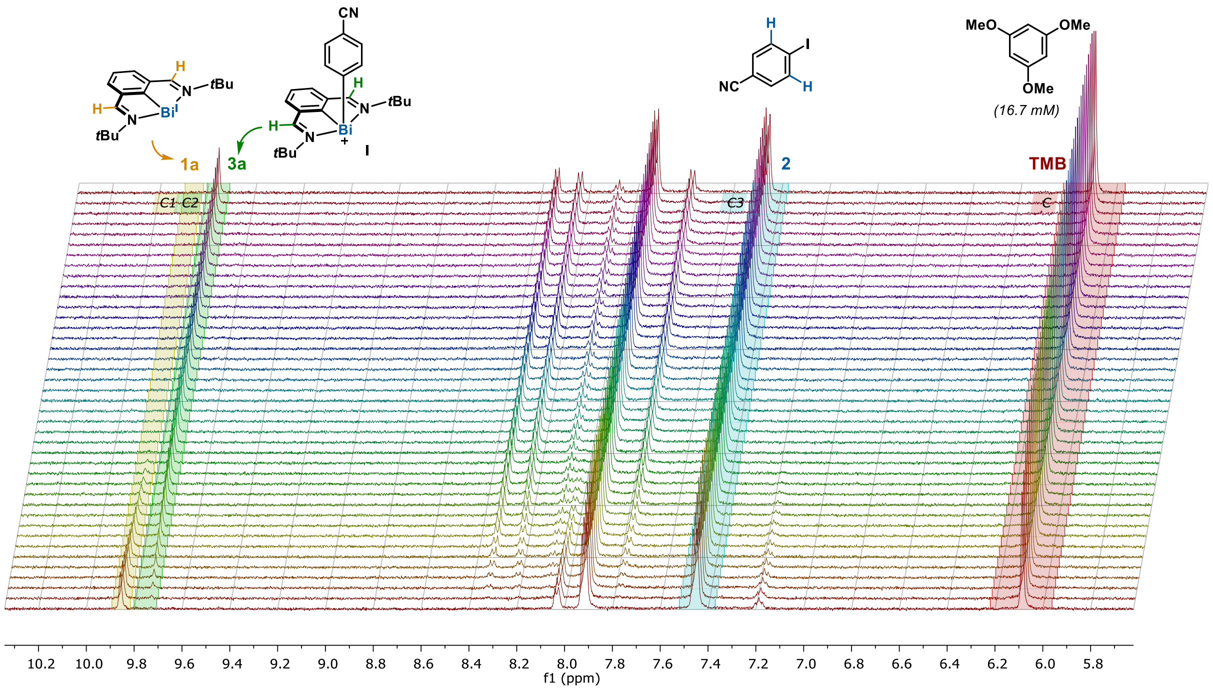

Figure S4: Stacked spectra for continuous ^{1}H NMR monitoring of the red light-promoted reaction between **1a** and **2** using an in situ LED-NMR setup. The highlighted peaks were used to construct plots of concentration vs time for the various reaction components, using 1,3,5-trimethoxybenzene as a true internal standard. The peaks in question, in order of decreasing chemical shift value, correspond to the starting bismuthinidene **1a** (9.87 ppm, beige band, aldimine protons), the oxidative addition product **3a** (9.76 ppm, green band, aldimine protons), aryl iodide **2** (7.47 ppm, light blue band), 1,3,5-trimethoxybenzene (6.10 ppm, red band). The structures of each species are included and the protons responsible for the diagnostic peaks are highlighted accordingly.

Representative plotted concentration vs time data for reaction between **1a** and **2**:

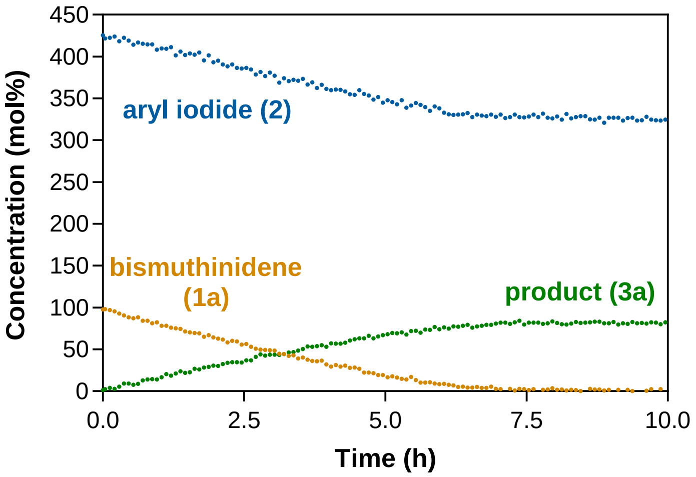

Figure S5: Representative full time course for the red light-promoted reaction between **1a** and **2**. Reaction conditions: [**1a**] = 8.3 mM, [**2**] = 33.2 mM, light intensity = 100% MeCN-d_3, 35 °C. Traces shows are the concentrations of **1a** (●), oxidative addition product **3a** (●), and **2** (●), calculated using 1,3,5-trimethoxybenzene as an internal standard.

Due to the long reaction times required for full NMR time course data (> 7 h), the NMR experiments were carried out to 15 – 20% conversion and the initial rates were obtained from linear regression on the Bi(I) consumption trace at ≤ 10% conversion. Along the same vein, only the data required to glean the order in light intensity was recorded in triplicate to showcase the reproducibility of the LED-NMR data.

5.3. Dependence on light intensity (0 – 100%)

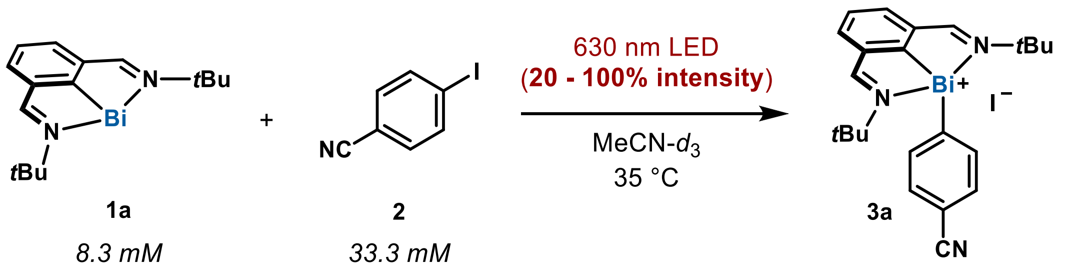

While working in an argon-filled glovebox, a 4 mL vial was charged with **1a** (7.5 mg, 1.0 equiv.), **2** (15.3 mg, 4.0 equiv.), and 1,3,5-trimethoxybenzene (5.6 mg, 2.0 equiv.) in MeCN-d_3 (2 mL). Using a precision pipette, a 400 μL aliquot of the resulting solution was transferred to an NMR tube fitted with a screw-cap top. The NMR tube was sealed with a rubber-lined screw cap, through which was inserted a coaxial inner glass insert, thus creating an air-tight seal between the outer wall of the glass insert and the rubber lining of the screw cap. The connection at the NMR tube / screw cap / insert junction was further secured with parafilm and the setup was removed from the glovebox. The coaxial inner cell was fitted with a fiber optic cable featuring a sandblasted tip. The opposite end of the fiber optic cable was connected to a high-power LED light source and the sample placed in the NMR instrument. The sample was actively heated to 35 °C, after which the instrument was locked to the deuterated solvent signal, tuned, and shimmed. Irradiation of the sample (Ultra-High Power LED light, 630 nm) and data collection were commenced simultaneously.

Data were collected in triplicate for LED-NMR reactions run 100%, 80%, 60%, 40%, and 20% light intensity, which was adjusted by the potentiometer of the power control unit. A reaction left in the dark at 35 °C in the NMR showed no conversion after 24 h.

Table S1: Recipes for LED-NMR reactions performed at various loadings aryl iodide **2**. The initial rates are obtained by linear regression of the **1a** consumption trace at ≤ 10% conversion.

Light intensity (%)	Initial rate (mM h^{-1}) ± st. dev.
0	0
20	0.34 ± 0.03
40	0.69 ± 0.01
60	0.97 ± 0.02
80	1.22 ± 0.003
100	1.55 ± 0.03

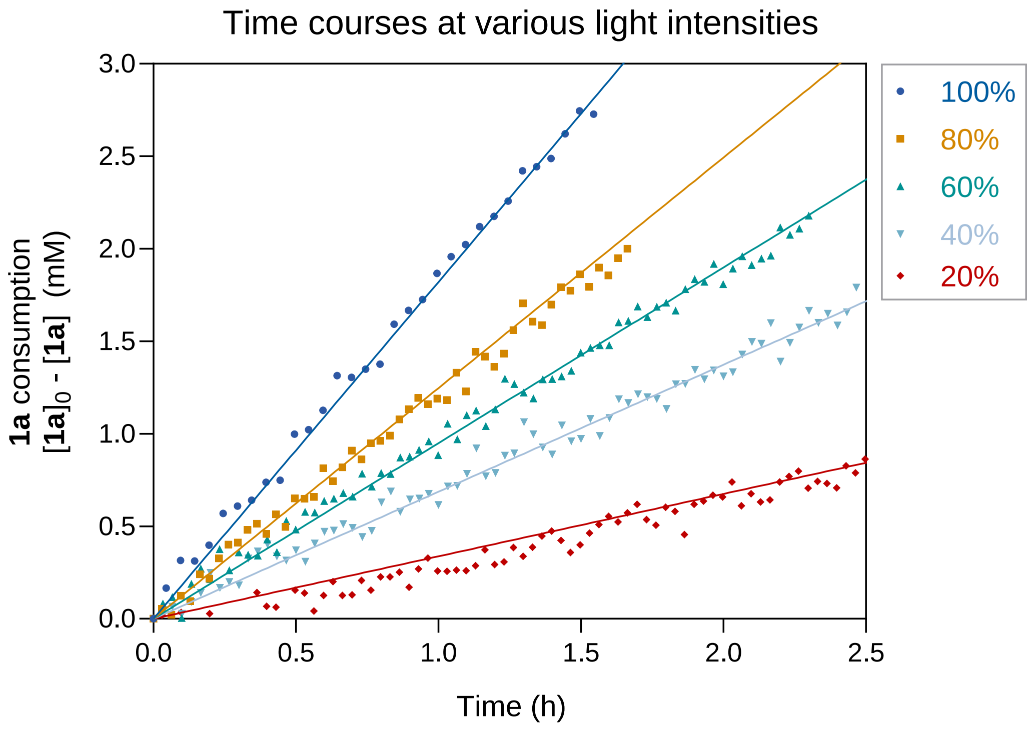

Figure S6: Representative time courses for the red light-promoted reaction between **1a** and **2** at varying light intensities. Reaction conditions: [**1a**] = 8.3 mM, [**2**] = 33.2 mM, light intensity = 20% (●), 40% (●), 60% (●), 80% (●), 100% (●), MeCN-d_3, 35 °C. Lines of best fit were calculated by linear regression of the **1a** consumption trace at ≤ 10% conversion.

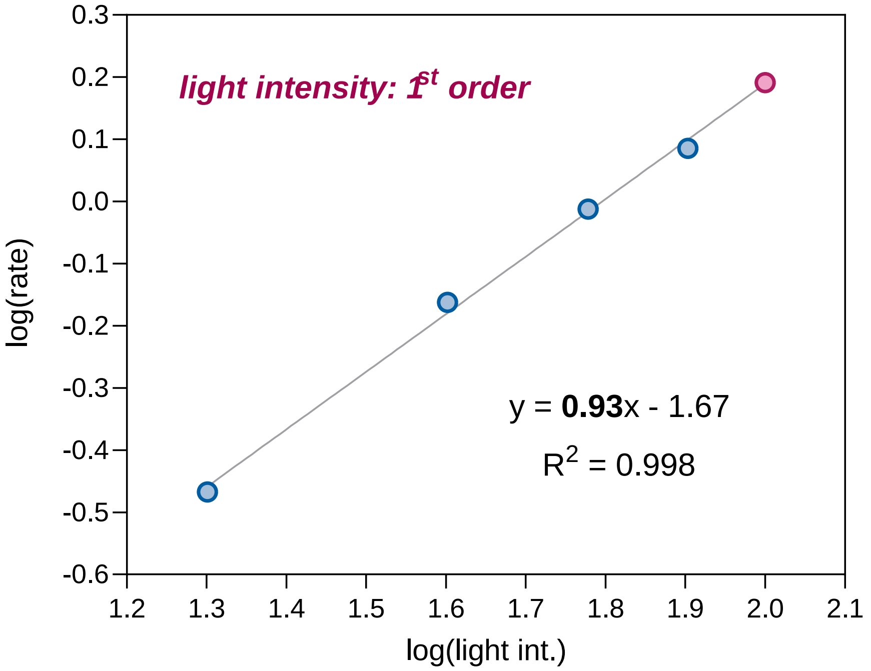

Figure S7: Log(rate) vs log(intensity) to calculate kinetic order in photon flux. Data shown in purple denote "standard" reaction conditions. Standard conditions: [**1a**] = 8.3 mM, [**2**] = 33.2 mM, light intensity = 100% MeCN-d_3, 35 °C.

5.4. Dependence on aryl iodide **2** (8.3 – 133.3 mM)

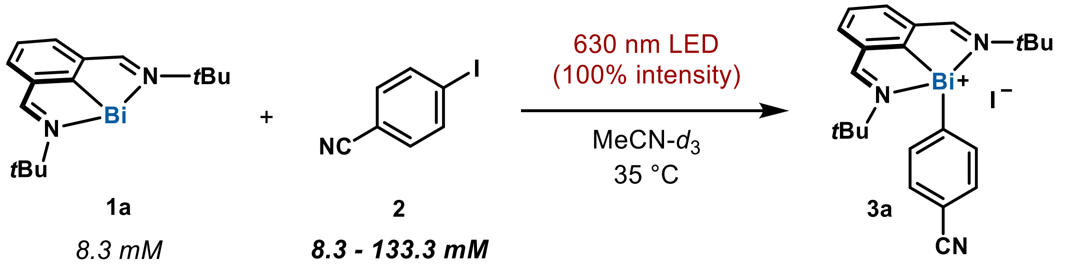

While working in an argon-filled glovebox, two stock solutions were prepared in two separate 4 mL scintillation vials:

Stock A: **1a** (15.1 mg), 1,3,5-trimethoxybenzene (11.2 mg), and MeCN-d_3 (1 mL)

Stock B: **2** (80.6 mg) in MeCN-d_3 (2 mL)

Using a precision pipette, stock solutions were mixed according to the following proportions for each experiment (categorized by concentration of **2**):

Table S2: Recipes for LED-NMR reactions performed at various loadings aryl iodide **2**. The initial rates are obtained by linear regression of the **1a** consumption trace at ≤ 10% conversion.

**[2] loading (mM)**	**Stock A (µL)**	**Stock B (µL)**	**MeCN-d_3 balance (µL)**	**Initial rate (mM h^{-1})**
**8.3**	100	19	281	**0.33**
**16.7**	100	38	262	**0.74**
**33.3**	100	75	225	**1.51**
**66.7**	100	150	150	**3.22**
**133.3**	100	300	0	**6.56**

For each experiment, all 400 µL of the resulting solution was transferred to an NMR tube fitted with a screw-cap top. The NMR tube was sealed with a rubber-lined screw cap, through which was inserted a coaxial inner glass insert, thus creating an air-tight seal between the outer wall of the glass insert and the rubber lining of the screw cap. The connection at the NMR tube / screw cap / insert junction was further secured with parafilm and the setup was removed from the glovebox. The coaxial inner cell was fitted with a fiber optic cable featuring a sandblasted tip. The opposite end of the fiber optic cable was connected to a high-power LED light source and the sample placed in the NMR instrument. The sample was actively heated to 35 °C, after which the instrument was locked to the deuterated solvent signal, tuned, and shimmed. Irradiation of the sample (Ultra-High Power LED light, 630 nm, 100% intensity) and data collection were commenced simultaneously.

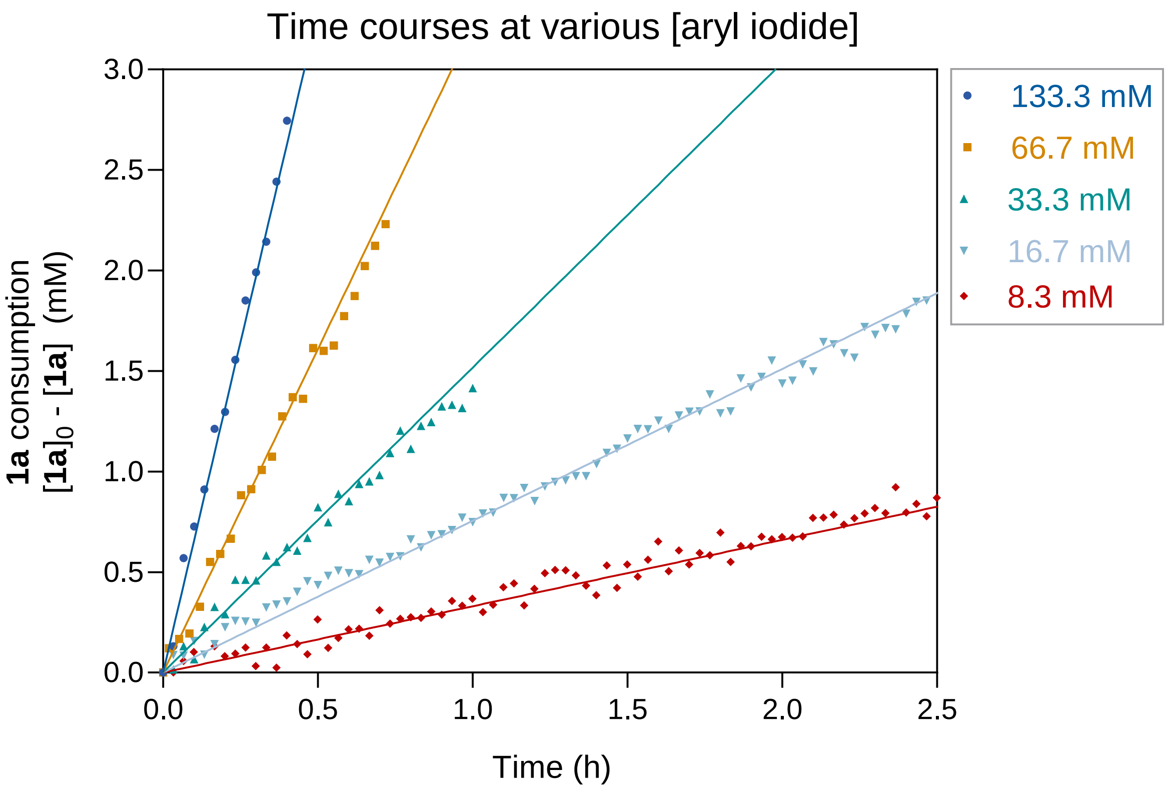

Figure S8: Time courses for the red light-promoted reaction between **1a** and **2** at varying loadings of aryl iodide **2**. Reaction conditions: [**1a**] = 8.3 mM, [**2**] = 8.3 mM (●), 16.7 mM (●), 33.3 mM (●), 66.7 mM (●), 133.3 mM (●), light intensity = 100%, MeCN-d_3, 35 °C. Lines of best fit were calculated by linear regression of the **1a** consumption trace at ≤ 10% conversion.

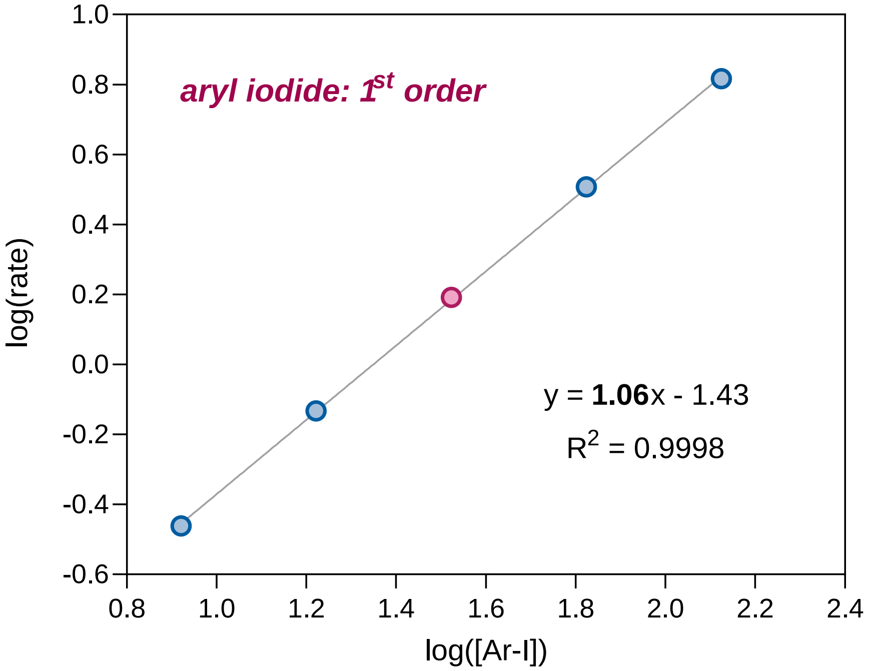

Figure S9: Plot of log(rate) vs log([**2**]) to calculate kinetic order in aryl iodide. Data shown in purple denote "standard" reaction conditions. Standard conditions: [**1a**] = 8.3 mM, [**2**] = 33.2 mM, light intensity = 100% MeCN-d_3, 35 °C.

5.5. Dependence on **1a** (2.1 – 33.3 mM)

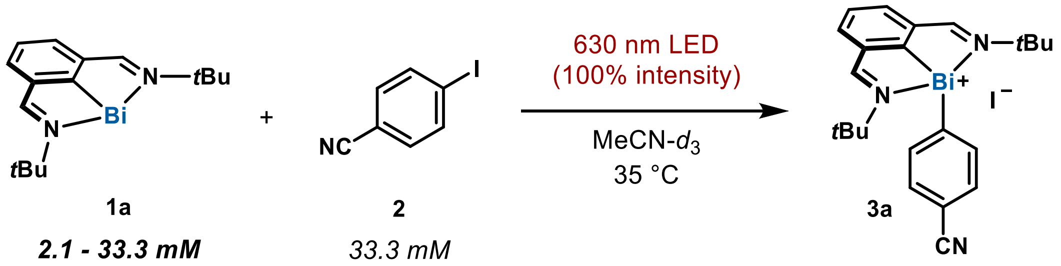

While working in an argon-filled glovebox, two stock solutions were prepared in two separate 4 mL scintillation vials:

Stock A: **2** (30.2 mg), 1,3,5-trimethoxybenzene (11.2 mg), and MeCN-d_3 (1 mL)

Stock B: **1a** (39.8 mg) in MeCN-d_3 (2 mL)

Using a precision pipette, the stock solutions were mixed according to the following proportions for each experiment (categorized by loading of **1a**):

Table S3. Recipes for LED-NMR reactions performed at various loadings of **1a**. The initial rates are obtained by linear regression of the **1a** consumption trace at ≤ 10% conversion.

[1a] loading (mM)	Stock A (μL)	Stock B (μL)	MeCN-d_3 balance (μL)	Initial rate (mM h^{-1})
**2.1**	100	19	281	**1.30**
**4.2**	100	38	262	**1.23**
**8.3**	100	75	225	**1.40**
**16.7**	100	150	150	**1.23**
**33.3**	100	300	0	**1.30**

For each experiment, all 400 μL of the resulting solution was transferred to an NMR tube fitted with a screw-cap top. The NMR tube was sealed with a rubber-lined screw cap, through which was inserted a coaxial inner glass insert, thus creating an air-tight seal between the outer wall of the glass insert and the rubber lining of the screw cap. The connection at the NMR tube / screw cap / insert junction was further secured with parafilm and the setup was removed from the glovebox. The coaxial inner cell was fitted with a fiber optic cable featuring a sandblasted tip. The opposite end of the fiber optic cable was connected to a high-power LED light source and the sample placed in the NMR instrument. The sample was actively heated to 35 °C, after which the instrument was locked to the deuterated solvent signal, tuned, and shimmed. Irradiation of the sample (Ultra-High Power LED light, 630 nm, 100% intensity) and data collection were commenced simultaneously.

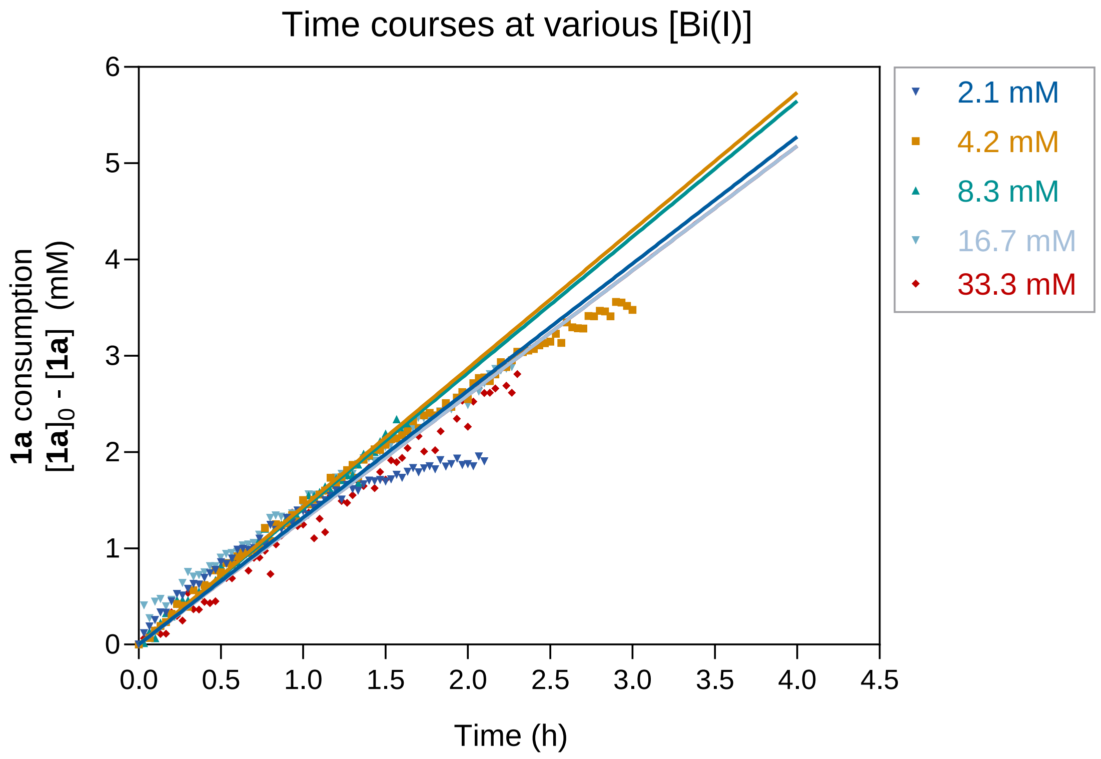

Figure S10: Time courses for the red light-promoted reaction between **1a** and **2** at varying loadings of bismuthinidene **1a**. Reaction conditions: [**1a**] = 2.1 mM (●), 4.2 mM (●), 8.3 mM (●), 16.7 mM (●),

33.3 mM (●), [**2**] = 33.3 mM, light intensity = 100%, MeCN-d_3, 35 °C. Lines of best fit were calculated by linear regression of the **1a** consumption trace at ≤ 10% conversion.

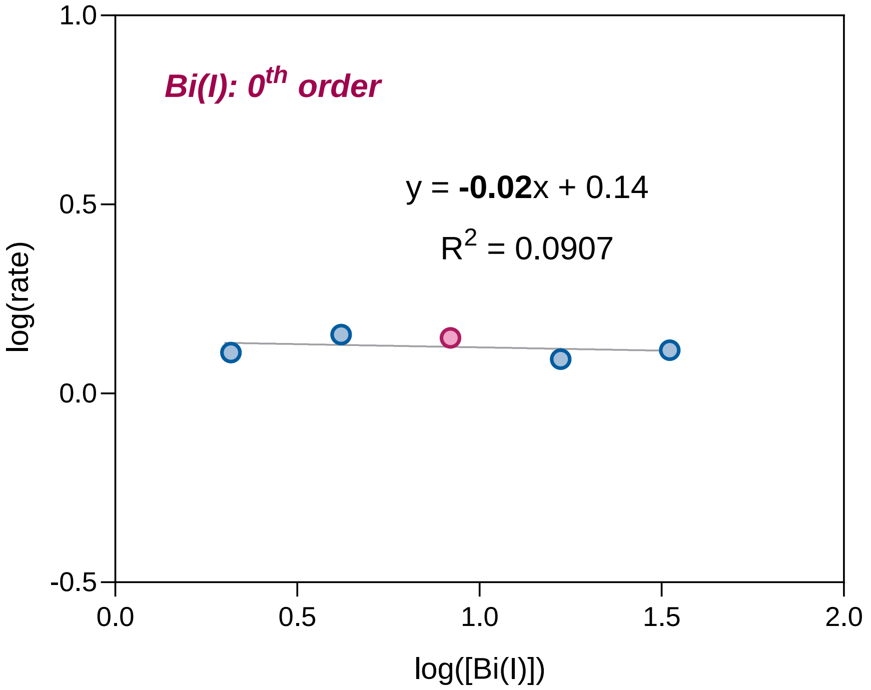

Figure S11: Plot of log(rate) vs log([**1a**]) to calculate kinetic order in bismuthinidene. Data shown in purple denote "standard" reaction conditions. Standard conditions: [**1a**] = 8.3 mM, [**2**] = 33.2 mM, light intensity = 100% MeCN-d_3, 35 °C.

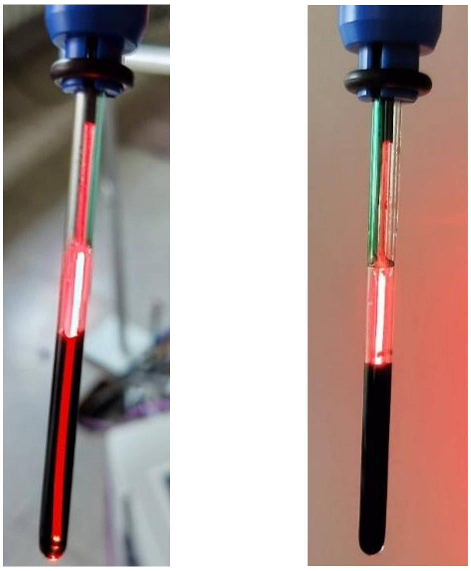

[1a] = 8.3 mM **[1a] = 16.7 mM**

Figure S12: Qualitative transmission through the sample at 8.3 mM (left) and 16.7 mM (right) of **1a** in the LED-NMR apparatus at 100% light intensity.

The poor transmission of light through the sample, even at low concentrations, helps rationalize the zeroth order in **1a** under the range of concentrations tested. The high molar absorptivity of the complex places the formation of the excited state in a light-limited regime.

5.6. Hammett plot

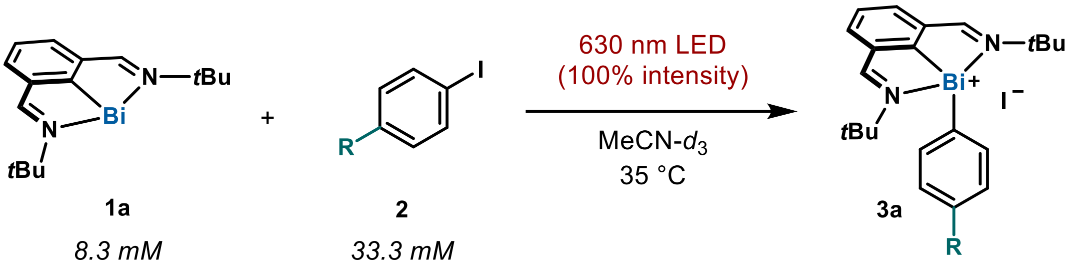

1a *8.3 mM* | **2** *33.3 mM* | **3a**

While working in an argon-filled glovebox, seven stock solutions were prepared in seven separate 4 mL scintillation vials:

Stock A: Bi(I) (15.1 mg), TMB (11.2 mg), and MeCN-d_3 (1 mL)

Stock B: 4-iodobenzonitrile **2** (10.2 mg) in MeCN-d_3 (1 mL)

Stock C: 1-iodo-4-(trifluoromethyl)benzene (12.1 mg) in MeCN-d_3 (1 mL)

Stock C: 1-chloro-4-iodobenzene (10.6 mg) in MeCN-d_3 (1 mL)

Stock D: 1-fluoro-4-iodobenzene (9.9 mg) in MeCN-d_3 (1 mL)

Stock E: 1-iodo-4-methylbenzene (9.7 mg) in MeCN-d_3 (1 mL)

Stock F: 1-iodo-4-methoxybenzene (10.4 mg) in MeCN-d_3 (1 mL)

Reaction solutions were prepared by mixing 100 µL of **stock A** and 300 µL of **stocks B-F** using a precision pipette, according to the aryl iodide tested. All 400 µL of the resulting solution was transferred to an NMR tube fitted with a screw-cap top. The NMR tube was sealed with a rubber-lined screw cap, through which was inserted a coaxial inner glass insert, thus creating an air-tight seal between the outer wall of the glass insert and the rubber lining of the screw cap. The connection at the NMR tube / screw cap / insert junction was further secured with parafilm and the setup was removed from the glovebox. The coaxial inner cell was fitted with a fiber optic cable featuring a sandblasted tip. The opposite end of the fiber optic cable was connected to a high-power LED light source and the sample placed in the NMR instrument. The sample was actively heated to 35 °C, after which the instrument was locked to the deuterated solvent signal, tuned, and shimmed. Irradiation of the sample (Ultra-High Power LED light, 630 nm, 100% intensity) and data collection were commenced simultaneously.

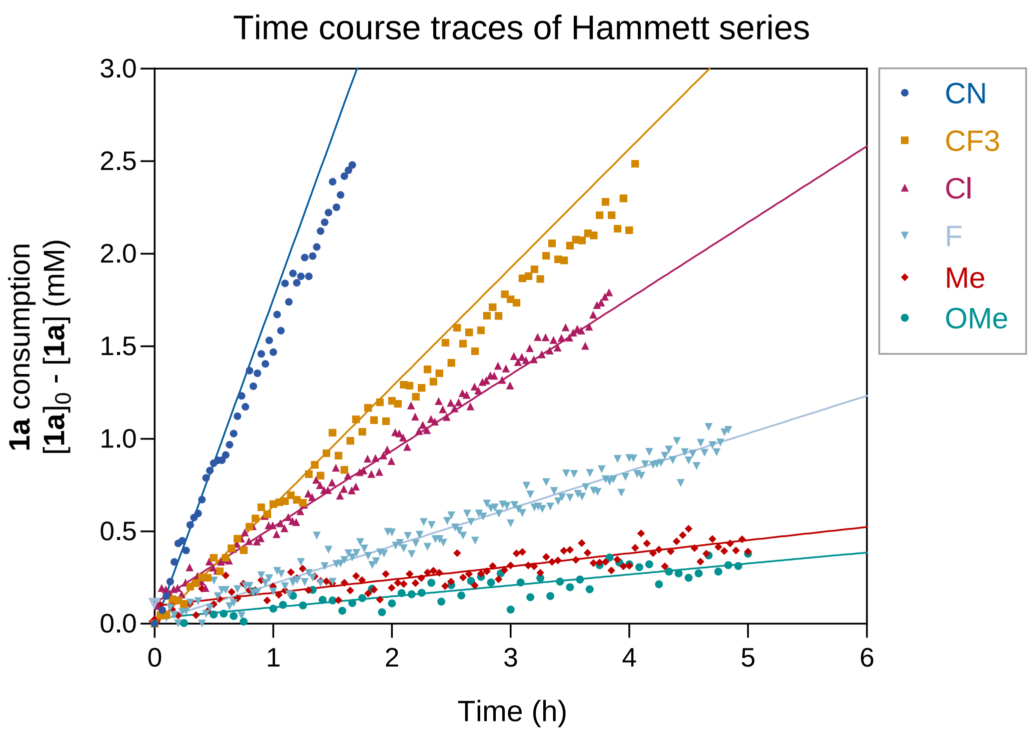

Figure S13: Time courses for red light-promoted reaction between **1a** and *para*-substituted aryl iodides used to construct Hammett series. Lines of best fit shows are constructed using data points in the initial rate regime (≤10% conversion). Standard conditions: [**1a**] = 8.3 mM, [aryl iodide] = 33.2 mM, light intensity = 100% MeCN-d_3, 35 °C. Lines of best fit were calculated by linear regression of the **1a** consumption trace at ≤ 10% conversion.

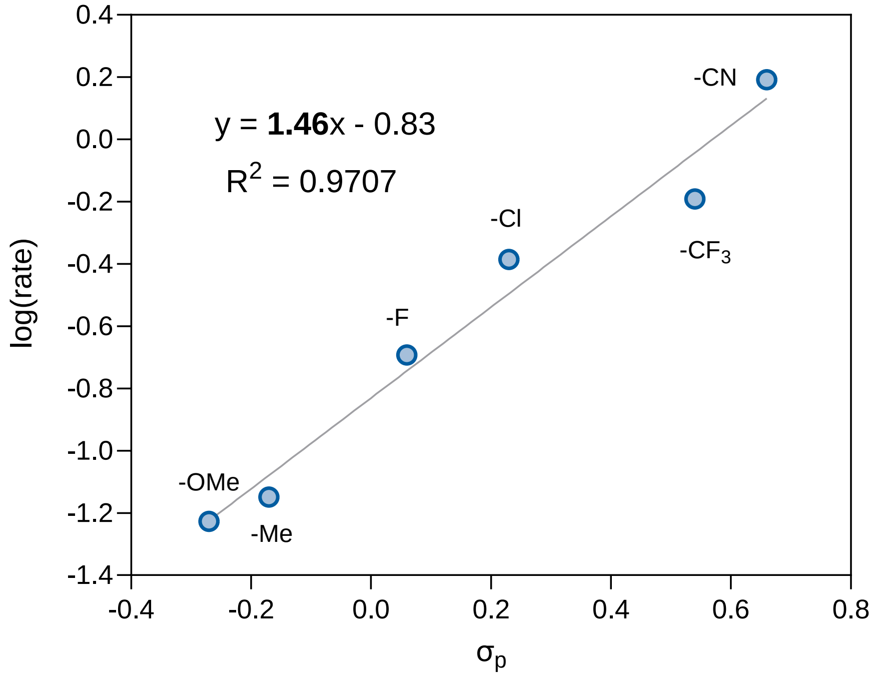

Figure S14: Hammett plot constructed using σ_p values. Reaction conditions: [**1a**] = 8.3 mM, aryl iodide = 33.2 mM, light intensity = 100% MeCN-d_3, 35 °C.

6. Radical clock and competition experiments

6.1. Intramolecular competition experiment with 2,5-diiodo-1,3-dimethylbenzene (6)

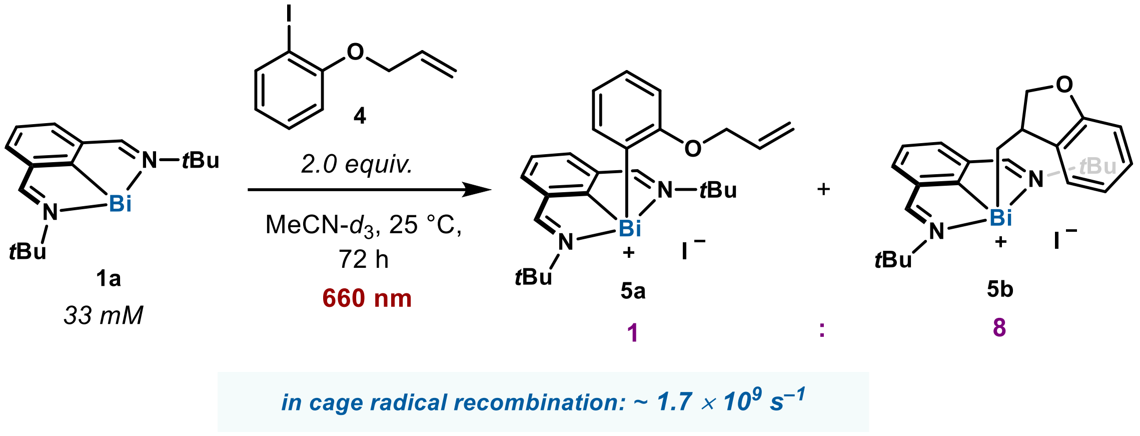

Procedure: While working in an argon-filled glovebox, a 4 mL scintillation vial was charged with **1a** (9.0 mg, 0.02 mmol, 1.0 equiv.), 1-(allyloxy)-2-iodobenzene **4** (10.4 mg, 0.04 mmol, 2.0 equiv.), and anhydrous, degassed MeCN-d_3 (0.6 mL). The vial was swirled to homogenize the reaction mixture, after which the entire solution was transferred to an NMR tube which was capped. The cap was further secured with parafilm before porting the NMR tube out of the glovebox. The solution was subjected to red light irradiation using the setup outlined in section 2 (2 × 660 nm LED PR160L lamps at 100% intensity, purchased from Kessil), keeping the temperature at around 35 °C with a cooling fan. After 72 h the mixture had changed color from dark green to yellow, indicating complete consumption of **1a**. The crude reaction was characterized by NMR spectroscopy, revealing a **5a**/**5b**/4 ratio of 0.12 : 1 : 1.2.

^{1}H NMR (5a): ^{1}H NMR (600 MHz, MeCN-d_3) δ 9.71 (s, 2H), 8.30 (d, J = 7.6 Hz, 2H), 8.07 – 8.04 (m, 1H), 7.59 (ddd, J = 7.4, 1.6, 0.4 Hz, 1H), 7.44 – 7.39 (m, 1H), 7.39 (ddd, J = 8.3, 6.9, 1.6 Hz, 1H), 6.92 (ddd, J = 7.4, 6.9, 1.5 Hz, 1H), 6.21 (ddt, J = 17.3, 10.6, 5.3 Hz, 1H), 5.54 (dq, J = 17.2, 1.6 Hz, 1H), 5.39 (dq, J = 10.6, 1.6 Hz, 1H), 4.74 (dt, J = 5.3, 1.5 Hz, 2H), 1.27 (s, 18H).

^{1}H NMR (5b): ^{1}H NMR (600 MHz, MeCN-d_3) δ 9.86 (s, 1H), 9.69 (s, 1H), 8.28 (dd, J = 7.6, 1.0 Hz, 1H), 8.27 (dd, J = 7.6, 1.0 Hz, 1H), 8.00 (t, J = 7.6 Hz, 1H), 7.08 (dtd, J = 7.5, 1.4, 0.5 Hz, 1H), 7.03 (dddd, J = 8.1, 7.5, 1.4, 0.7 Hz, 1H), 6.83 (td, J = 7.5, 1.0 Hz, 1H), 6.65 (ddt, J = 8.0, 1.0, 0.5 Hz, 1H), 4.26 – 4.18 (m, 1H), 3.34 – 3.28 (m, 2H), 2.37 – 2.30 (m, 2H), 1.56 (s, 9H), 1.46 (s, 9H).

^{1}H NMR (4): ^{1}H NMR (600 MHz, MeCN-d_3) δ 7.79 (dd, J = 7.8, 1.6 Hz, 1H), 7.34 (ddd, J = 8.3, 7.4, 1.6 Hz, 1H), 6.93 (ddq, J = 8.3, 1.4, 0.4 Hz, 1H), 6.74 (ddd, J = 7.8, 1.4 Hz, 1H), 6.08 (ddt, J = 17.3, 10.6, 4.9 Hz, 1H), 5.50 (dq, J = 17.3, 1.8 Hz, 1H), 5.29 (dq, J = 10.7, 1.6 Hz, 1H), 4.61 (dddd, J = 4.9, 1.9, 0.4 Hz, 2H).

Full spectral assignments for **5a**:

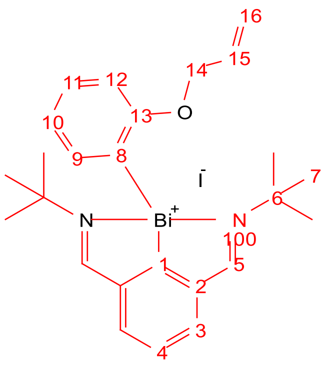

Atom	δ (ppm)	J	COSY	HSQC	HMBC	NOESY
1 C	186.643				3, 5	
2 C	149.719				4, 5	
3 C	137.248			3	3, 5	
H	8.305		4	3	1, 3	
4 C	131.501			4		
H	8.052		3	4	2	
5 C	168.268			5		
H	9.706			5	1, 2, 3, 6, 100	7
6 C	62.023				5, 7	
7 C	30.843			7	7	
H3	1.273			7	6, 7, 100	5, 14
8 C	165.336				10, 12	
9 C	138.008			9	11	
H	7.585	0.40(12), 1.60(11), 7.40(10)	10	9	11, 13	
10 C	126.760			10	12	
H	6.917	1.50(12), 6.90(11), 7.40(9)	9, 11	10	8, 12	
11 C	132.622			11	9	
H	7.387	1.60(9), 6.90(10)	10	11	9, 13	
12 C	113.160			12	10	
H	7.415	0.40(9), 1.50(10)		12	8, 10	14
13 C	161.641				9, 11, 14	
14 C	70.193			14		
H2	4.735	5.30(15), 1.40(16cis), 1.60(16trans)	15	14	13, 15, 16	7, 12
15 C	134.193			15	14	
H	6.214	5.30(14), 10.60(16cis), 17.30(16trans)	14, 16cis, 16trans	15		
16 C	118.593			16cis, 16trans	14	
Hcis	5.539	1.40(14), 10.60(15), 1.60(16trans)	15	16		
Htrans	5.386	1.60(14), 17.30(15), 1.60(16cis)	15	16		
100 N	-70.095				5, 7	

Remarks: Both sides of the ligand scaffold are chemically equivalent. The connectivity of the arene is supported by NOEs (e.g. H14-H7). The signals of C8 and C1 are significantly broadened.

Full spectral assignments for **5b**:

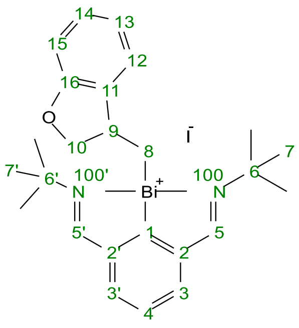

Atom	δ (ppm)	J	COSY	HSQC	HMBC	NOESY
1 C	186.262				3, 3', 5, 5', 8	
2 C	149.143				4, 5	
2' C	149.701				4, 5'	
3 C	137.361			3	3', 5	
H	8.274	7.60(4), 1.00(3')	4	3	1, 3', 5	5
3' C	137.593			3'	3, 5'	
H	8.279	7.60(4), 1.00(3)	4	3'	1, 3, 5'	5'
4 C	131.130			4		
H	7.996	7.60(3), 7.60(3')	3, 3'	4	2, 2'	
5 C	169.767			5	3	
H	9.861			5	1, 2, 3, 6, 100	3, 7, 9
5' C	169.586			5'	3'	
H	9.685			5'	1, 2', 3', 6', 100'	3', 7', 10
6 C	62.423				5, 7	
6' C	62.451				5', 7'	
7 C	31.206			7	7	
H3	1.560			7	6, 7, 100	5, 8, 9, 12
7' C	31.155			7'	7'	
H3	1.459			7'	6', 7', 100'	5', 8

Atom	δ (ppm)	J	COSY	HSQC	HMBC	NOESY
8 C	59.089			8	9, 10	
H2	2.337		9	8	1, 9, 10, 11	7, 7', 9, 10, 12
9 C	42.118			9	8, 10, 12	
H	4.219	0.70(14), 1.00(12)	8, 10	9	8, 11, 16	5, 7, 8, 10, 12
10 C	78.783			10	8	
H2	3.305		9	10	8, 9, 11, 16	5', 8, 9
11 C	138.791				8, 9, 10, 13, 15	
12 C	124.761			12	14	
H	7.076	0.50(15), 1.40(14), 7.50(13), 1.00(9)	13	12	9, 14, 16	7, 8, 9
13 C	121.582			13	15	
H	6.827	7.50(14), 1.00(15), 7.50(12)	12	13	11, 15	
14 C	129.539			14	12	
H	7.033	0.70(9), 7.50(13), 8.00(15), 1.40(12)	14, 15	14	12, 16	
15 C	110.391			15	13	
H	6.647	8.00(14), 1.00(13), 0.50(12), 0.50(?)	14	15	11, 13, 16	
16 C	160.319				9, 10, 12, 14, 15	
100 N	-74.010				5, 7	
100' N	-73.276				5', 7'	

Remarks: The two sides of the ligand scaffold are chemically different, likely due to the chiral center (C9) and a "formal" hindered rotation around the C1-Bi bond (multiple mechanisms are imaginable here). The connectivity of the dihydrobenzofuran moiety and the *N*,*C*,*N*-Bi(III) pincer part is supported by multiple NOEs (e.g. H9-H7) and a HMBC cross peak from H8 to C1. The signals of C1 and C8 are broadened due to a residual quadrupolar interaction with ^{209}Bi.

Full spectral characterization of **4**:

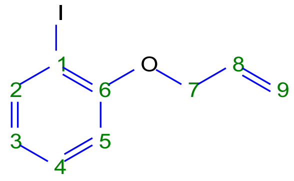

Atom	δ (ppm)	J	COSY	HSQC	HMBC	NOESY
1 C	86.728				3	
2 C	140.365			2	4	
H	7.789	1.60(4), 7.80(3)	3	2	4, 6	
3 C	123.705			3		
H	6.743	7.80(2), 7.40(4), 1.40(5)	2, 4	3	1, 5	
4 C	130.722			4	2	
H	7.340	1.60(2), 8.30(5), 7.40(3)	3, 5	4	2, 6	
5 C	113.795			5	3	
H	6.931	8.30(4), 1.40(3), 0.40(7)	4	5		7
6 C	158.047				2, 4, 7	
7 C	70.351			7	8, 9cis, 9trans	
H2	4.606	0.40(5), 4.90(8), 1.60(9cis), 1.90(9trans)	8, 9cis, 9trans	7	6, 8, 9	5, 9trans
8 C	134.069			8	7	
H	6.075	10.60(9trans), 17.30(9cis), 4.90(7)	7, 9cis, 9trans	8	7	
9 C	117.829			9cis, 9trans	7	
Hcis	5.290	17.30(8), 1.80(9trans), 1.60(7)	7, 8, 9trans	9	7	
Htrans	5.497	10.60(8), 1.80(9cis), 1.90(7)	7, 8, 9cis	9	7	7

^{1}H NMR spectrum

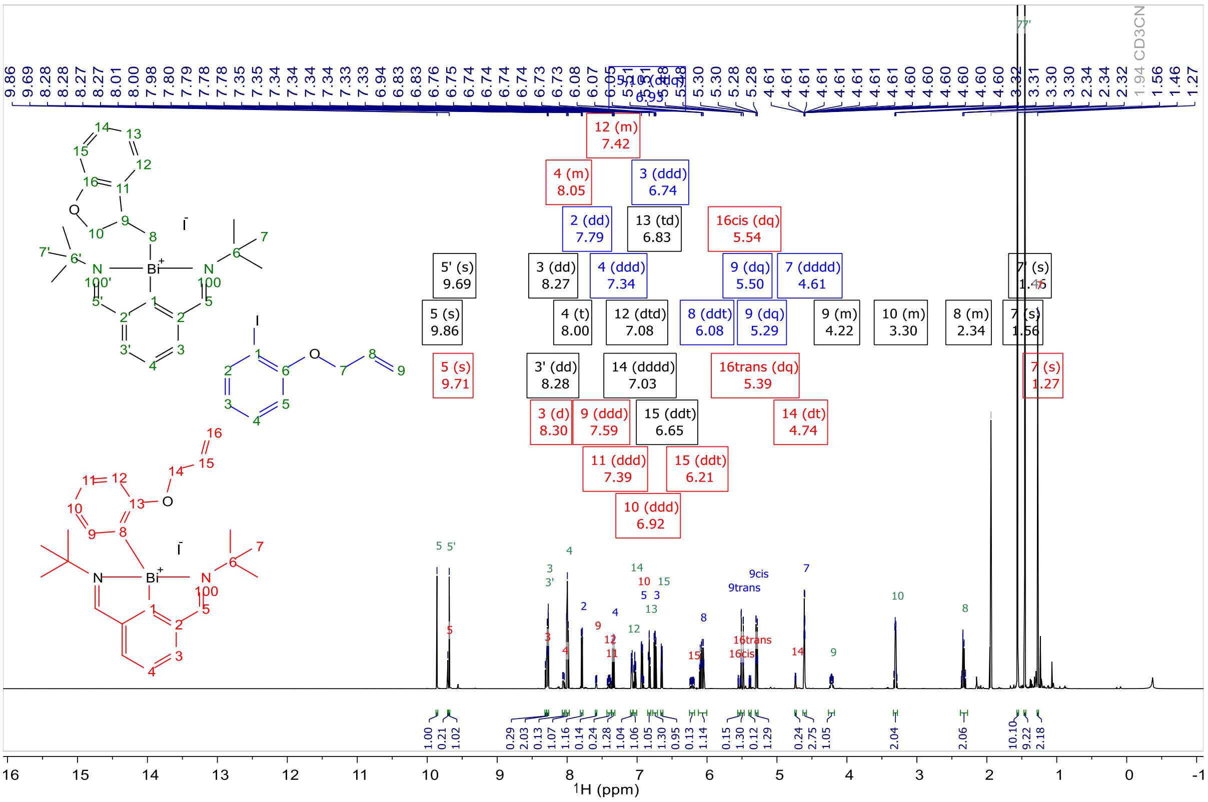

$^{13}C\{^{1}H\}$ NMR

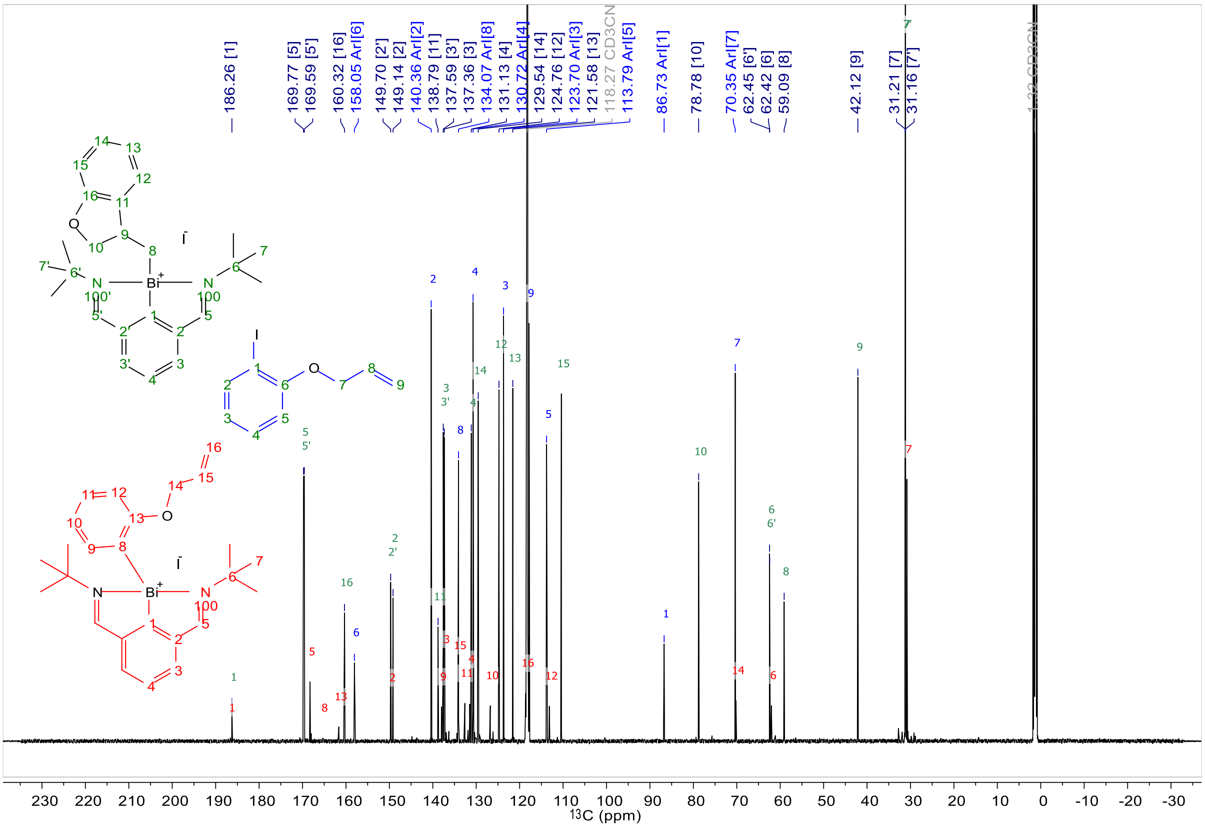

^{1}H-^{13}C edited HSQC

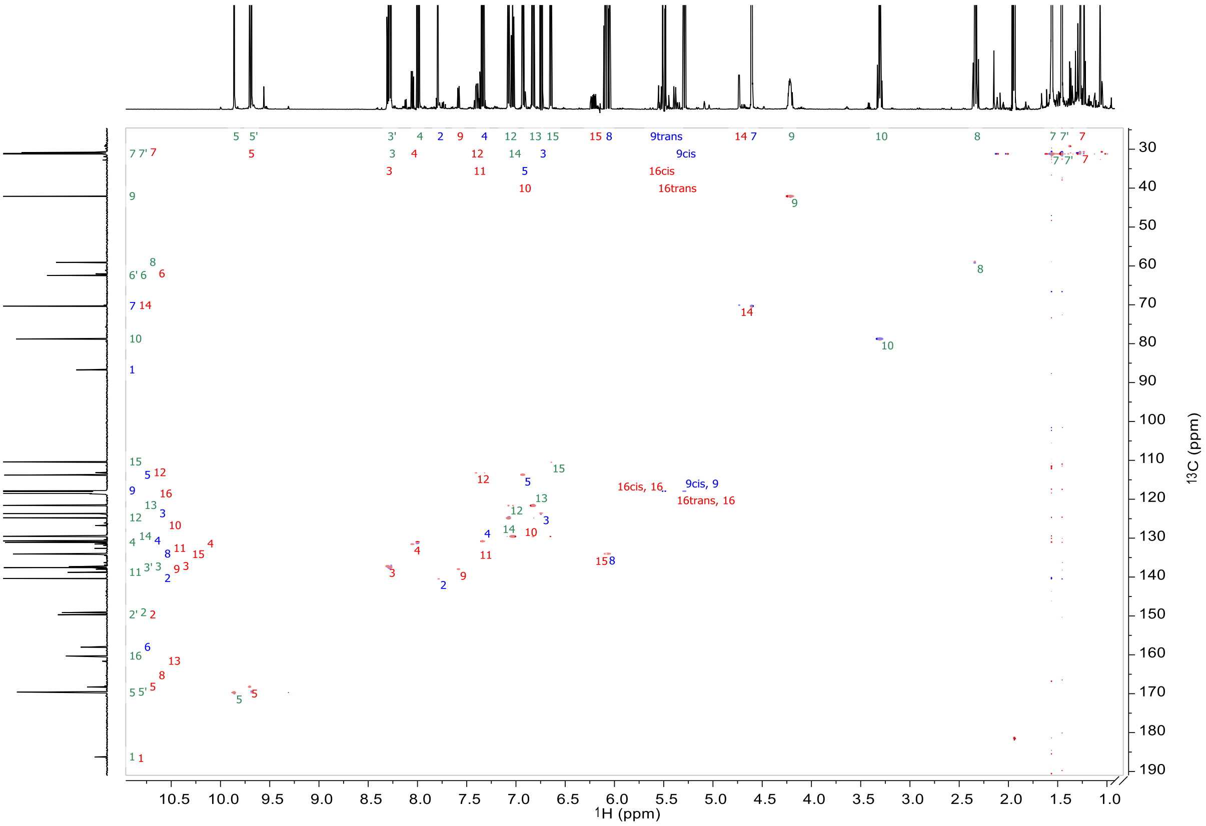

^{1}H-^{13}C HMBC

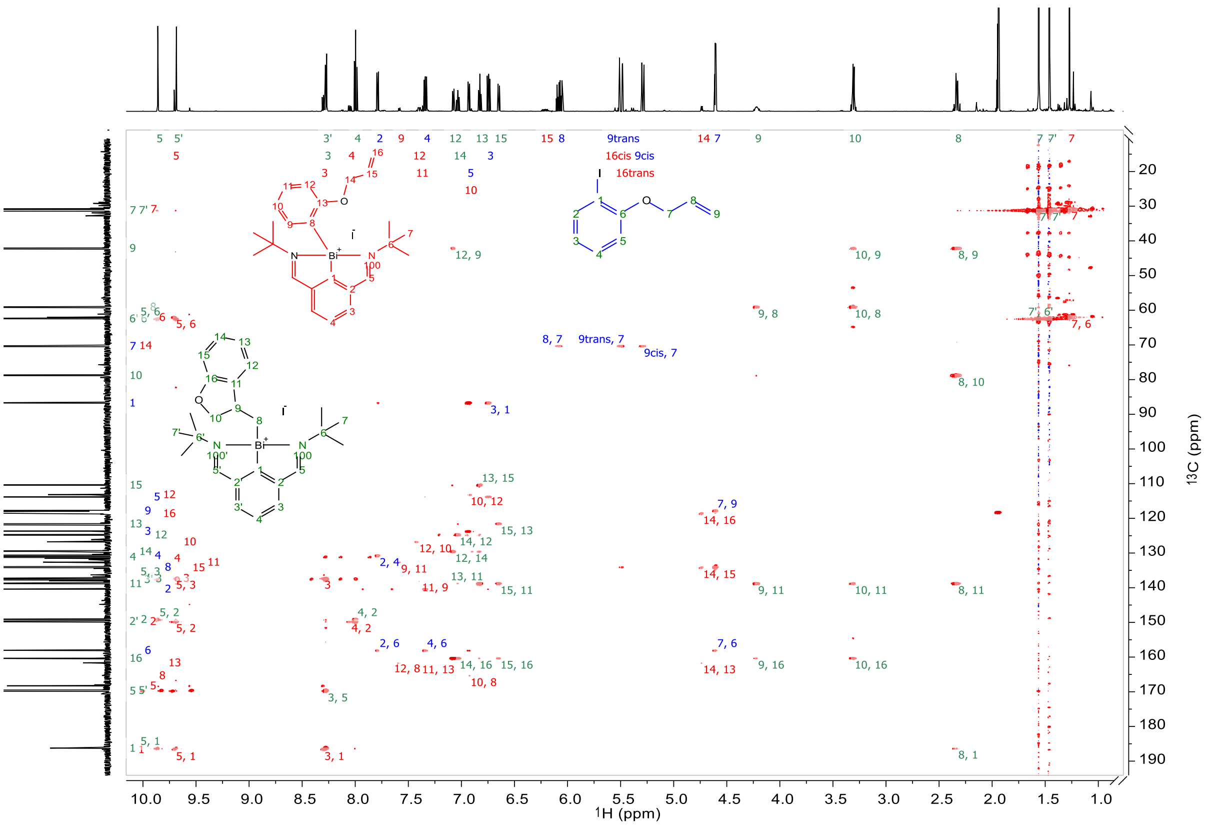

^{1}H-^{1}H COSY

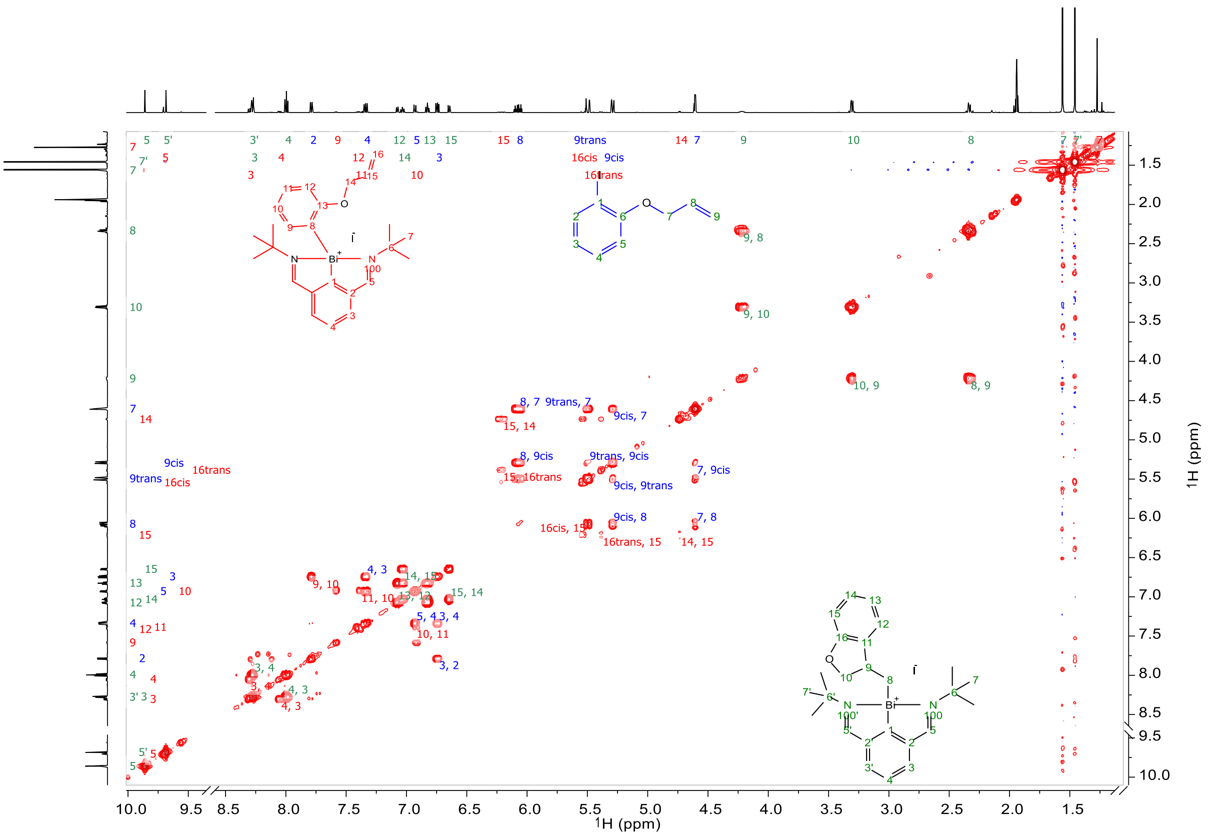

^{1}H-^{1}H NOESY

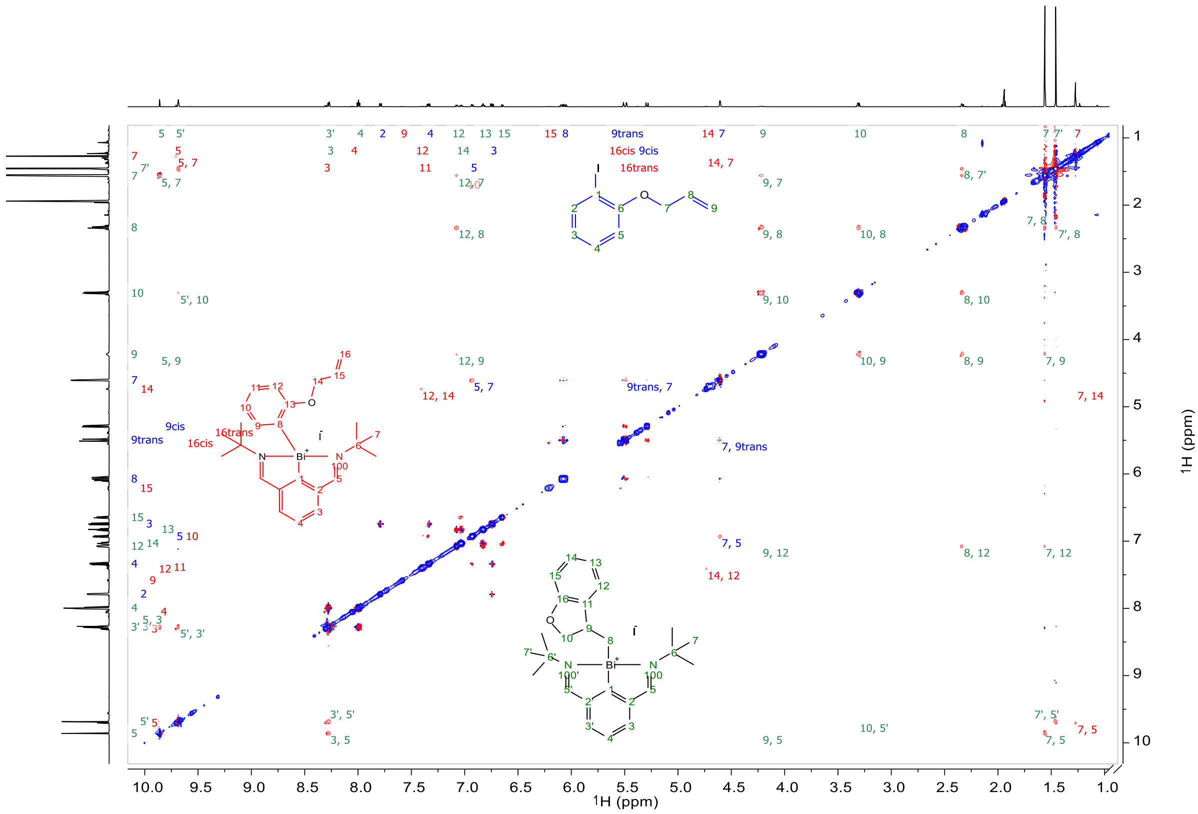

^{1}H-^{15}N HMBC

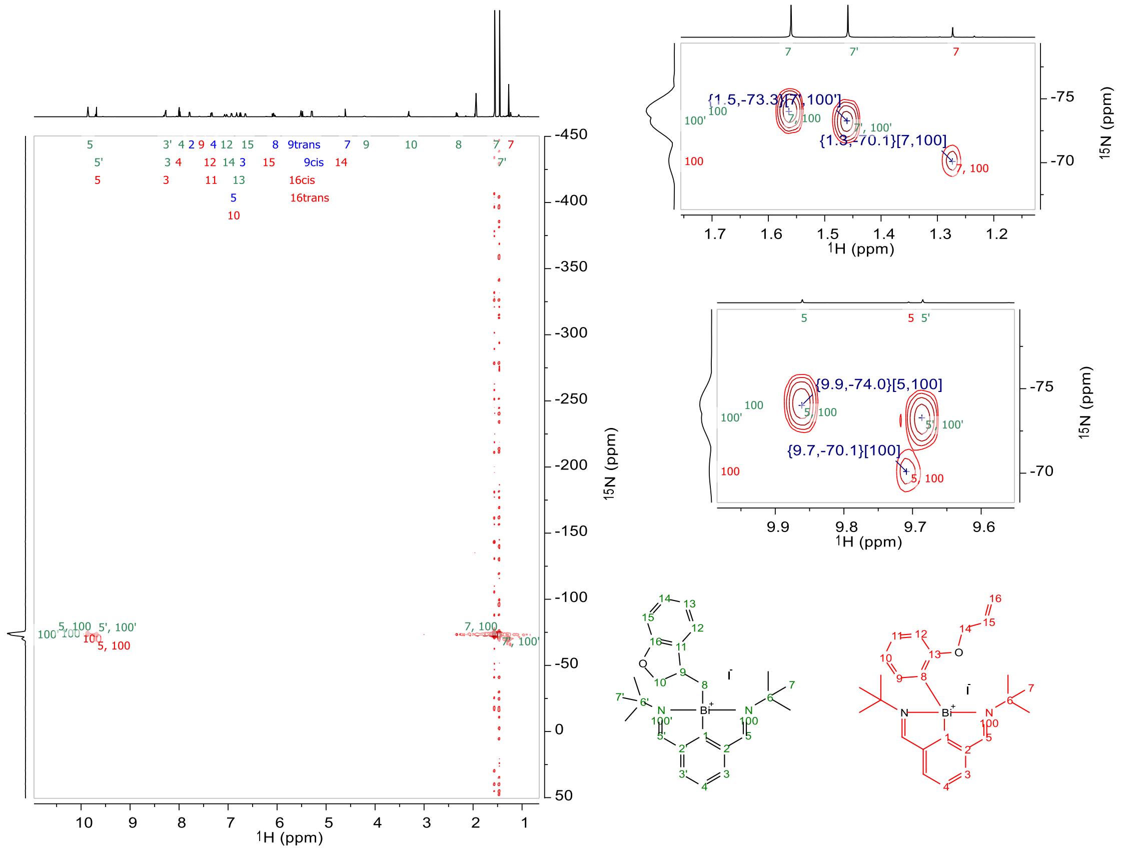

6.2. Intramolecular competition experiment with 2,5-diiodo-1,3-dimethylbenzene (6)

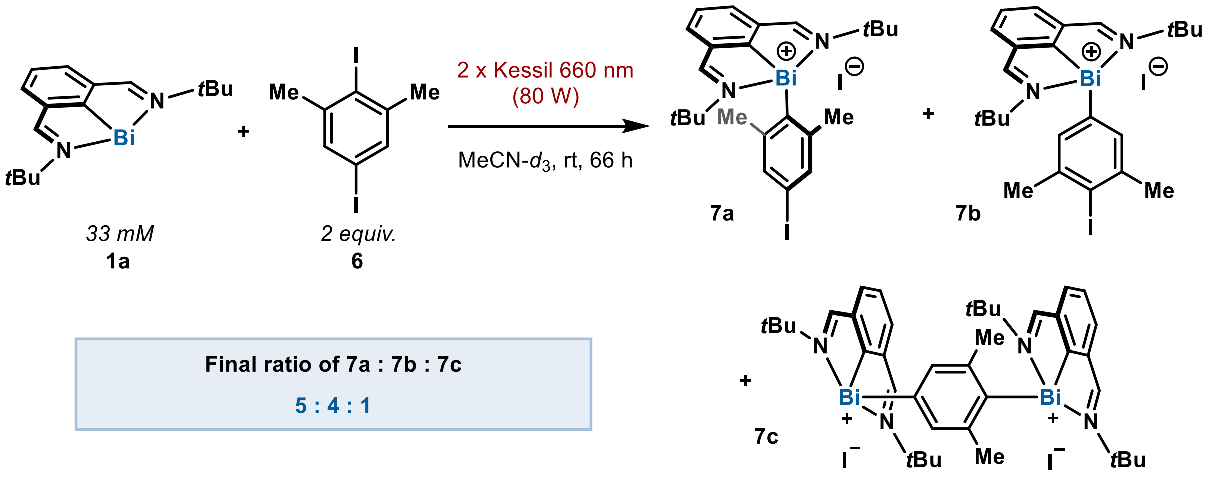

Procedure: While working in an argon-filled glovebox, a 4 mL scintillation vial was charged with **1a** (9.0 mg, 0.020 mmol, 1.0 equiv.), diiodoarene **6** (14.2 mg, 0.040 mmol, 2.0 equiv.), and anhydrous, degassed MeCN-d_3 (0.6 mL). The vial was swirled to homogenize the reaction mixture, after which the entire solution was transferred to an NMR tube which was capped. The cap was further secured with parafilm before transferring the NMR tube out of the glovebox. The solution was subjected to red light irradiation using the setup outlined in section 2 (2 × 660 nm LED PR160L lamps at 100% intensity, purchased from Kessil), while keeping the temperature at around 35 ºC with the aid of a cooling fan. After 66 h the mixture had changed color from dark green to yellow, indicating complete consumption of **1a**. The reaction tube was placed directly in the NMR instrument and the ratio of products was analyzed by ^{1}H NMR. This revealed a **7a**/**7b**/**7c** ratio of 5 : 4 : 1.

^{1}H NMR (7a): ^{1}H NMR (300 MHz, MeCN-d_3) δ 9.79 (s, 2H), 8.33 (d, J = 7.57 Hz, 2H), 8.03 (dd, J = 7.47 Hz, 1H), 7.86 (d, J = 1.37, 1H), 7.76 (d, J = 1.47, 1H), 2.89 (s, 3H), 1.42 (s, 3H), 1.28 (s, 18H).

^{1}H NMR (7b): ^{1}H NMR (300 MHz, MeCN-d_3) δ 9.73 (s, 2H), 8.33 (d, J = 7.59 Hz, 2H), 8.06 (dd, J = 7.44, 1H), 7.80 (h, J = 0.65, 2H), 2.30 (t, 0.59, 6H), 1.31 (s, 18H).

^{1}H NMR (7c): ^{1}H NMR (300 MHz, MeCN-d_3) δ 9.76 (s, 2H), 9.74 (s, 2H), 8.32 (d, J = 7.56 Hz, 2H), 8.31 (d, J = 7.58, 2H), 8.24 (brs, 1H), 8.18 (brs, 1H), 8.04 (dd, J = 7.41 Hz, 1H), 8.00 (dd, J = 7.39 Hz, 1H), 2.80 (brs, 3H), 1.33 (brs, 3H), 1.25 (s, 18H), 1.19 (s, 18H).

HRMS (ESI Positive) calculated for **7a** and **7b**: $C_{26}H_{30}BiN_2$ $[M\text{-}I]^+$: 683.13; found: 683.13.

HRMS (ESI Positive) calculated for **7c**: $C_{40}H_{54}Bi_2IN_4$ $[M\text{-}I]^+$: 1135.30; found: 1135.30.

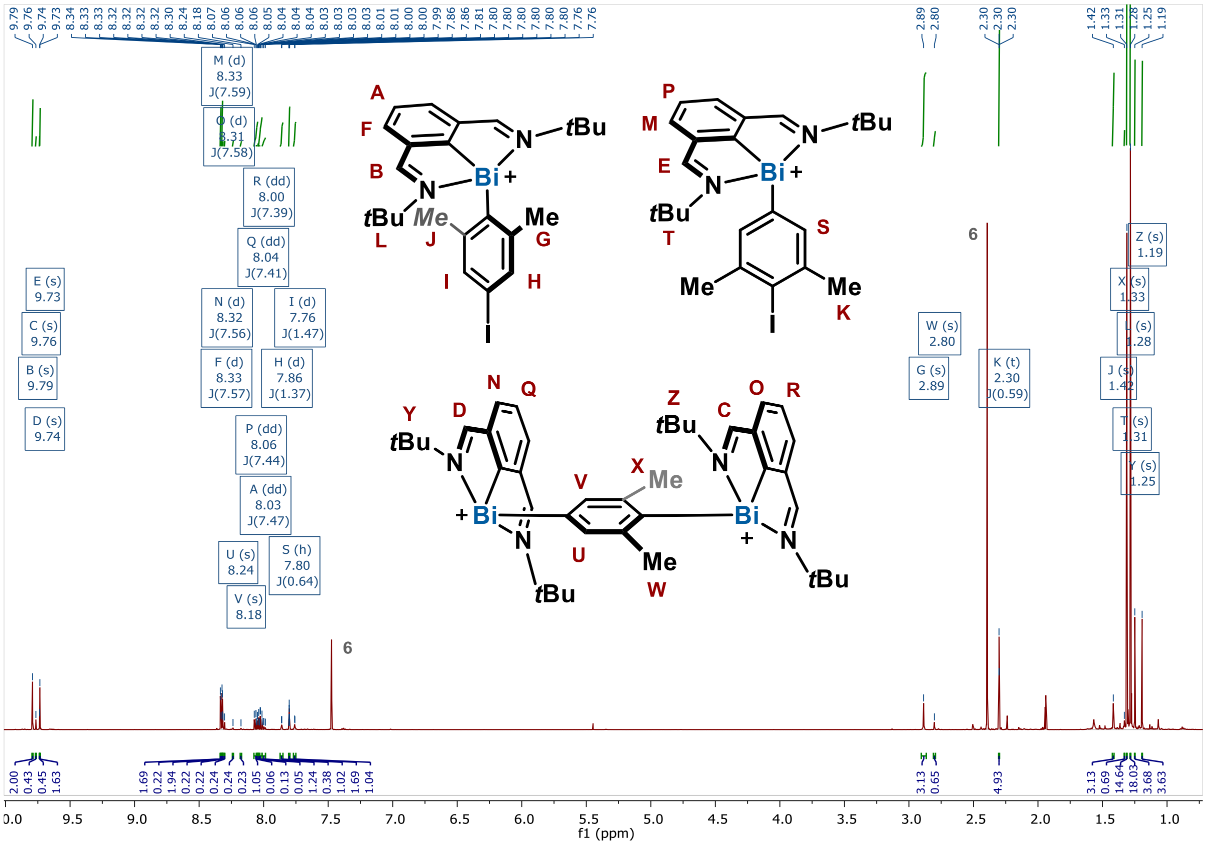

Figure S15: Full ^{1}H NMR spectrum of crude reaction mixture featuring a 5 : 4 : 1 mixture of **7a**, **7b**, and **7c**. Assigned structures of products included.

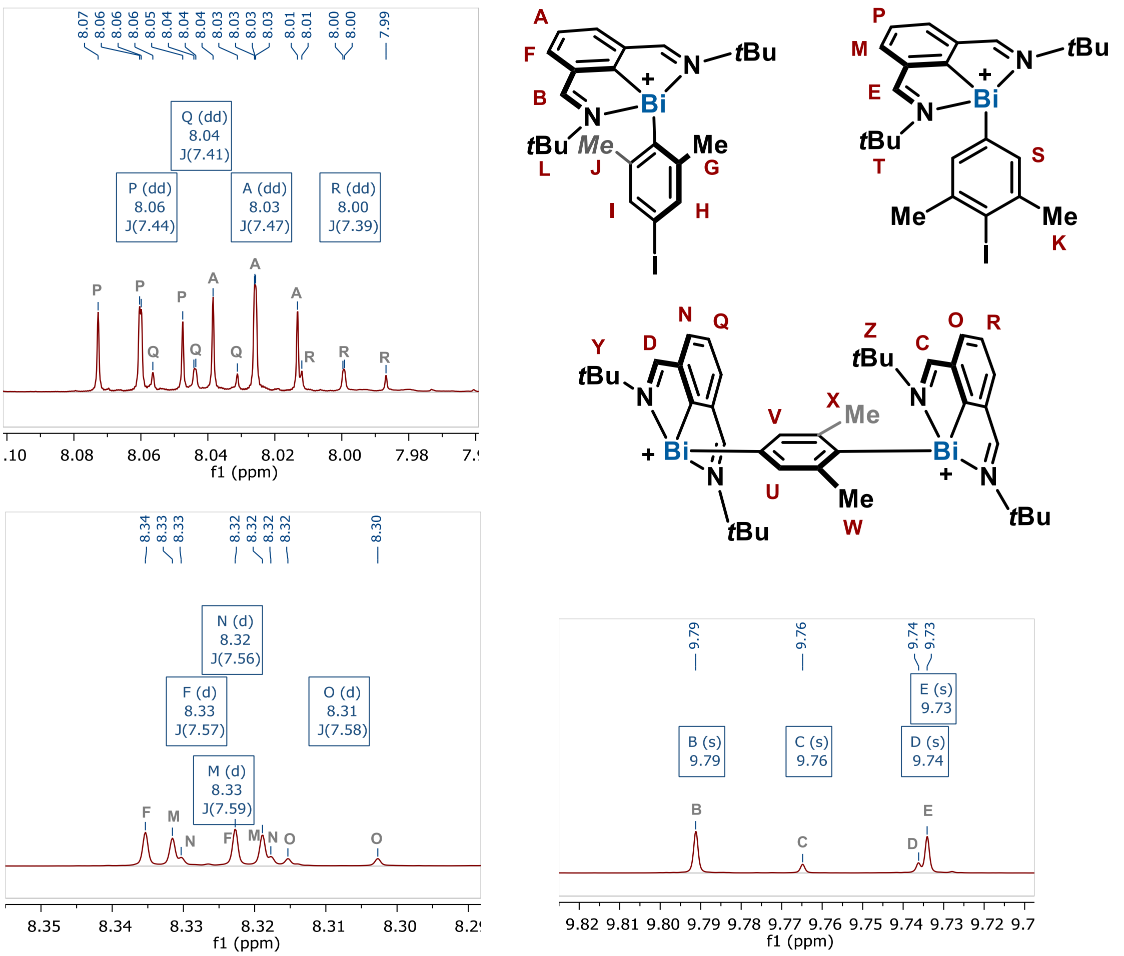

Figure S16: Enlarged portions of the crude ^{1}H NMR spectrum to visually resolve overlapping peaks from intramolecular competition reaction with 2,5-diiodo-1,3-dimethylbenzene **6**. Assigned structures of products included, and multiplet peaks labeled.

The above (crude) spectra reveal a 5 : 4 : 1 ratio of **7a**, **7b**, and **7c**. Protons for the *N,C,N* ligand backbone for all species reveal a plane of symmetry (due to homotopic nature of aldimine protons and protons *meta* to Bi center). However, protons on axial arene are desymmetrized and broadened in **7a**, indicative of hindered rotation about the Bi–C bond axis.

6.3. Intramolecular competition experiment with 2,5-diiodo-1,3-diisopropylbenzene (**8**)

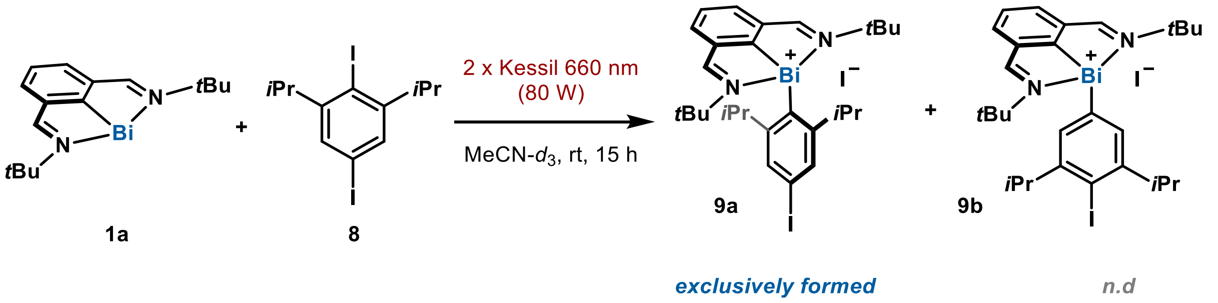

Procedure: While working in an argon-filled glovebox, a 4 mL scintillation vial was charged with **1a** (9.0 mg, 0.020 mmol, 1.0 equiv.), diiodoarene **8** (16.6 mg, 0.040 mmol, 2.0 equiv.), and anhydrous, degassed MeCN-d_3 (0.6 mL). The vial was swirled to homogenize the reaction mixture, after which the entire solution was transferred to an NMR tube which was capped. The cap was further secured with parafilm before transferring the NMR tube out of the glovebox. The solution was subjected to red light irradiation using the setup outlined in section 2 (2 × 660 nm LED PR160L lamps at 100% intensity, purchased from Kessil), while keeping the temperature at around 35 ºC with the aid of a cooling fan. After 15 h the mixture had changed color from dark green to yellow, indicating complete consumption of **1a**. The reaction tube was placed directly in the NMR instrument and the ratio of products was analyzed by quantitative ^{1}H NMR. This revealed exclusive formation of **9a**.

^{1}H NMR (9a): ^{1}H NMR (300 MHz, MeCN-d_3) δ 9.78 (s, 2H), 8.39 (d, J = 7.6 Hz, 2H), 8.08 (d, J = 1.9 Hz, 1H), 8.08 – 8.02 (m, 1H), 7.84 (d, J = 1.9 Hz, 1H), 3.86 (p, J = 6.6 Hz, 1H), 1.95 (q, J = 7.0 Hz, 1H), 1.53 (d, J = 6.6 Hz, 6H), 1.31 (s, 18H), 0.53 (d, J = 6.7 Hz, 6H).

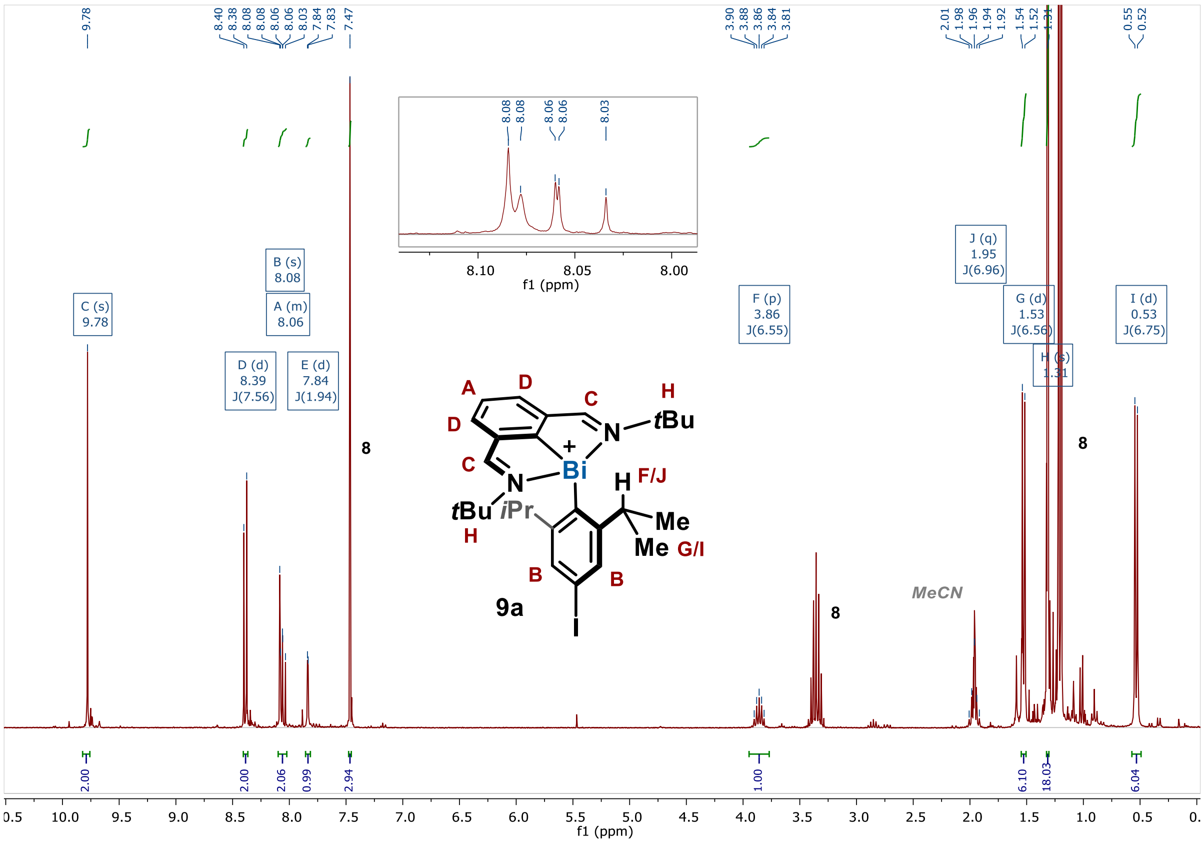

Figure S17: Full ^{1}H NMR spectrum of crude reaction mixture from intramolecular competition with 2,5-diiodo-1,3-diisopropylbenzene, featuring exclusive formation of **9a**.

The above (crude) spectrum reveals exclusive formation of product **9a** with excess **8** (peaks labeled accordingly). Protons for the *N,C,N* ligand backbone reveal a plane of symmetry (due to homotopic nature of protons D, C, and H). However, protons on axial arene are sharp and desymmetrized, indicative of highly hindered rotation about the Bi–C bond axis (much slower than timescales of quantitative NMR experiment). Enlarged portion of spectrum included to facilitate visualization of the overlapping signals of protons **A** and **B**.

6.4. Intermolecular competition experiment between 2-iodo-1,3-diisopropylbenzene (10) and 4-iodobenzonitrile (2) & independent synthesis of 11a

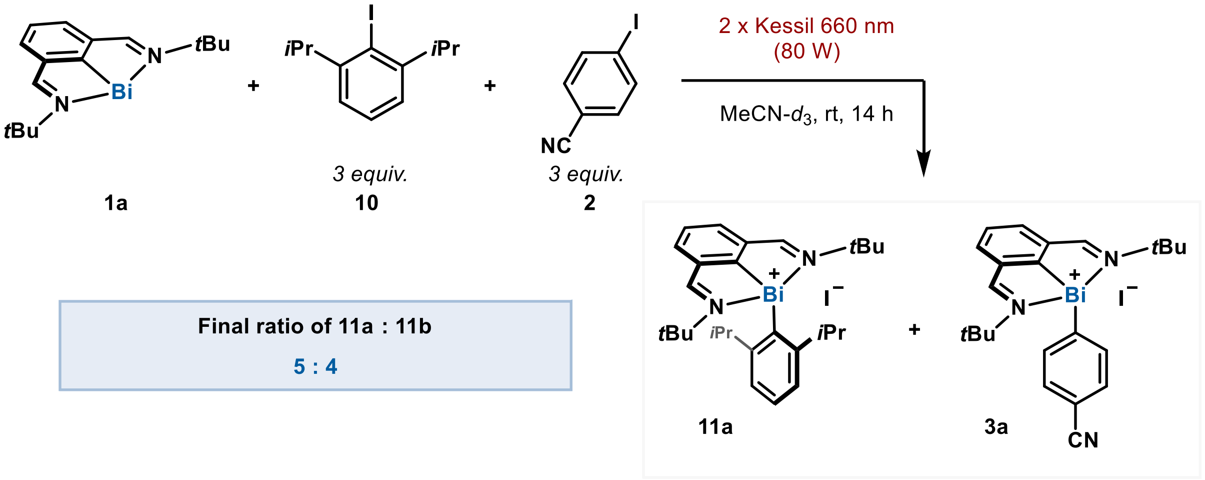

Procedure: While working in an argon-filled glovebox, a 4 mL scintillation vial was charged with **1a** (9.0 mg, 0.02 mmol, 1 equiv.), 2-iodo-1,3-diisopropylbenzene **10** (17.3 mg, 0.06 mmol, 3 equiv.), 4-iodobenzonitrile **2** (13.7 mg, 0.06 mmol, 3 equiv.), and anhydrous, degassed MeCN-d_3 (0.6 mL). The vial was swirled to homogenize the reaction mixture, after which the entire solution was transferred to an NMR tube which was capped. The cap was further secured with parafilm before transferring the NMR tube out of the glovebox. The solution was subjected to red light irradiation using the setup outlined in section 2 (2 × 660 nm LED PR160L lamps at 100% intensity, purchased from Kessil), while keeping the temperature at around 35 °C with the aid of a cooling fan. After 14 h the mixture had changed color from dark green to yellow, indicating complete consumption of **1a**. The reaction tube was placed directly in the NMR instrument and the ratio of products was analyzed by quantitative ^{1}H NMR. This revealed a 5 : 4 ratio of **11a** to **3a**. Spectral assignment was aided by the independent synthesis and ^{1}H NMR characterization of **11a**. The latter compound was independently synthesized using the same procedure outlined above, with omission of **2**. The spectral data for compound **3a** has already been reported in the literature.[3] The spectral details of the two compounds and the synthesis of **11a** are reported below.

^{1}H NMR (11a): ^{1}H NMR (300 MHz, MeCN-d_3) δ 9.76 (s, 2H), 8.37 (d, J = 7.6 Hz, 2H), 8.04 (dd, J = 7.9, 7.3 Hz, 1H), 7.78 (dd, J = 7.4, 1.7 Hz, 1H), 7.54 (dd, J = 7.6, 1.8 Hz, 1H), 7.48 (t, J = 7.5 Hz, 1H), 3.94 (hept, J = 6.6 Hz, 1H), 2.01 (hept, J = 6.7 Hz, 1H), 1.53 (d, J = 6.5 Hz, 6H), 1.29 (s, 18H), 0.54 (d, J = 6.8 Hz, 6H).

^{1}H NMR (3a): ^{1}H NMR (300 MHz, MeCN-d_3) δ 9.78 (s, 2H), 8.35 (m, 2H), 8.29 (d, J = 8.3 Hz, 2H), 8.08 (dd, J = 7.9, 7.3 Hz, 1H), 7.76 (m, 2H), 1.31 (s, 18H).

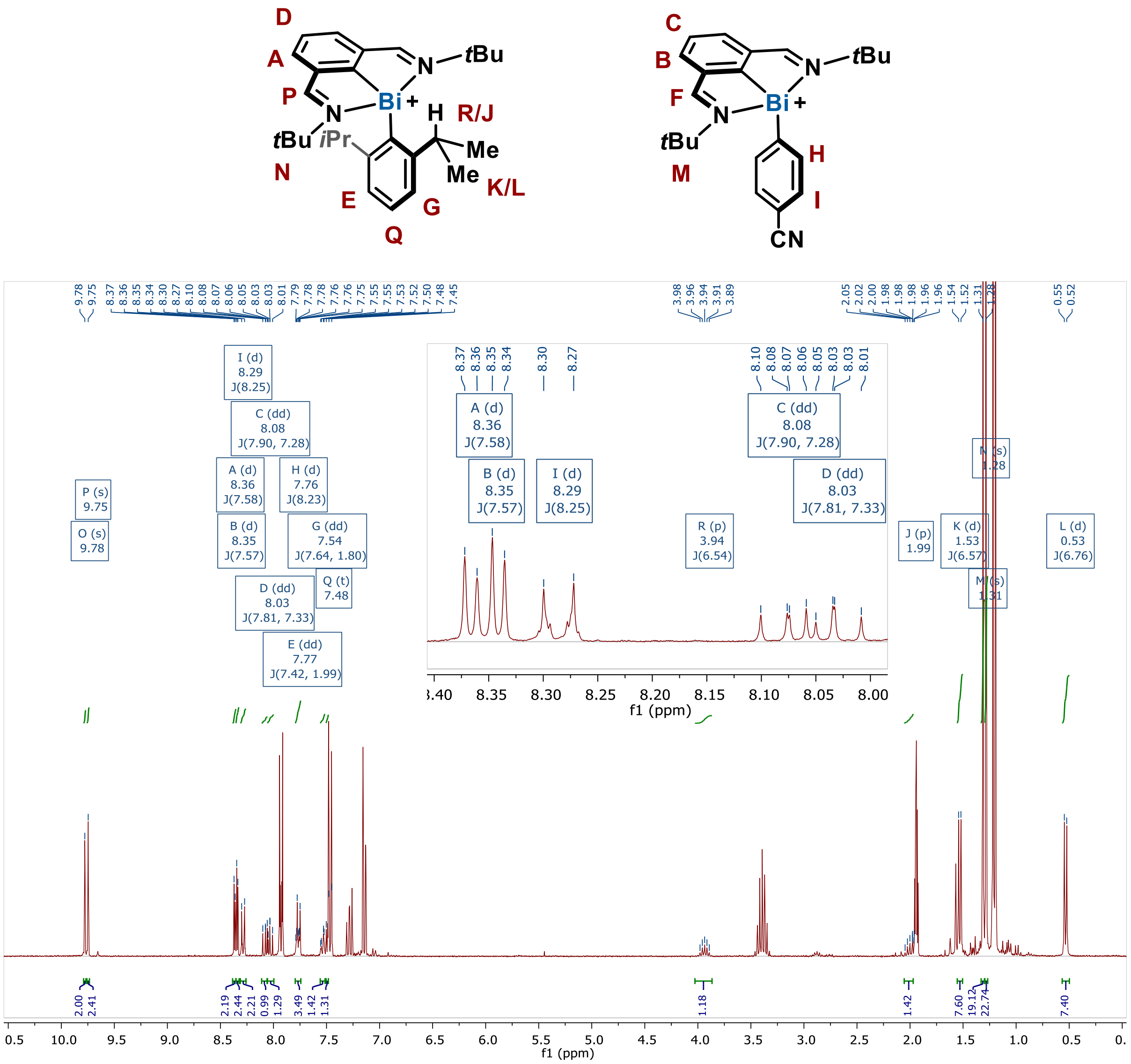

Figure S18: Crude ^{1}H NMR spectrum of competition reaction between **10** and **2**. Enlarged portion of spectrum included for clarity due to overlapping peaks in aromatic region. Structures of products included and proton signals assigned.

Independent synthesis of **11a**:

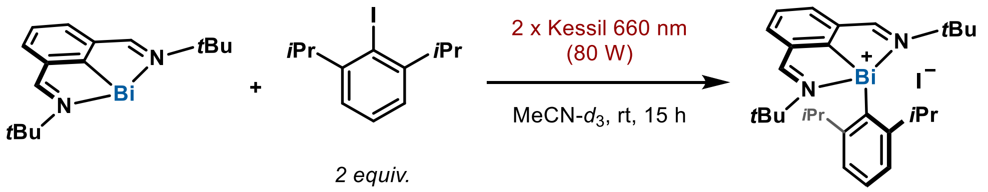

1a **10** **11a**

Procedure: While working in an argon-filled glovebox, a 4 mL scintillation vial was charged with **1a** (9.0 mg, 0.02 mmol, 1 equiv.), 2-iodo-1,3-diisopropylbenzene **10** (11.5 mg, 0.04 mmol, 2 equiv.), and anhydrous, degassed MeCN-d_3 (0.6 mL). The vial was swirled to homogenize the reaction mixture, after which the entire solution was transferred to an NMR tube which was capped. The cap was further secured with parafilm before transferring the NMR tube out of the glovebox. The solution was subjected to red light irradiation using the setup outlined in section 2 (2 × 660 nm LED PR160L lamps at 100%

intensity, purchased from Kessil), while keeping the temperature at around 35 ºC with the aid of a cooling fan. After 15 h the mixture had changed color from dark green to yellow, indicating complete consumption of **1a**. The reaction tube was placed directly in the NMR instrument and the crude reaction was analyzed by quantitative ^{1}H NMR. This revealed exclusive presence of **11a** and excess **10**.

^{1}H NMR (11a): ^{1}H NMR (300 MHz, MeCN-d_3) δ 9.76 (s, 2H), 8.37 (d, *J* = 7.6 Hz, 2H), 8.04 (dd, *J* = 7.9, 7.3 Hz, 1H), 7.78 (dd, *J* = 7.4, 1.7 Hz, 1H), 7.54 (dd, *J* = 7.6, 1.8 Hz, 1H), 7.48 (t, *J* = 7.5 Hz, 1H), 3.94 (hept, *J* = 6.6 Hz, 1H), 2.01 (hept, *J* = 6.7 Hz, 1H), 1.53 (d, *J* = 6.5 Hz, 6H), 1.29 (s, 18H), 0.54 (d, *J* = 6.8 Hz, 6H.

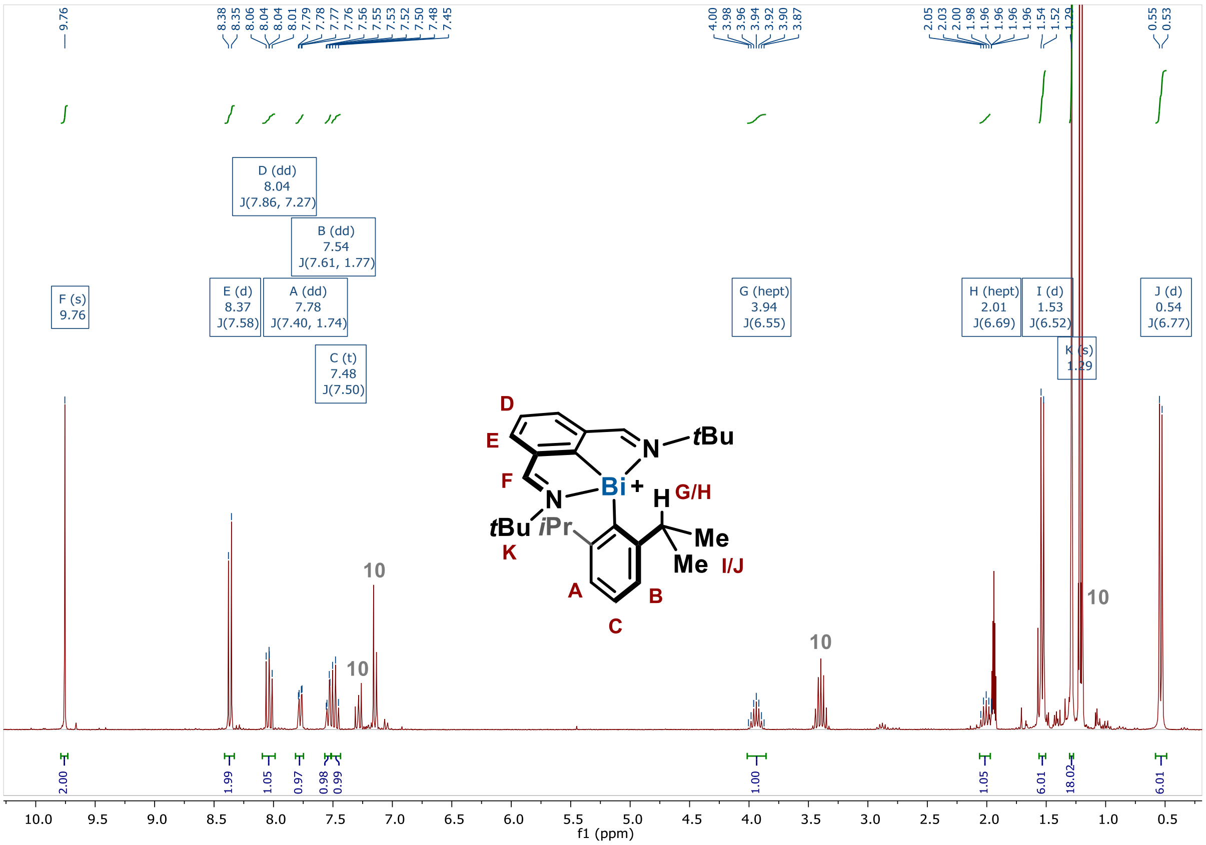

Figure S19: Crude spectrum obtained after independent synthesis of **11a**. Peaks associated with oxidative addition product are assigned and labeled on the structure. Peaks corresponding to excess **10** are labeled on the NMR spectrum.

6.5. Intermolecular competition experiment between 2-iodo-1,3-diisopropylbenzene (**10**) and 4-iodobenzonitrile (**2**)

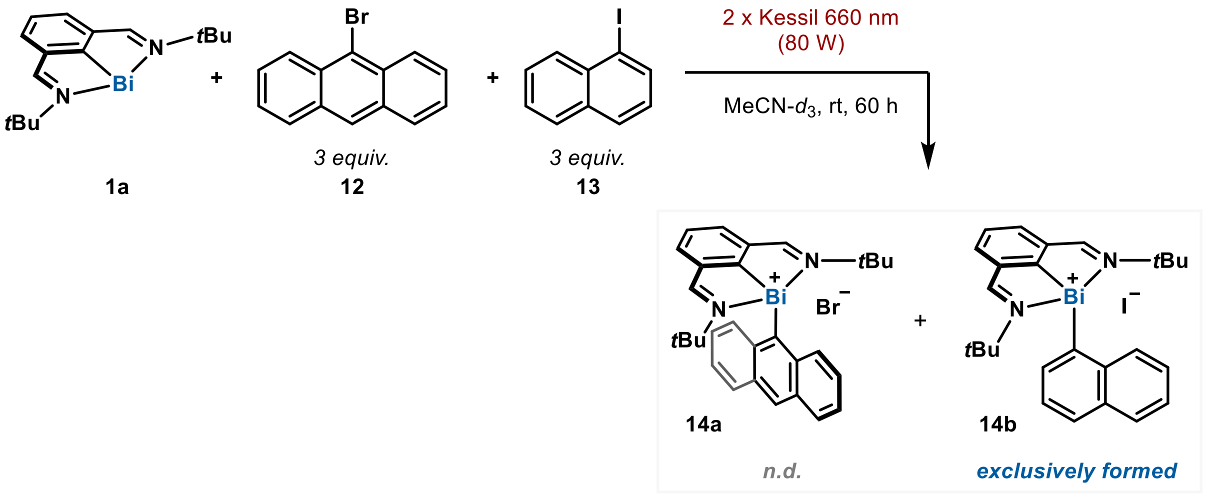

Procedure: While working in an argon-filled glovebox, a 4 mL scintillation vial was charged with **1a** (9.0 mg, 0.020 mmol, 1.0 equiv.), 9-bromoanthracene **12** (15.4 mg, 0.060 mmol, 3.0 equiv.), 1-iodonaphthalene **13** (15.2 mg, 0.060 mmol, 3.0 equiv.), and anhydrous, degassed MeCN-d_3 (0.6 mL). The vial was swirled to homogenize the reaction mixture, after which the entire solution was transferred to an NMR tube which was capped. The cap was further secured with parafilm before transferring the NMR tube out of the glovebox. The solution was subjected to red light irradiation using the setup outlined in section 2 (2 × 660 nm LED PR160L lamps at 100% intensity, purchased from Kessil), while keeping the temperature at around 35 °C with the aid of a cooling fan. After 60 h the mixture changed color from dark green to yellow, indicating complete consumption of **1a**. The contents of the reaction tube were transferred to a Schlenk flask and the solvent removed under vacuum. The residue wash washed with anhydrous, degassed, and inhibitor-free diethyl ether (3 × 5 mL) to remove most of the excess 1-iodonaphthalene and 9-bromoanthracene. The Schlenk flask was ported into the glovebox and the residue complemented with MeCN-d_3 (0.6 mL). The solution was transferred to an NMR tube and subsequently analyzed by quantitative ^{1}H NMR. This revealed exclusive formation of product **14b**, whose spectral features matched those previously reported in the literature.[3]

^{1}H NMR (14b): ^{1}H NMR (300 MHz, MeCN-d_3) δ 9.71 (s, 2H), 8.57 (d, *J* = 8.40 Hz, 1H), 8.36 (dd, *J* = 7.61, 0.42 Hz, 2H), 8.14 (m, 1H), 8.13 – 8.06 (m, 2H), 8.03 – 8.00 (m, 1H), 7.77 (ddd, *J* = 8.31, 6.86, 1.37 Hz, 1H), 7.65 – 7.59 (m, 1H), 7.42 (dd, *J* = 8.2, 7.0 Hz, 1H), 1.15 (s, 18H).

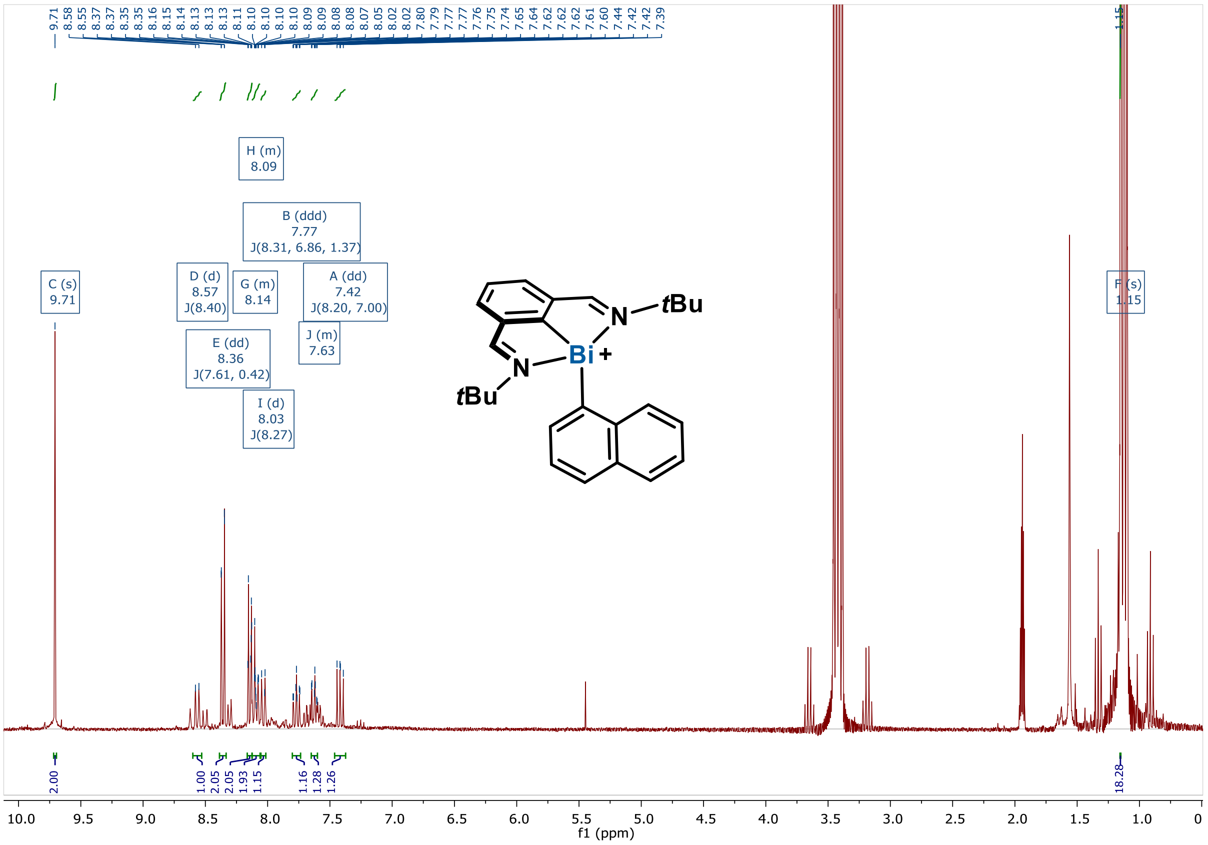

Figure S20: Crude spectrum obtained after intermolecular competition between 9-bromoanthracene **12** and 1-iodonaphthalene **13**, revealing exclusive formation of product **14b**.

7. Electrochemical data

Cyclic voltammograms were collected using a 3-electrode cell consisting of a 1.6 mm Ø glassy carbon working electrode, a platinum wire counter electrode, and a bare silver wire as a pseudoreference electrode at ambient temperature in an argon-filled glovebox equipped with electrochemical outlets. Sublimed ferrocene was added as the internal reference. All potentials in V vs $Fc^{0/+}$. The redox potential of bismuthinidene **1a** was estimated by finding the half-wave potential $E_{1/2}$, calculated by finding the arithmetic mean of the anodic and cathodic peak potentials (E_{pa} and E_{pc}, respectively). Redox potentials of aryl iodides estimated by finding the half-peak potential, $E_{p/2}$. The latter value was calculated by finding the corrected cathodic peak current density (j_{pc}') and determining the potential ($E_{p/2}$) at half this value (j_{pc}'). The corrected cathodic peak current density (j_{pc}') was obtained by correcting the cathodic peak current density (j_{pc}) for the background current. For voltammograms with two cathodic reduction features (observed for diiodoarenes), the more anodic peak is used to calculate the $E_{p/2}$.

Cyclic voltammograms of **1a** and aryl iodides used in intermolecular competition studies are shown below.

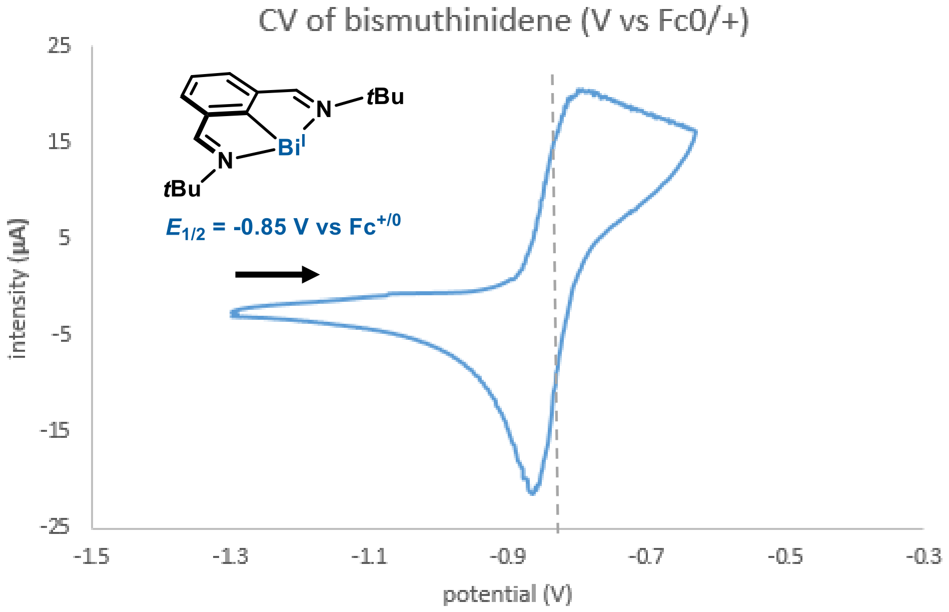

Figure S21. Cyclic voltammogram of a 1 mM solution of **1a** in CH_3CN using 0.1 M [$^{n}Bu_4N$][PF_6] as the supporting electrolyte at ambient temperature; scan rate: 100 mV/s. Potential in V vs $Fc^{0/+}$. Black arrow indicates the direction of the potential sweep. Dashed gray line represents the half-wave potential, $E_{1/2} = -0.85$ V vs $Fc^{+/0}$).

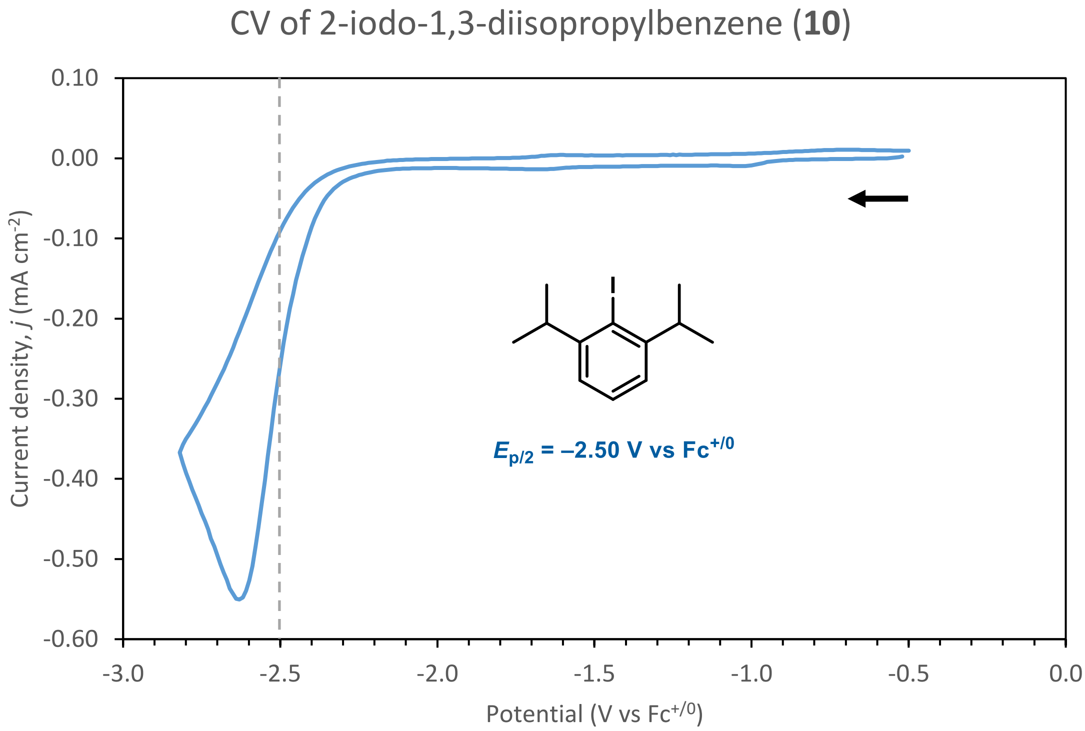

Figure S22. Cyclic voltammogram of a 1 mM solution of 2-iodo-1,3-diisopropylbenzene **10** in CH_3CN using 0.1 M [^{n}Bu$_4$N][PF$_6$] as the supporting electrolyte at ambient temperature; scan rate: 100 mV/s. Potential in V vs $Fc^{0/+}$. Black arrow indicates the direction of the potential sweep. Dashed gray line represents the half-peak potential, $E_{p/2}$.

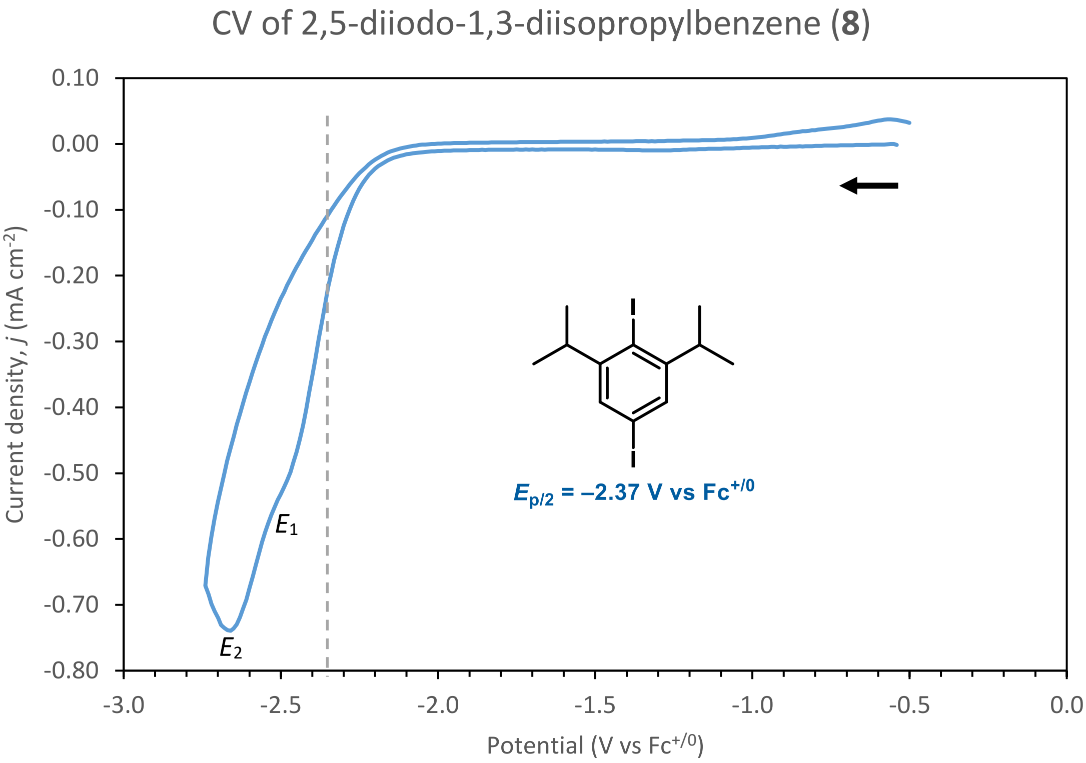

Figure S23. Cyclic voltammogram of a 1 mM solution of 2,5-diiodo-1,3-diisopropylbenzene **8** in CH_3CN using 0.1 M [$^nBu_4N$][PF_6] as the supporting electrolyte at ambient temperature; scan rate: 100 mV/s. Potential in V vs $Fc^{0/+}$. Black arrow indicates the direction of the potential sweep. Dashed gray line represents the half-peak potential, $E_{p/2}$. with respect to the first reduction feature with peak potential E_1.

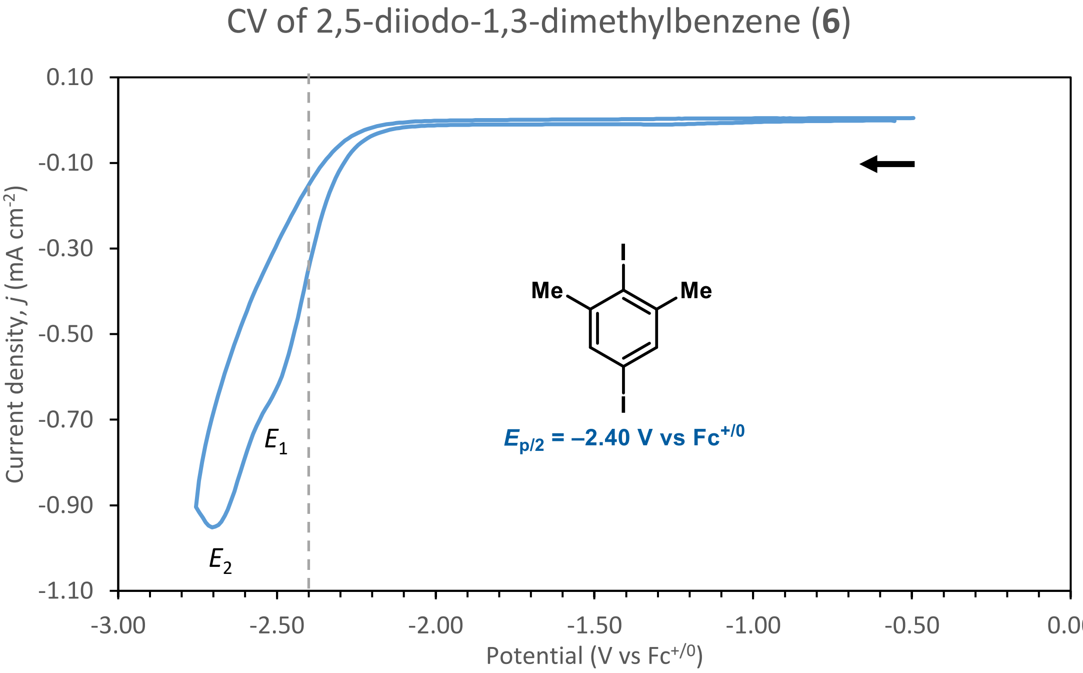

Figure S24. Cyclic voltammogram of a 1 mM solution of 2,5-diiodo-1,3-dimethylbenzene **6** in CH_3CN using 0.1 M [$^nBu_4N$][PF_6] as the supporting electrolyte at ambient temperature; scan rate: 100 mV/s. Potential in V vs $Fc^{0/+}$. Black arrow indicates the direction of the potential sweep. Dashed gray line represents the half-peak potential, $E_{p/2}$ with respect to the first reduction feature with peak potential E_1.

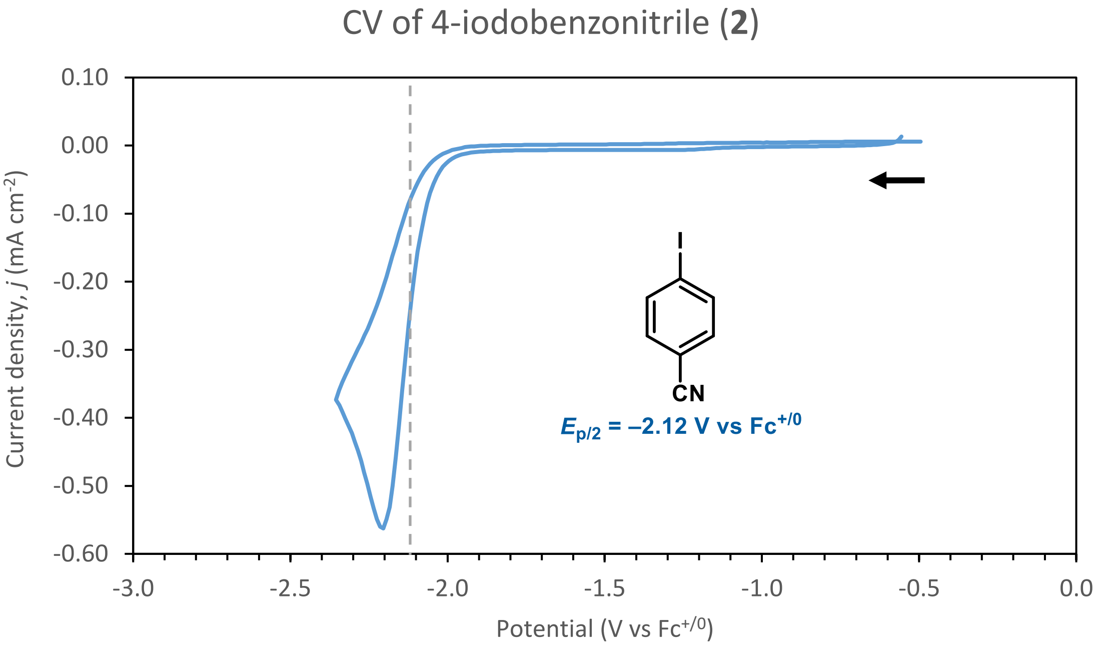

Figure S25. Cyclic voltammogram of a 1 mM solution of 4-iodobenzonitrile **2** in CH_3CN using 0.1 M [$^{n}Bu_4N$][PF_6] as the supporting electrolyte at ambient temperature; scan rate: 100 mV/s. Potential in V vs $Fc^{0/+}$. Black arrow indicates the direction of the potential sweep. Dashed gray line represents the half-peak potential, $E_{p/2}$.

8. Photophysical properties

8.1. UV-Vis absorption and emission spectroscopy

General information

UV-Vis spectra were recorded on a Cary6000i UVVIS/NiR spectrometer, using 2 mm (l = 0.2 cm) Suprasil Quartz cuvettes. All measurements for bismuth complexes were done using solutions of the specified concentration in anhydrous MeCN, stored inside an Ar-filled glovebox, and using the same solvent as blank.

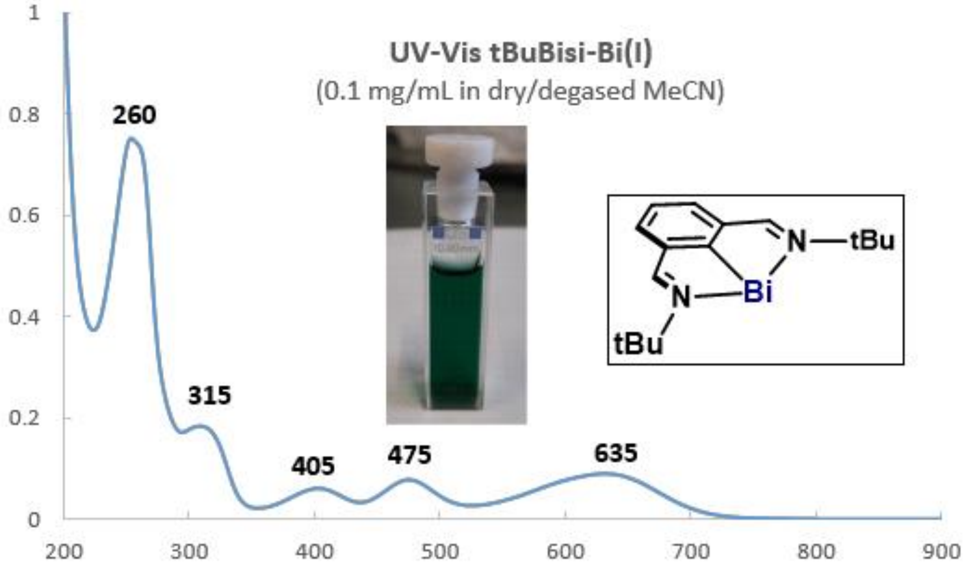

Figure S26. UV-Vis absorption spectrum of bismuthinidene **1a** in MeCN (0.1 mg/mL; 0.2 mM), selected absorption maxima, and physical appearance of a 1 mg/mL solution.

UV-Vis absorption of a mixture of bismuthinidene (1a) and 4-iodobenzonitrile (2)

In order to evaluate potential charge-transfer interaction between the two reagents in solution, we conducted UV-Vis absorption analysis of a solution of bismuthinidene **1a** (0.1 mg/mL in MeCN), a solution of 4-iodobenzonitrile **2** (0.2 mg/mL in MeCN) and a 1:1 mixture of the two solutions (0.05 mg/mL of **1a**, 1 equiv., and 0.1 mg/mL of 4-iodobenzonitrile **2**, 4 equiv.).

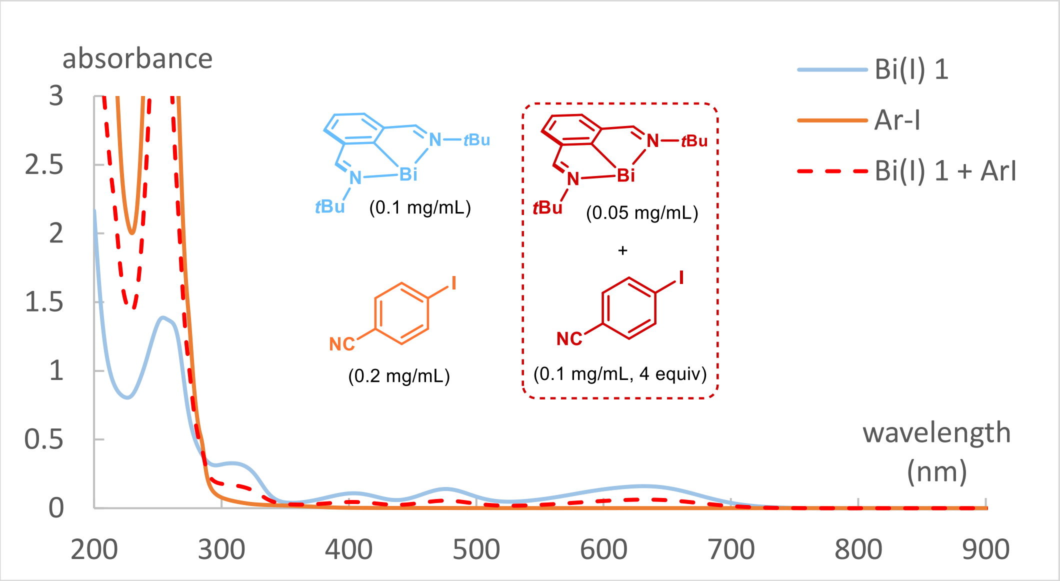

Figure S27. UV Vis absorption spectra of **1a**, **2**, and a mixture of both.

We found no significant shift in the absorption bands. For easy visualization, a concentration-normalized version of the spectrum is attached below.

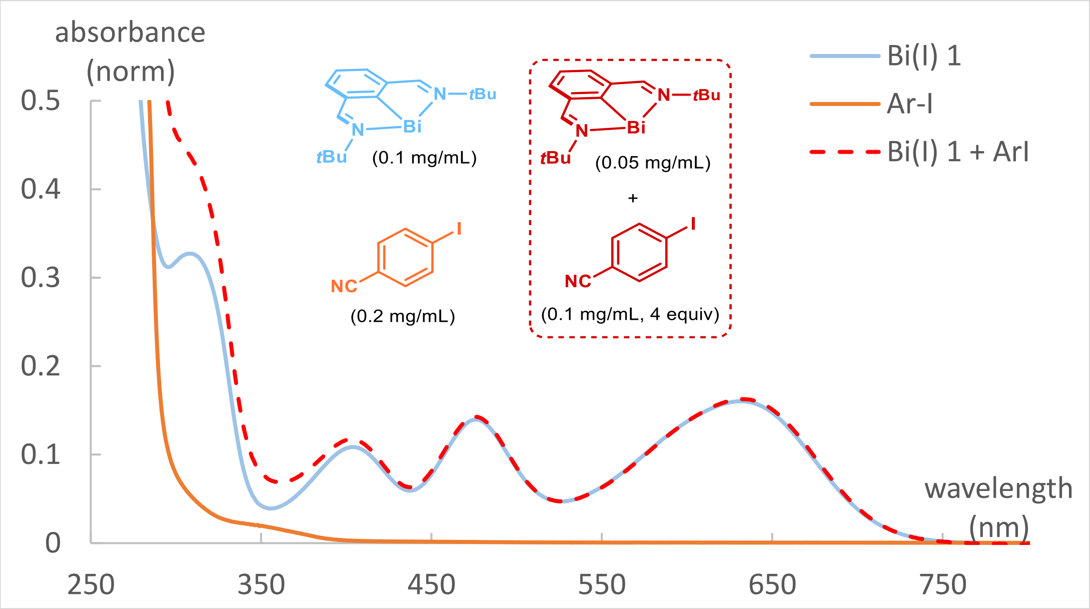

Figure S28. Enlarged section of UV-Vis spectrum (250-800 nm), showing concentration-normalized absorption spectra of **1a**, **2**, and a mixture of both.

Thus, no charge transfer of EDA complex formation can be observed.

UV-Vis absorption spectra in various solvents

In order to obtain additional evidence for the MLCT character of the band at 635 nm, UV-Vis spectra were measured in pentane, diethyl ether, acetonitrile, and dimethylformamide. The overlayed absorption spectra are shown below:

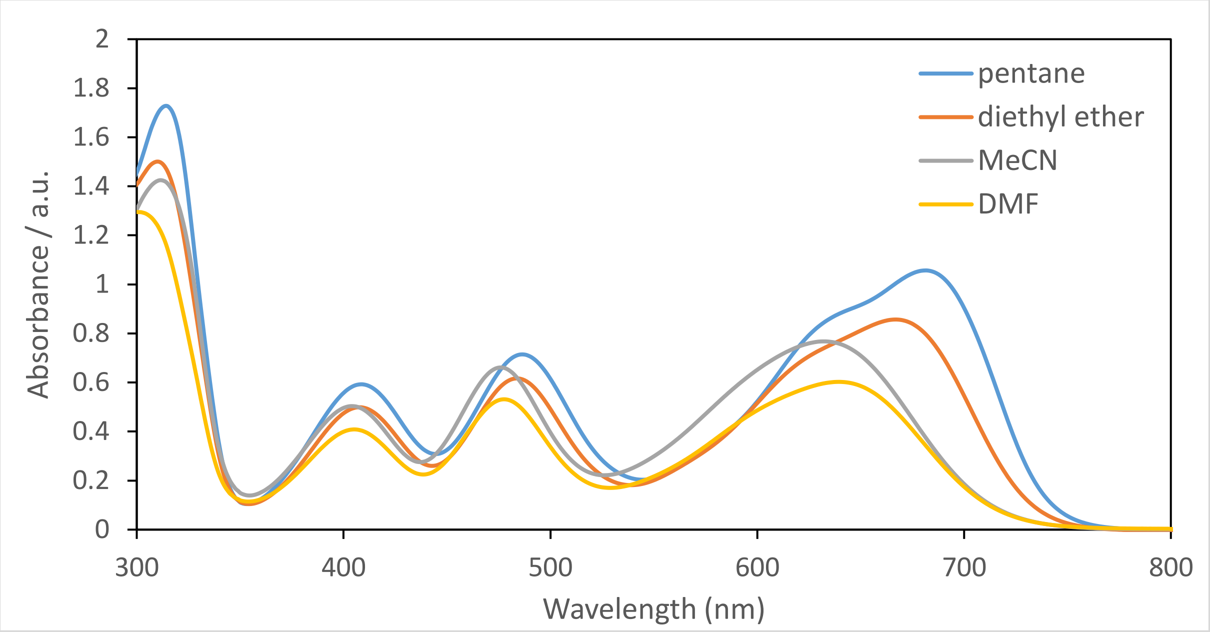

Figure S29. UV-Vis absorption spectrum of **1a** in various solvents: pentane, diethyl ether, acetonitrile (MeCN), and dimethylformamide (DMF), showing strong solvatochromic behavior for the band arising from a Bi(I)→(*N*,*C*,*N*) MLCT transition.

The dramatic solvatochromism of the band at around 630 nm is taken as evidence supporting the assignment of a Bi(I)→(*N*,*C*,*N*) MLCT transition that is responsible for the absorption of red light. The remaining bands show little to no solvatochromic behavior, suggesting predominantly metal-centered transitions. This is supported by quantum mechanical calculations (see Section 11.1 in the Supporting Information).

8.2. Spectroelectrochemistry

Spectroelectrochemical studies were conducted in an optically transparent quartz spectroelectrochemical cuvette (Ocean Optics, path length ca. 1 mm) inside a N_2-filled glovebox (< 1 ppm O_2). This cuvette houses a Pt grid electrode (working), a Pt wire electrode (counter) and a silver wire pseudo reference electrode (*ca.* −0.1 V respect to Fc/Fc^+). The cuvette was filled with ca. 0.5 ml of a 0.11 mM solution of **1a** in MeCN containing 0.1 M [^{n}Bu$_4$N][PF$_6$] inside a N_2-filled glovebox. The UV-Vis evolution during application of a constant potential was measured using a standard Ocean optics sample stage for 1 cm cuvettes and a fiber optic setup calibrated for the measurements. The absorbance spectra were collected using USB2000+UV-VIS-ES spectrophotometer from Ocean Optics (1.5 nm resolution) equipped with a DH-2000 Deuterium-Halogen Light Source and the electrodes were connected to a Biologic SP-300 potentiostat.

The spectroelectrochemical experiments were carried out by applying a controlled potential (−0.6 V vs $Fc^{+/0}$) until a stable spectrum was obtained (ca. 5 min). The chemical reversibility of the process was confirmed by applying the corresponding reductive potential (−1.2 V vs $Fc^{+/0}$), which afforded the initial spectrum. Simultaneously, UV-Vis spectra were recorded continuously to monitor the changes in the electronic structure of the complexes.

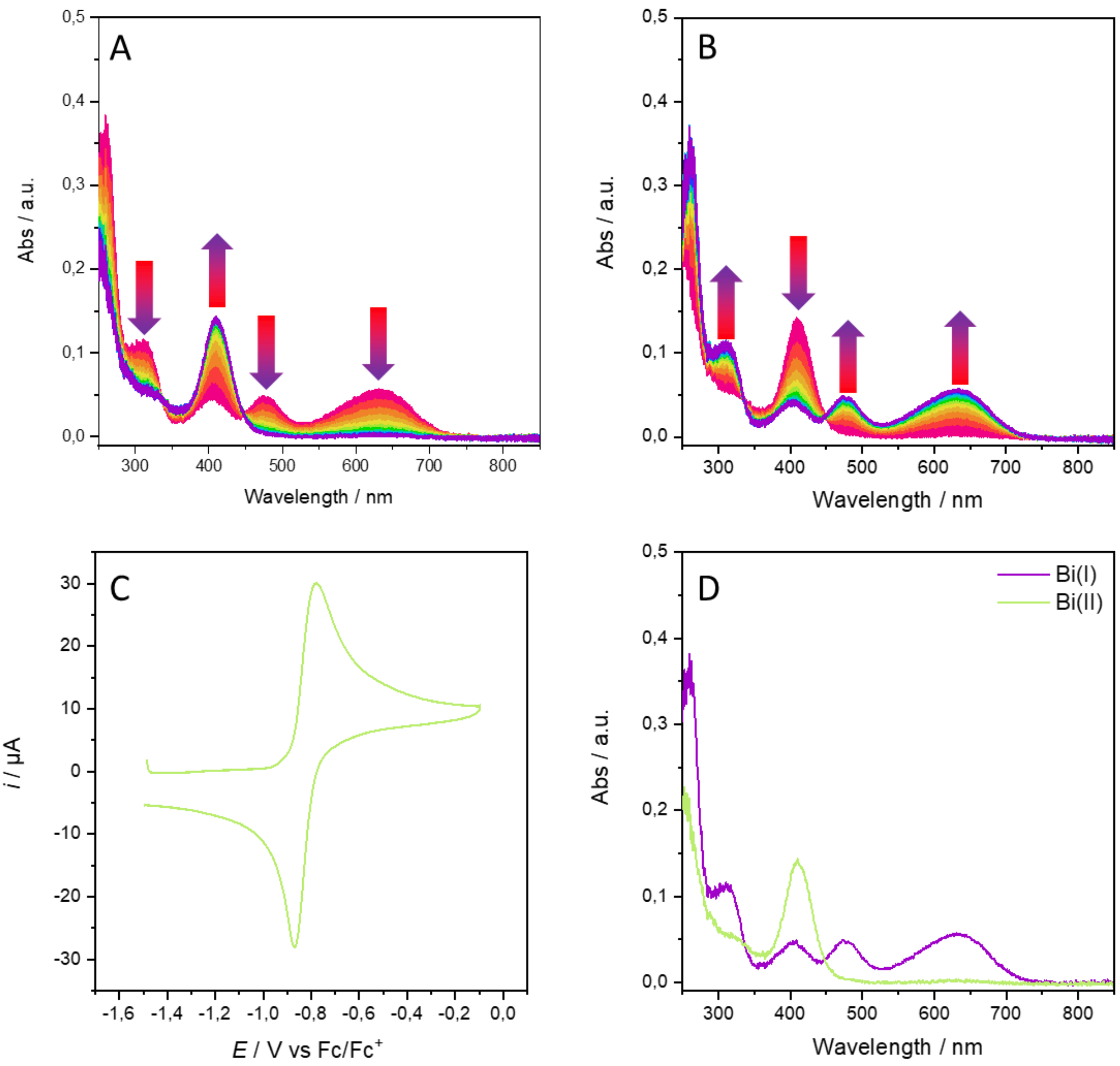

Figure S30. **(A)** UV-Vis evolution of a 0.11 mM solution of Bi(I) (**1a**) in anhydrous MeCN containing 0.1 M of $TBAPF_6$ as supporting electrolyte during a controlled potential electrolysis at −0.6 V vs $Fc^{+/0}$ for 5 min to generate Bi(II) species (**1a**$^+$). **(B)** UV-Vis evolution during a controlled potential electrolysis at -1.2 vs $Fc^{+/0}$ for 5 min under the same conditions to recover the initial Bi(I) species. **(C)** CV of a 0.11 mM solution of Bi(I) in anhydrous MeCN containing 0.1 M of $TBAPF_6$ as supporting electrolyte at a scan rate of 100 mV/s. Pt mesh as working, Pt as counter, silver wire as pseudo-reference. **(D)** UV-Vis absorption spectra of Bi(I) and Bi(II) a solution of in anhydrous MeCN, [Conc.] = 0.11 mM. The Bi(I) spectrum corresponds to the initial species and Bi(II) spectrum was obtained after applying a controlled potential (−0.6 V vs $Fc^{+/0}$) for 5 min.

8.3. Transient absorption spectroscopy

Photophysical analysis: Absorption spectra were collected with a Shimadzu UV-1900i spectrometer. Steady-state emission spectroscopy was performed using a Edinburgh FS5 spectrofluorimeter, using solutions with absorbance of 0.06 at 630 nm. Ultrafast transient absorption experiments were conducted using an Astrella-F-1K amplified Ti:sapphire femtosecond laser system from Coherent, operating at a repetition rate 1kHz, 5.5 W power (5 mJ pulse energy), pulse duration of 80 fs, with TA pump / probe Helios detection system from Ultrafast Systems. White light was generated focusing a fraction of the fundamental 800 nm output onto a 2 mm CaF_2 mounted on a translating crystal holder. A 1.2 mJ fraction of the fundamental is used for pump beam generation by a TOPAS Prime from Light Conversion with standard NirUVis extension. A magic angle configuration between pump and probe polarization directions was used to avoid rotational dynamics. Bandpass filters with ± 5 or ± 10 nm were used to ensure low spectral width and to exclude 800 nm photons. Transient absorption measurements were conducted in a 2 mm quartz cuvette under argon atmosphere, using solutions with absorbances of 0.5-0.7 under stirring. To analyze transient absorption data, we used a suggested procedure.[4] We start with SVD and global analysis, using an all-sequential decay model that provides evolution associated spectra of potentially intervening species, to determine the number of decaying species that participate in the decay cascade. However, this doesn't necessarily yield differential spectra with genuine physicochemical meaning. Afterwards, a target analysis is applied, using specific target models that result in species associated spectra with true physicochemical meaning. Obtained data were treated by SVD, global and target analyses using the R- package TIMP and GloTarAn.[3,5,6]

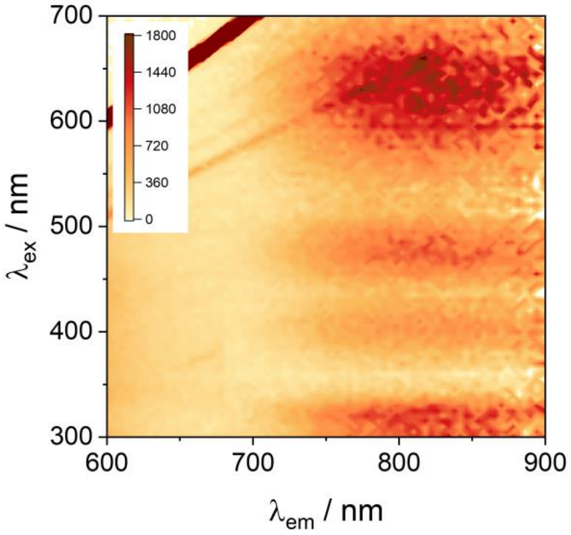

Figure S31. Excitation-emission heatmap of **1a** in MeCN at room temperature, revealing how the maximum position and emission bandshape are independent of the excitation wavelength.

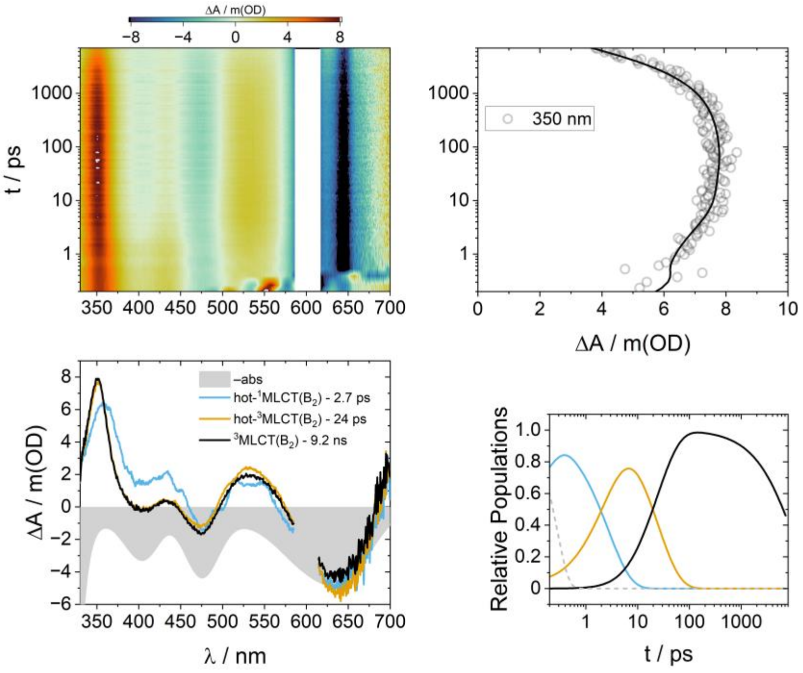

Figure S32. fsTAS experiments on **1a** in MeCN at room temperature using 600 nm excitation. Top left: Differential absorption heatmap. Top right: Differential absorption kinetic trace at 350 nm. Bottom: Species-associated differential spectra (left) and evolution of relative populations (right) of hot-^{1}MLCT(B_2) (cyan), hot-^{3}MLCT(B_2) (orange) and ^{3}MLCT(B_2) (black).

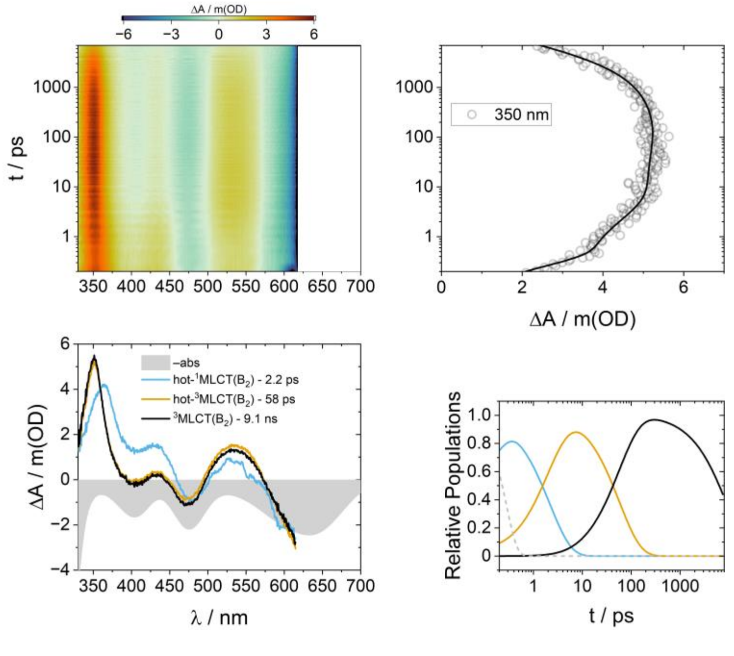

Figure S33. fsTAS experiments on **1a** in MeCN at room temperature using 660 nm excitation. Top left: Differential absorption heatmap. Top right: Differential absorption kinetic trace at 350 nm. Bottom: Species-associated differential spectra (left) and evolution of relative populations (right) of hot-^{1}MLCT(B_2) (cyan), hot-^{3}MLCT(B_2) (orange) and ^{3}MLCT(B_2) (black).

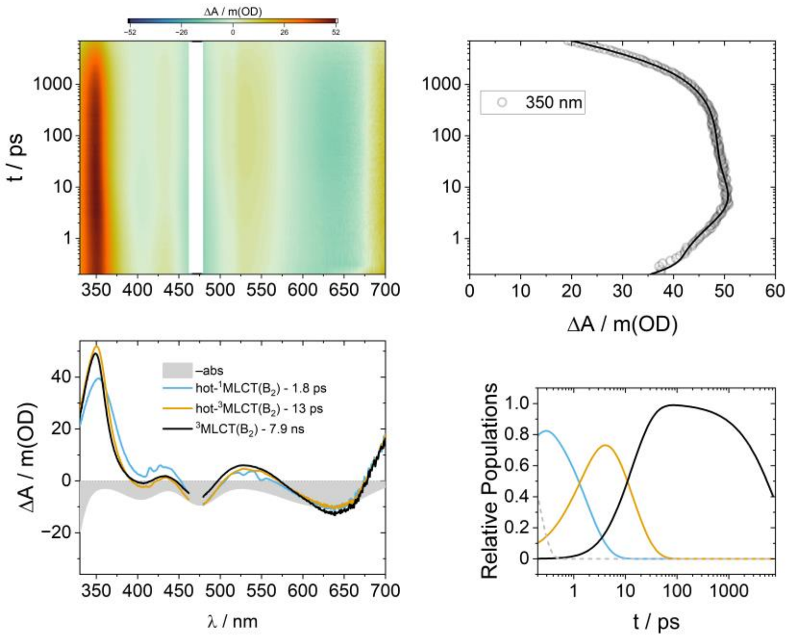

Figure S34. fsTAS experiments on **1a** in MeCN at room temperature using 475 nm excitation. Top left: Differential absorption heatmap. Top right: Differential absorption kinetic trace at 350 nm. Bottom: Species-associated differential spectra (left) and evolution of relative populations (right) of hot-^{1}MLCT(B_2) (cyan), hot-^{3}MLCT(B_2) (orange) and ^{3}MLCT(B_2) (black).

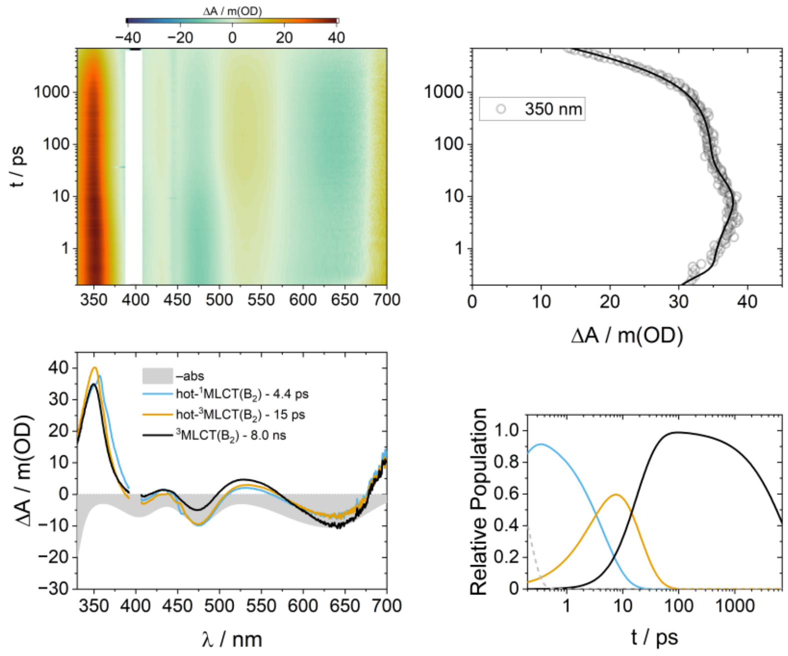

Figure S35. fsTAS experiments on **1a** in MeCN at room temperature using 400 nm excitation. Top left: Differential absorption heatmap. Top right: Differential absorption kinetic trace at 350 nm. Bottom: Species-associated differential spectra (left) and evolution of relative populations (right) of hot-^{1}MLCT(B_2) (cyan), hot-^{3}MLCT(B_2) (orange) and ^{3}MLCT(B_2) (black).

Bimolecular Stern-Volmer quenching fsTAS experiments:

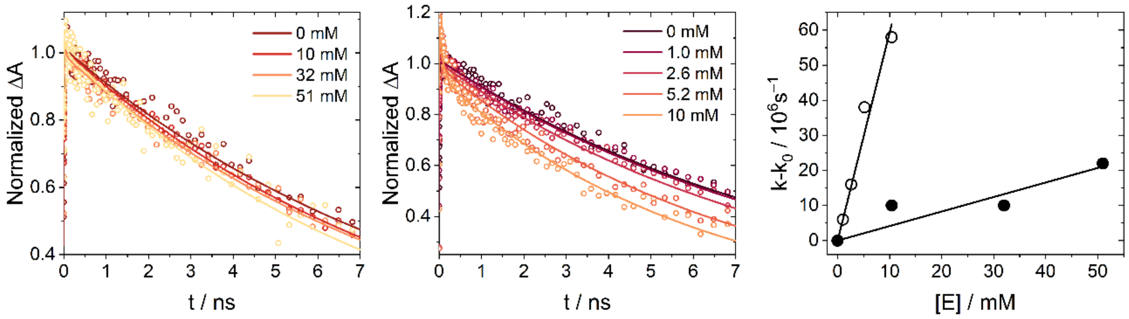

Figure S36. Differential absorption kinetic traces at 350 nm for a 0.55 mM solution of **1a** in MeCN at room temperature, in the presence of different concentrations of **2** (left) and **3** (middle). Right: Stern-Volmer analysis of the bimolecular quenching of **1a** with **2** (full dots) and **3** (empty dots).

8.4. Rehm-Weller estimation of excited-state redox potentials

The schematic below shows the relevant parameters that were used to calculate the redox potentials associated with the excited ^{3}MLCT state.

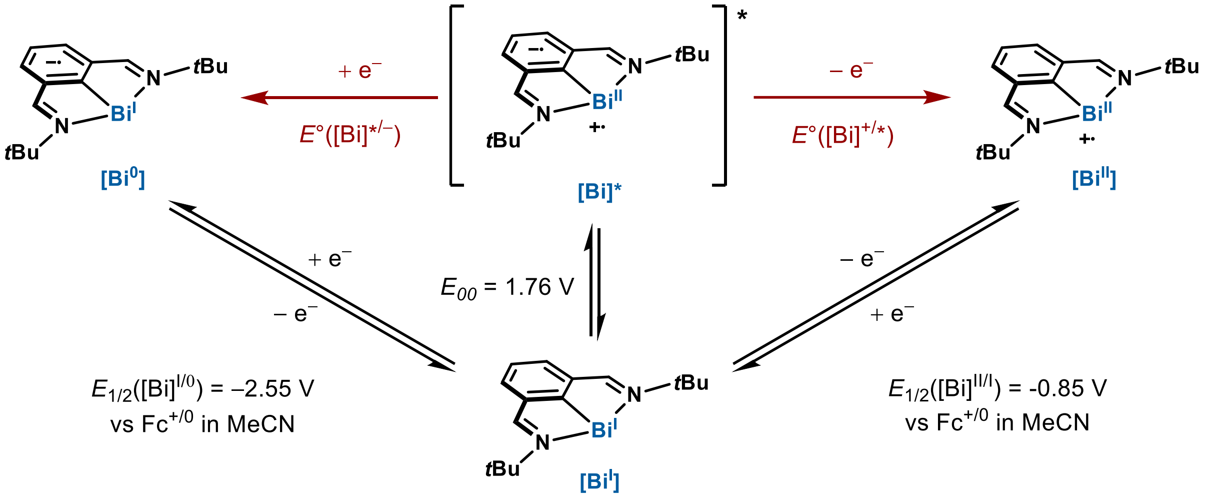

$$E°(Bi^{*/0}) = E_{00} + E_{1/2}([Bi]^{I/0})$$

$$E°(Bi^{*/0}) = 1.76\ V + (-\ 2.55\ V)$$

$$\mathbf{E°(Bi^{*/0}) = -0.79\ V\ vs\ Fc^{+/0}\ (MeCN)}$$

$$E°(Bi^{II/*}) = E_{1/2}([Bi]^{II/I}) - E_{00}$$

$$E°(Bi^{II/*}) = -0.85\ V - 1.76\ V$$

$$\mathbf{E°(Bi^{II/*}) = -2.61\ V\ vs\ Fc^{+/0}\ (MeCN)}$$

The above redox potentials quantify the ability of the excited ^{3}MLCT state to act as an excited-state reductant (−2.61 V vs Fc$^{+/0}$) or oxidant (−0.79 V vs Fc$^{+/0}$).

9. Oxidative additions into alkyl electrophiles

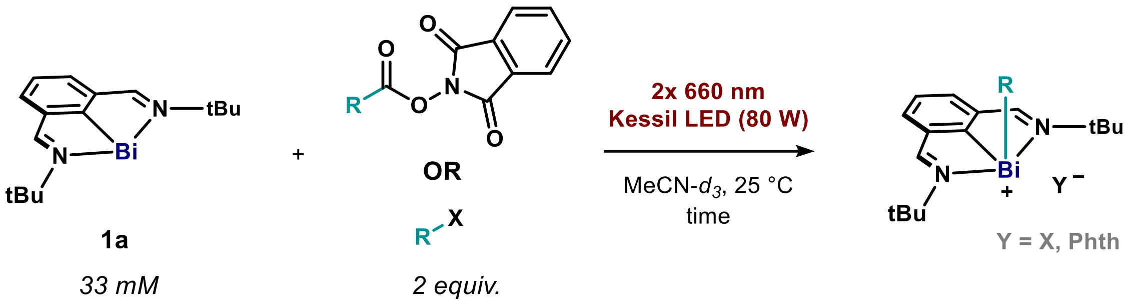

General procedure: While working in an argon-filled glovebox, a 4 mL scintillation vial was charged with **1a** (9.0 mg, 0.020 mmol, 1.0 equiv.), the corresponding alkyl electrophile (0.040 mmol, 2.0 equiv.), and 1,3,5-trimethoxybenzene (10 mg, 0.060 mmol, 3.0 equiv.). The reagents were dissolved in anhydrous, degassed MeCN-d_3 (0.6 mL) and the entire solution was transferred to an NMR tube. The tube was capped and the cap further secured with parafilm before transferring the NMR tube out of the glovebox. The solution was subjected to red light irradiation using the setup outlined in section 2 (2 × 660 nm LED PR160L lamps at 100% intensity, purchased from Kessil), while keeping the temperature at around 35 ºC with the aid of a cooling fan. Full conversion of bismuth(I) was determined visually, by the disappearance of its dark green color to give light yellow/orange solutions. The yield of the oxidative addition was determined by NMR by repeating the reaction in the presence of 1 equiv. of 1,3,5-trimethoxybenzene as internal standard. Unless otherwise stated, full conversion was observed for the starting bismuth(I) complex.

[(2,6-(*t*BuNCH)$_2$C$_6$H$_3$)Bi(4-fluorophenethyl)(phthalimide)] (**18a**)

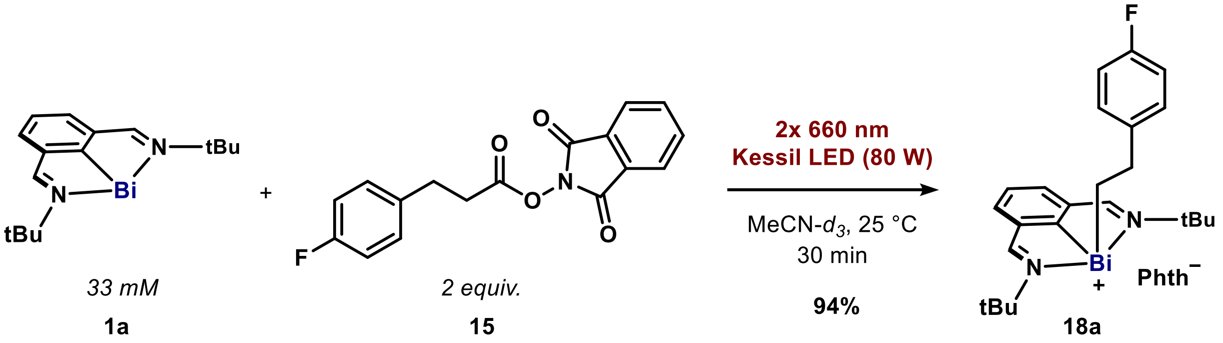

The title compound was obtained following the general procedure outlined above from bismuthinidene **1a** (9.1 mg, 0.020 mmol, 1.0 equiv.) and 1,3-dioxoisoindolin-2-yl 3-(4-fluorophenyl)propanoate **15** (12.5 mg, 0.040 mmol, 2.0 equiv.) in 0.6 mL of MeCN-d_3 (33 mM with respect to **1a**) after 5 min of red-LED irradiation (94% NMR yield). A control reaction showed no conversion even after 24 h of being left in the dark.

^{1}H NMR (300 MHz, CD$_3$CN) δ 9.69 (s, 2H), 8.18 (d, *J* = 7.5 Hz, 2H), 7.91 (dd, *J* = 7.9, 7.1 Hz, 1H), 7.43 (s, 4H), 6.92 – 6.79 (m, 4H), 3.41 – 3.23 (m, 2H), 2.27 – 2.08 (m, 2H), 1.48 (s, 18H).

^{19}**F NMR** (282 MHz, CD_3CN) δ -118.73.

^{13}**C NMR** (101 MHz, CD_3CN) δ 186.37, 169.34, 162.27 (d, $^1J_{C-F}$ = 241.9 Hz), 149.28, 145.52 (d, $^4J_{C-F}$ = 3.0 Hz), 137.11, 131.61, 130.81, 130.47 (d, $^3J_{C-F}$ = 8.1 Hz), 125.89, 120.88, 115.93 (d, $^2J_{C-F}$ = 21.2 Hz), 62.15, 53.88, 33.42, 31.10, 30.59.

HRMS (ESI Positive) calculated for **18a**: $C_{24}H_{31}BiFN_2$ [M-Phth]$^+$: 575.23; found: 575.23.

HRMS (ESI Negative) calculated for **18a**: $C_8H_4NO_2$ [Phth]$^-$: 146.02; found: 146.02.

[(2,6-(*t*BuNCH)$_2$C$_6$H$_3$)Bi(*N*-Boc-3-azetidinyl)(iodide)] (18b)

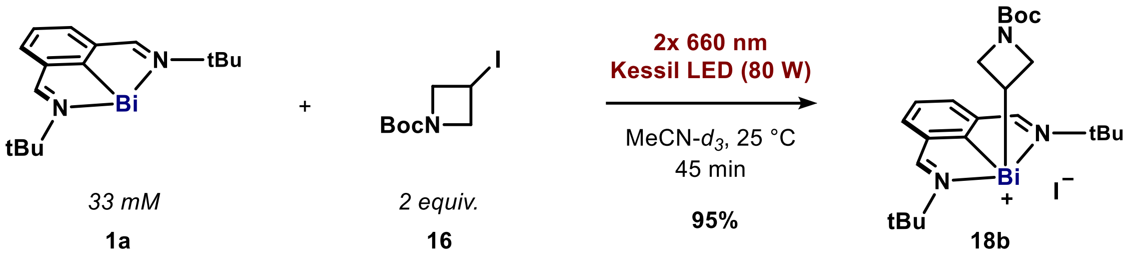

The title compound was obtained following the general procedure outlined above from bismuthinidene **1a** (9.1 mg, 0.020 mmol, 1.0 equiv) and *N*-Boc-3-iodoazetidine **16** (11.3 mg, 0.040 mmol, 2.0 equiv.) in 0.6 mL of MeCN-*d*$_3$ (33 mM with respect to **1a**) after 45 min of red-LED irradiation (95% NMR yield). A control reaction gave only 14% conversion after 3 h of being left in the dark.

^{1}**H NMR** (300 MHz, MeCN-*d*$_3$) δ 9.90 (s, 2H), 8.27 (d, J = 7.3 Hz, 2H), 7.97 (t, J = 7.2 Hz, 1H), 5.23 (t, J = 9.3 Hz, 2H), 4.97 (dd, J = 9.3, 6.7 Hz, 2H), 3.60 (tt, J = 9.1, 6.4 Hz, 1H), 1.47 (s, 18H), 1.25 (s, 9H).

^{13}**C NMR** (101 MHz, CD3CN) δ 187.72, 169.23, 157.56, 149.39, 137.44, 131.17, 79.86, 62.00, 56.82, 31.08, 28.43.

HRMS (ESI Positive) calculated for **18b**: $C_{24}H_{37}BiN_3O_2$ [M-I]$^+$: 608.27; found: 608.27.

HRMS (ESI Negative) calculated for **18b**: I [I]$^-$: 126.90; found: 126.91.

Scale-up synthesis and isolation of 18b

While working in an argon-filled glovebox, a 10 mL Schlenk bomb equipped with a Teflon-coated stir bar was charged with bismuthinidene **1a** (81.4 mg, 0.180 mmol, 1 equiv.) and *N*-Boc-3-iodoazetidine (61 mg, 0.216 mmol, 1.2 equiv.), and the solids were complemented with MeCN-*d*$_3$ (3 mL). The Schlenk bomb was hermetically sealed by screwing the Teflon plug valve, then ported out of the glovebox and the reaction irradiated with 660 nm light (2 × 660 nm LED PR160L lamps at 100% intensity, purchased from Kessil). Within 30 min, the dark green color of the reaction disappeared completely to give a pale-yellow solution. The Schlenk bomb was plumbed to a double-manifold vacuum/argon Schlenk line and the reaction concentrated to dryness on high vacuum (note: gentle heating with a heat gun is required to remove all excess MeCN). The crude was suspended in anhydrous and degassed pentane and sonicated

to remove the crude material from the sides of the flask. The Schlenk bomb was sealed and ported into an argon-filled glovebox. Therein, the suspension was filtered over a fine fritted funnel and the residue washed with additional pentane. Drying the resulting residue afforded **18b** as a golden yellow crystalline powder (98 mg, 74%).

[(2,6-(*t*BuNCH)$_2$C$_6$H$_3$)Bi(*N*-Boc-3-azetidinyl)(bromide)] (18c)

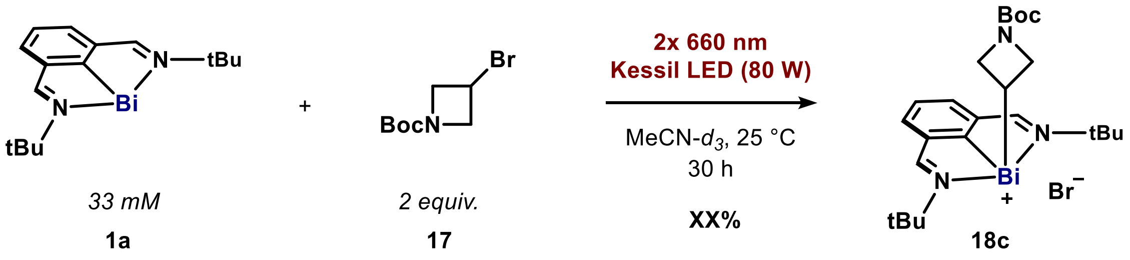

The title compound was obtained following the general procedure outlined above from bismuthinidene **1a** (9.1 mg, 0.020 mmol, 1.0 equiv) and *N*-Boc-3-iodoazetidine **17** (9.4 mg, 0.040 mmol, 2.0 equiv.) in 0.6 mL of MeCN-d_3 (33 mM with respect to **1a**) after 5 min of red-LED irradiation (85% NMR yield).

^{1}H NMR (300 MHz, CD$_3$CN) δ 9.91 (s, 2H), 8.27 (d, *J* = 7.6 Hz, 2H), 8.00 – 7.89 (m, 1H), 5.23 (t, *J* = 9.3 Hz, 2H), 5.00 (dd, *J* = 9.4, 6.8 Hz, 2H), 3.60 (tt, *J* = 9.0, 6.5 Hz, 1H), 1.47 (s, 18H), 1.26 (s, 9H).

^{13}C NMR (101 MHz, CD$_3$CN) δ 188.07, 168.24, 156.60, 148.51, 136.38, 130.09, 79.36, 61.03, 56.43, 30.07, 27.46.

HRMS (ESI Positive) calculated for **18c**: $C_{24}H_{37}BiN_3O_2$ [M-Br]$^+$: 608.27; found: 608.27.

HRMS (ESI Negative) calculated for **18c**: Br [Br]$^-$: 78.92 (100.0%), 80.92 (97.3%); found: 78.92, 80.92.

10. Additional experimental data

10.1. Oxidative addition reactions in MeCN vs pentane

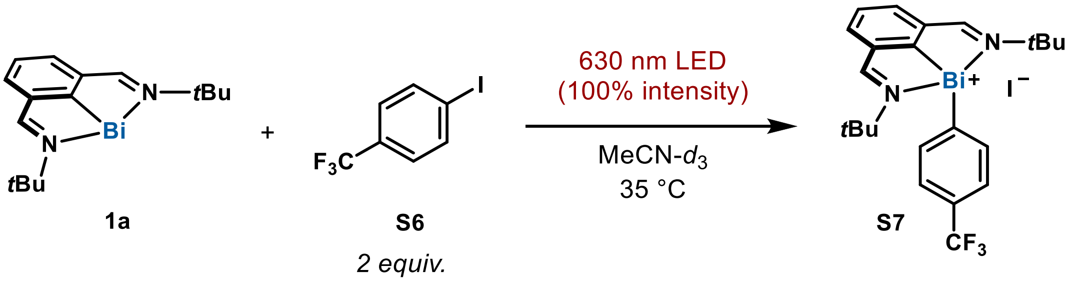

Procedure: While working in an argon-filled glovebox, two separate 10 mL culture tubes equipped with Teflon-coated stir bars were each charged with **1a** (9.0 mg, 0.020 mmol, 1.0 equiv.) and 1-iodo-4-(trifluoromethyl)benzene (10.9 mg, 0.040 mmol, 2.0 equiv.). One tube was complemented with anhydrous, degassed MeCN-d_3 (0.6 mL), while the other was complemented with anhydrous, degassed pentane (0.6 mL). The tubes were hermetically sealed with rubber-lined screw caps, which were further secured with electrical tape. The tubes were ported out of the glovebox and subjected to red light irradiation using the setup outlined in section 2 (2 × 660 nm LED PR160L lamps at 100% intensity, purchased from Kessil), while keeping the solution temperatures at around 35 ºC with the aid of a cooling fan. After 5 h of irradiation, the reaction in pentane was concentrated to dryness on high vacuum, after which both tubes were reintroduced in the glovebox. The concentrated crude reaction mixture was complemented with anhydrous, degassed MeCN-d_3 (0.6 mL), and both solutions were transferred to NMR tubes. The contents of both tubes were analyzed by quantitative ^{1}H NMR. The reactions conducted in MeCN-d_3 and pentane gave 49% and 7% yield of **S7**, indicating a *ca*. 7-fold faster rate of oxidative addition in the latter solvent. ^{1}H and ^{19}F NMR for the previously reported compound **S7** are given below:[3]

^{1}H NMR (S7) (300 MHz, MeCN-d_3) δ 9.76 (s, 2H), 8.34 (d, *J* = 7.6 Hz, 2H), 8.31 – 8.27 (m, 2H), 8.08 (dd, *J* = 7.95, 7.22 Hz, 1H), 7.77 – 7.72 (m, 2H), 1.31 (s, 18H).

^{19}F NMR (S7) (282 MHz, MeCN-d_3) δ −63.49.

Spectrum after reaction in MeCN-d_3

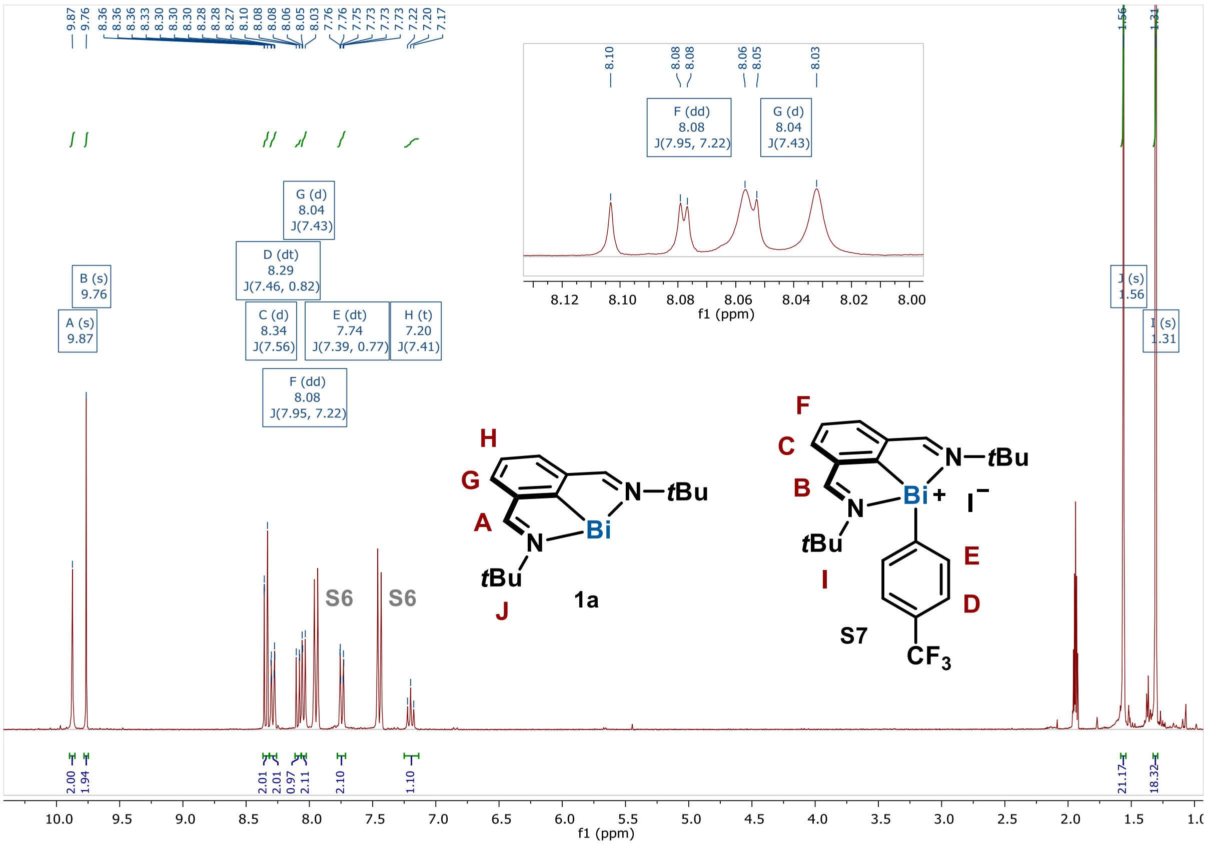

Figure S37: Crude ^{1}H NMR spectrum obtained after red light-promoted oxidative addition of **1a** into **S6** in MeCN-d_3. Spectral assignments for **1a** and **S7** included on spectrum. Excess **S6** labeled accordingly on spectrum (gray labels).

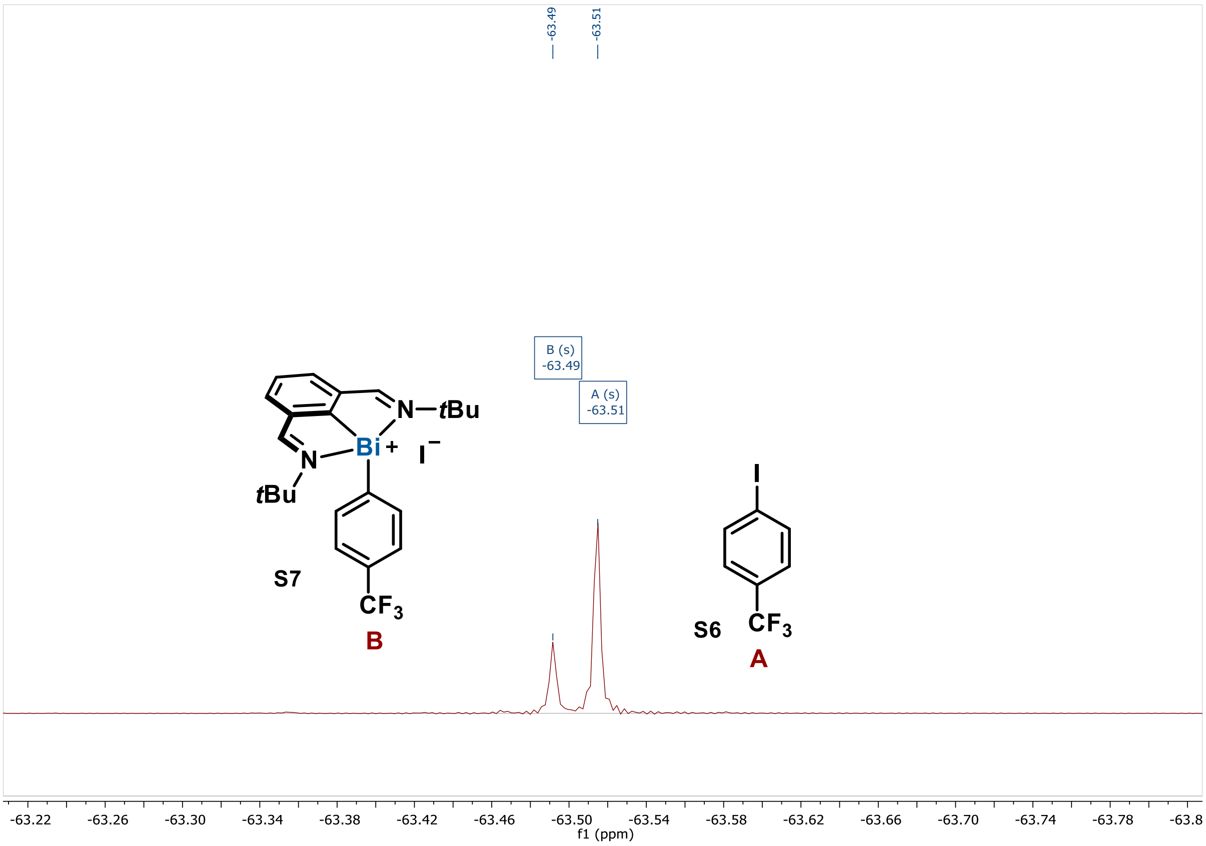

Figure S38: Crude ^{19}F NMR spectrum obtained after red light-promoted oxidative addition of **1a** into **S6** in MeCN-d_3. Spectral assignments for **S6** and **S7** included on spectrum.

Spectrum after reaction in pentane

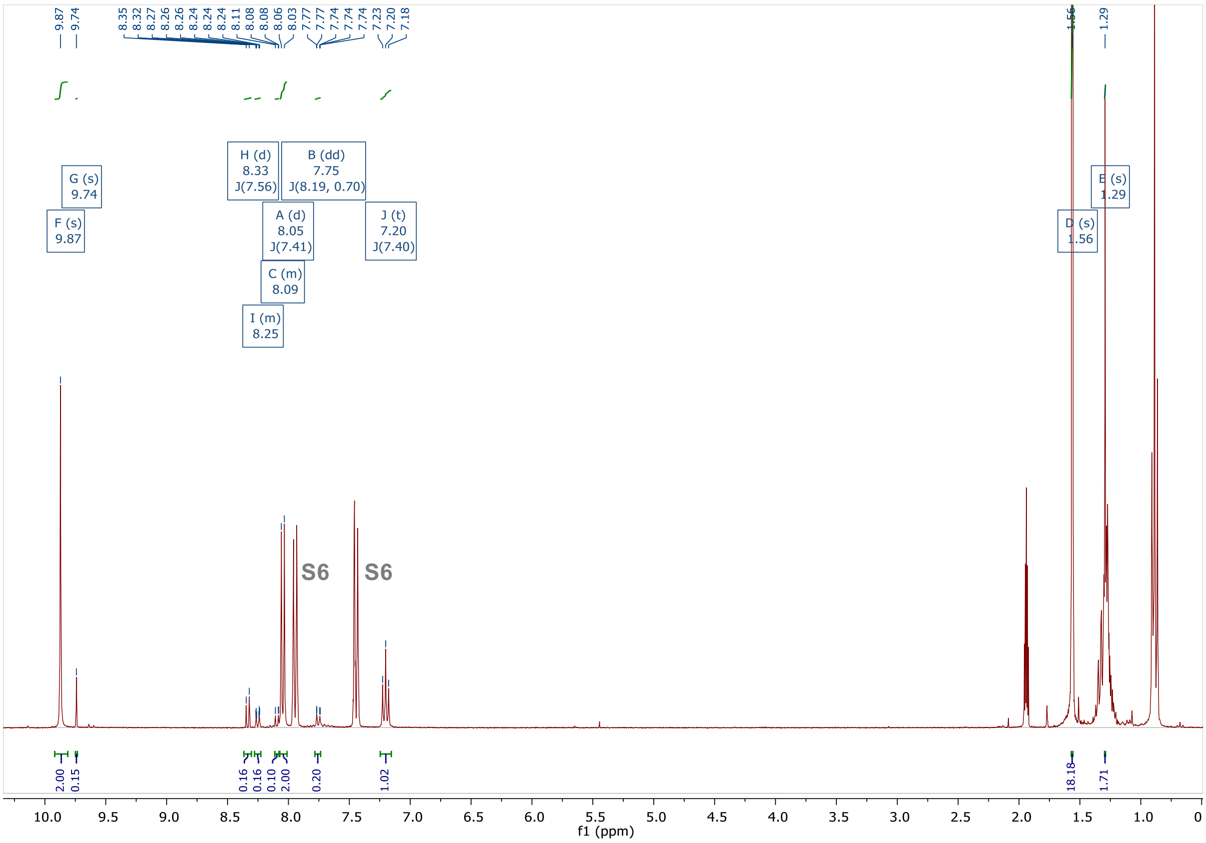

Figure S39: Crude spectrum obtained after red light-promoted oxidative addition of **1a** into **S6** in pentane. For spectral assignments of **1a** and **S7**, refer to Figure 39. Excess **S6** labeled accordingly on spectrum (gray labels).

10.2. Influence of wavelength on oxidative addition

The bismuthinidene **1a** has previously been reported to effect oxidative addition into aryl iodides when irradiated with wavelengths at or below 650 nm, as shown in Figure S40. This is consistent with a complex that follows Kasha's rule, where the formation of the key emissive ^{3}MLCT state is independent of the excitation wavelength. Furthermore, the stability of the product under red-light irradiation is highlighted by the constant concentration of product **3a** after t ≈ 13 h. While the stoichiometric oxidative addition occurs cleanly for red- or green-LED irradiation, under blue light, decomposition of the oxidative-addition adducts occurs at a significant rate, likely initiated by homolytic scission of the Bi-C bond.

The reaction was followed using irradiation from different light colors, with LEDs with an estimated maximum output power of 20 W. The number of photons reaching each reaction may vary due to fabrication of the different light strips, and slight differences in reaction set-up. Hence, this only aims to be a qualitative comparison on selectivity. Reaction performed with 0.010 mmol of Bi(I) and 0.020 mmol of 4-iodobenzonitrile in 0.6 mL of MeCN-d_3 (0.0166 M), in an NMR tube. Yields are determined by ^{1}H NMR using 1,3,5-trimethoxybenzene as a true internal standard.

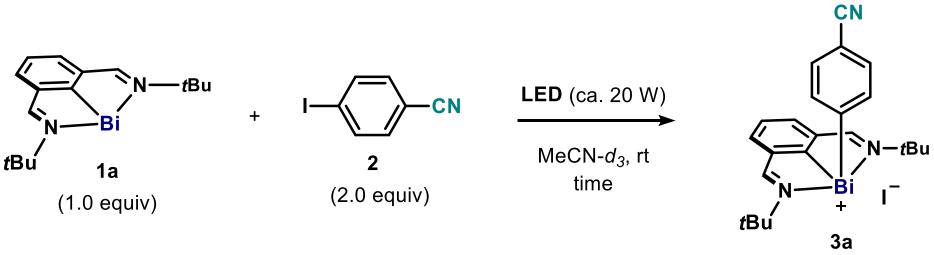

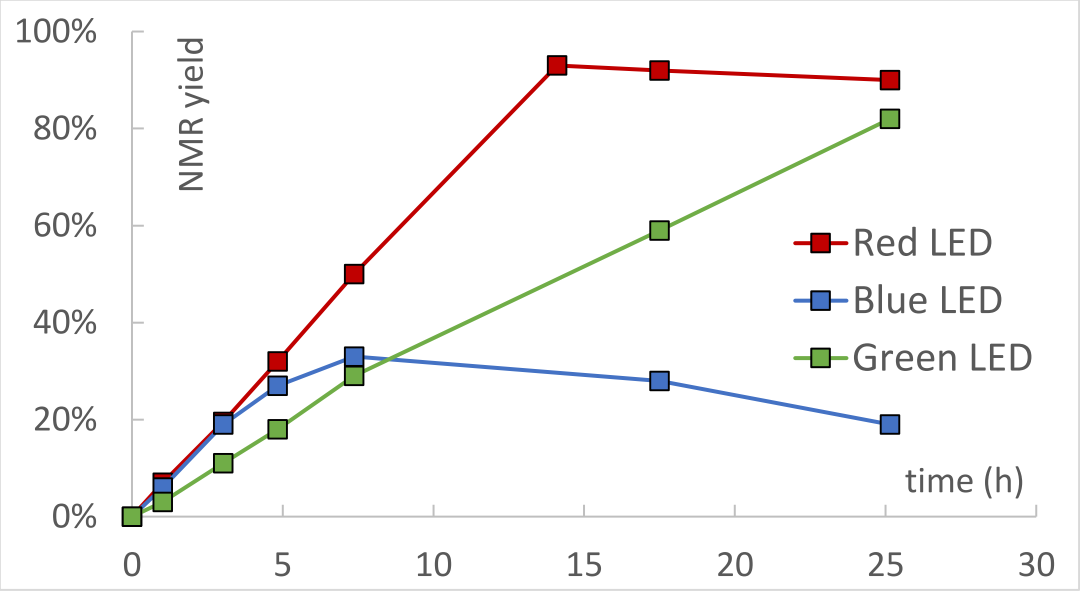

Figure S40. Reaction profiles using different light wavelengths.

For a closer comparison, blue (457 nm, 100 cm/140 LEDs, max. output ca. 19 W; max. luminous flow 400 lumen), green (550 nm, 100 cm/140 LEDs, max. output ca. 20 W; max. luminous flow 1360 lumen) and red (650 nm , 100 cm/140 LEDs, max. output ca. 21 W; max. luminous flow 1000 lumen) LED-strip irradiation was performed with 100 cm of 24V DC LED strips strapped around a 15-cm glass

crystalizing dish. These LED strips were purchased from LEDs24. Nevertheless, the amount of light reaching the sample with each LED is likely not the same.

10.3. Testing for a ground-state interaction via ^{1}H NMR

Complementary to the UV-Vis analysis in Figures S27 and S28, the existence of a ground-state interaction between bismuthinidene **1a** and 4-iodobenzonitrile **2** was probed by ^{1}H NMR in MeCN-d_3.

A 0.01 M solution of bismuthinidene **1a** in MeCN-d_3 (4.5 mg/mL, 0.01 mmol) of bismuthinidene **1a** in MeCN-d_3 was first prepared and measured by NMR. A second 0.01 M solution of 4-iodobenzonitrile in MeCN-d_3 (2.3 mg/mL, 0.01 mmol) was prepared and measured by NMR. Then, a 1:1 mixture of the two solutions was prepared and also measured by NMR.

No significant shift or broadening of the signals was observed, speaking against a ground-state interaction between the two species.

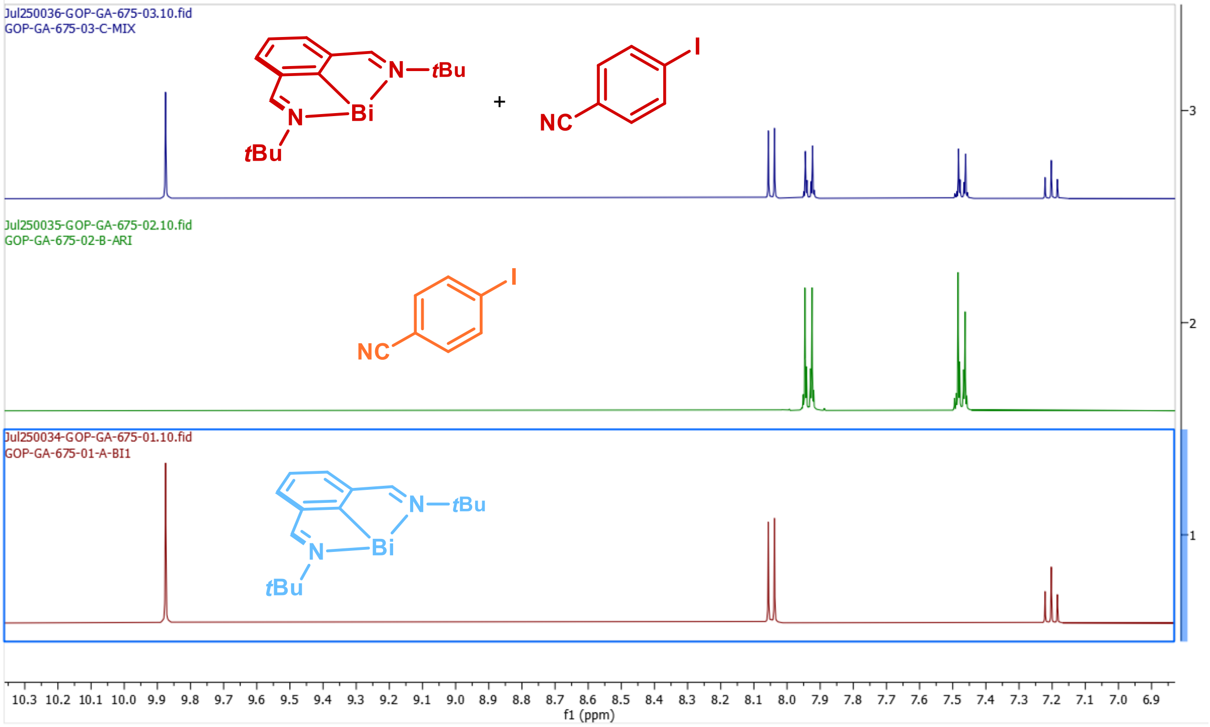

Figure S41. ^{1}H NMR spectra of **1a** (bottom), **2** (middle), and a mixture of both (top).

11. Computational analysis

11.1 Computational studies of **1a**

Computational methods

Quantum chemical calculations were carried out by using the Orca code (v6.0.0 and 6.0.1).[7,8] X2C scalar relativistic Hamiltonian was adopted for all the calculations.[9] Geometry optimizations and subsequent frequencies calculations were carried out within the frame of density functional theory (DFT) using the B3LYP[10,11] functional in conjunction with the X2C-TZVPall-2c basis set.[12] x2c/J auxiliary basis set was employed for the Coulomb fitting in the RIJCOSX approximation.[13,14] Dispersion interactions were treated at the D3(BJ) level.[15] Solvation effects of MeCN were modeled with the SMD method.[16,17]

The electronic structure of **1a** was investigated by means of single-point state-averaged complete active space self-consistent field (SA-CASSCF) calculations performed at the DFT optimized geometry. Dynamic electron correlation was treated using the N-electron valence second-order perturbation theory (NEVPT2).[18,19] Building on our recent work,[3] a slightly larger active space comprising of 6 electrons in 8 orbitals was employed (CAS(6e,8o)). (CAS) comprising of 6 electrons in 8 orbitals was employed (CAS(6e,8o)). This was done in order to include two empty ligand orbitals in the active space that can act as acceptor in Metal-to-ligand charge transfer (MLCT). The active space then consists of the following orbitals:

1. The bonding between Bi $6p_x$ with the two sp^2 orbitals of the N atoms and its antibonding counterpart.
2. The Bi $6p_y$ orbital, bonding to the sp^2 orbital of the C atom and its antibonding counterpart.
3. The Bi $6p_z$ orbital that is forming a weak π-bond with the aromatic ring and its antibonding counterpart.
4. Two empty ligand orbitals that act as electron acceptors for MLCT states.

A graphical representation of the orbitals together with the labels of their symmetry according to the C_{2v} group point is given in Figure S42. The X2C-QZVPall-2c basis set was adopted for Bi, X2C-TZVPPall-2c for C and N atoms and X2C-SVPall-2c for the H nuclei. Spin-orbit coupling (SOC) was treated using the mean-field SOC Hamiltonian, including picture change effects.[20,21] After some experimentation, six singlets and five triplet roots were considered sufficient to account for the SOC effects, in agreement with previous reports.[3]

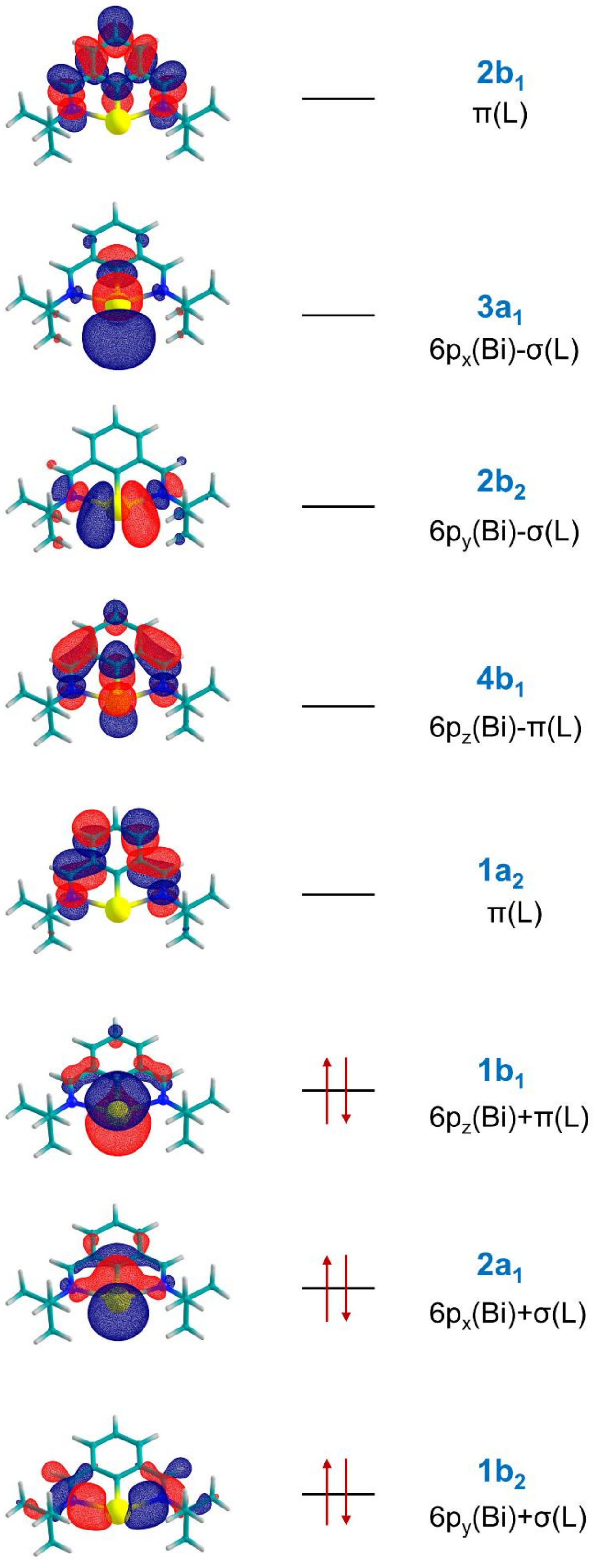

Figure S42. Canonical orbitals optimized with the CASSCF(6e,8o) method. The symmetry of the orbitals reported in cyan is assigned according to the C_{2v} point group. C, N, H, and Bi atoms are in sea green, blue, white, and yellow, respectively.

Bond dissociation free energies (BDFEs) for C-I and C-Br bonds in complexes were calculated as the change of the Gibbs free energy at 298 K of the following reactions:

$$LC\text{-}I/Br \rightarrow LC^{\cdot} + I^{\cdot}/Br^{\cdot}$$

BDFEs were computed on the optimized structures by using the electronic energy calculated at CCSD(T) with the DLPNO scheme[22,23,24,25,26] and the vibrational corrections obtained at DFT level of theory described above. The AUTOAUX keyword was used for the automatic generation of auxiliary basis sets for the RI approximation in the dynamical electron correlation treatment.[27]

11.2 Computational details

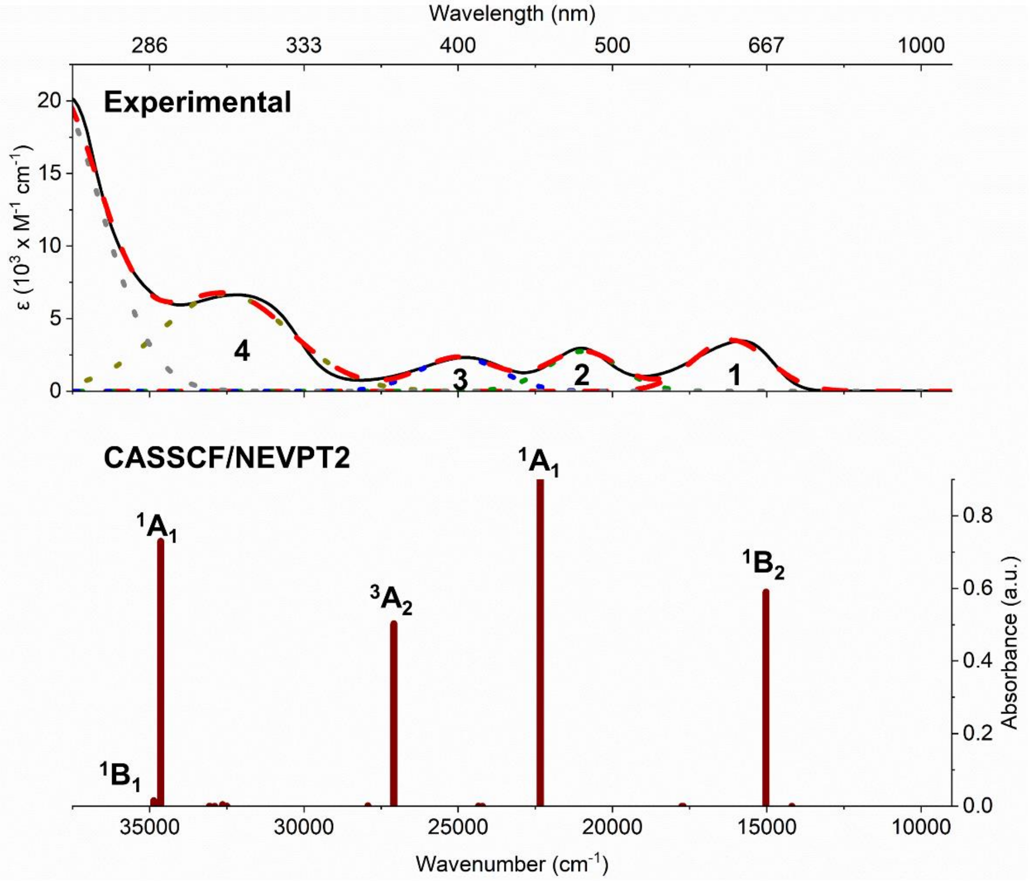

Figure S43. Top: Room temperature UV-Vis spectrum of complex **1a** from 37500 cm^{-1} to 9000 cm^{-1}. Deconvolution of the experimental spectrum into individual bands (dotted lines) is reported in dashed red line; Bottom: CASSCF/NEVPT2 absorbance spectrum with the symmetry of the corresponding excited state.

Table S4. Details of the calculated absorption spectrum presented in Figure S43 for the optimized structure of complex **1a** by the CASSCF/NEVPT2 method.

Band	Excited state symmetry	One-electron excitation	State Energy (cm^{-1})	% purity	f_{osc}(calc)	f_{osc}(exp)
				100	0.000	
**1**	**$^3B_2$**	$1b_1 \rightarrow 1a_2$ ($6p_z$ (Bi)+π(L)→π(L))	14187	100	0.000	0.084
				100	0.000	
	**$^1B_2$**		15022	100	0.092	
			17703	100	0.000	
	**$^3A_1$**	$1b_1 \rightarrow 4b_1$ (($6p_z$ (Bi)+π(L)→$6p_z$ (Bi)-π(L))	17735	100	0.000	
**2**			17754	100	0.000	0.054
	**$^1A_1$**		22336	57	0.155	
			24213	89	0.000	
	**$^3A_2$**	$1b_1 \rightarrow 2b_2$ (($6p_z$ (Bi)+π(L)→$6p_y$ (Bi)-σ(L))	24347	92	0.000	
**3**			27087	62	0.078	0.087
	**$^1A_2$**		27956	79	0.000	
			32501	94	0.000	
	**$^3B_1$**	$1b_1 \rightarrow 3a_1$ (($6p_z$ (Bi)+π(L)→$6p_x$ (Bi)-σ(L))	32637	90	0.000	
			32891	75	0.000	
			33048	100	0.000	
**4**	**$^3A_1$**	$1b_1 \rightarrow 2b_1$ (($6p_z$ (Bi)+π(L)→π(L))	33052	100	0.000	0.208
			33070	90	0.000	
	**$^1A_1$**	$1b_1 \rightarrow 2b_1$ (($6p_z$ (Bi)+π(L)→π(L))	34640	96	0.113	
	**$^1B_1$**	$1b_1 \rightarrow 3a_1$ (($6p_z$ (Bi)+π(L)→$6p_x$ (Bi)-σ(L))	34867	96	0.002	

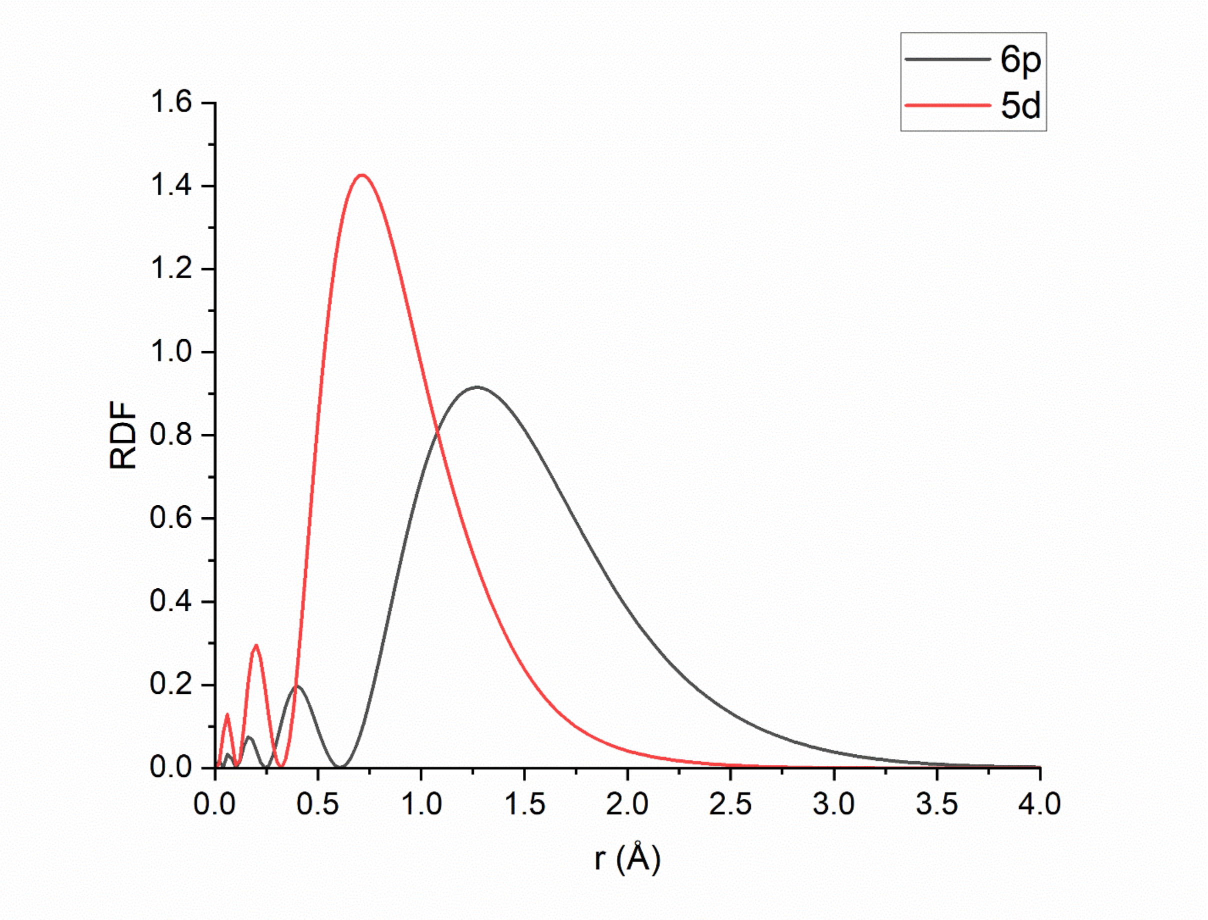

Figure S44. Comparison of radial distribution functions (RDFs) for the 5d atomic orbital of Os(II) (red) and the 6p atomic orbital of Bi(I) (black). The plots illustrate that 6p orbitals are more diffuse than Os 5d orbitals, leading to stronger metal-ligand interactions.

The excited-state ordering can be explained in terms of "primogenic effect". The Bi 6p orbitals extend their maximum amplitudes far away from the nucleus enabling strong overlap with the ligand orbitals (comparison of the radial distribution functions (RDFs) between 6p and 5d orbitals is given in Figure S44). As a result of this, the excited Bi ligand-field states are higher in energy, and thus are not kinetically significant.[28,29]

Table S5. Calculated bond dissociation free energies (BDFEs) at 298 K for C−I and C−Br bonds in compounds described in the main text.

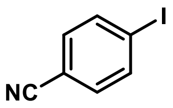

2

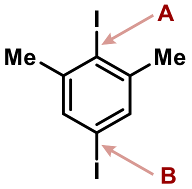

6

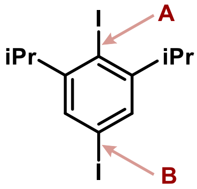

8

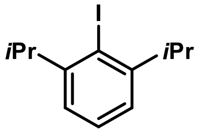

10

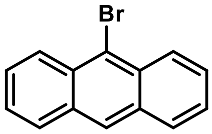

12

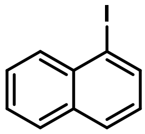

13

Compound	BDFE (kcal/mol)
**2**	61.7
**6-A**	61.4
**6-B**	62.2
**8-A**	55.8
**8-B**	62.4
**10**	55.6
**12**	71.7
**13**	61.9

11.3 Cartesian coordinates

Complex **1a**

C	4.27671661200000	1.10110451600000	-0.49525304100000
C	4.97219047000000	-0.00152841800000	-0.00060914700000
C	2.87898527200000	1.11106589400000	-0.49902599700000
H	6.05453316000000	-0.00226159000000	-0.00140651900000
C	4.27606525400000	-1.10309057100000	0.49529065500000
C	2.16931058500000	0.00000000000000	0.00000000000000
H	4.81870990300000	-1.95973664000000	0.88044771800000
C	2.87827613500000	-1.11095542400000	0.50014451100000
H	4.82070733000000	1.95730649900000	-0.87951462100000
C	2.10547532900000	2.22651317000000	-0.99765433100000
C	2.10395668500000	-2.22300766600000	1.00482279300000
N	0.81985633700000	2.16509892500000	-0.96899536500000
N	0.81829505600000	-2.16045706100000	0.97736055100000
H	2.63787868800000	3.09184794200000	-1.38474567400000
H	2.63561160400000	-3.08613934400000	1.39766094700000
Bi	0.00000000000000	0.00000000000000	0.00000000000000
C	-0.05044671900000	-3.24587288300000	1.47390748100000
C	-0.04857448300000	3.25583391800000	-1.45336747700000
C	-0.91642820700000	3.69833987000000	-0.26833058000000
H	-1.61040711900000	4.48083596100000	-0.58159789700000
H	-1.49382579600000	2.85858512100000	0.12095396100000
H	-0.29375272400000	4.09007509600000	0.53851295700000
C	0.71648310900000	4.45992418500000	-2.00464079600000
H	0.00001676000000	5.21138087800000	-2.33906994400000
H	1.35138532900000	4.91878427700000	-1.24407083600000
H	1.33951919600000	4.18787887100000	-2.85906095900000
C	-0.93897794300000	2.66527436200000	-2.55389446400000
H	-0.33151622500000	2.30310082300000	-3.38573584100000
H	-1.52557945900000	1.82971199000000	-2.16879573800000
H	-1.62553932600000	3.42581774700000	-2.93091194300000

C	0.71450200000000	-4.44201419500000	2.04209997400000
H	-0.00147207000000	-5.18906242400000	2.38733776500000
H	1.35061741800000	-4.91182686600000	1.28928275500000
H	1.33640872400000	-4.15589015300000	2.89267387100000
C	-0.94206848400000	-2.64118085600000	2.56587268200000
H	-1.63359599200000	-3.39472115100000	2.94788659500000
H	-0.33598745900000	-2.27420636300000	3.39663506900000
H	-1.52278668300000	-1.80658958000000	2.17029355900000
C	-0.91667809400000	-3.70246438100000	0.29340709700000
H	-1.49351935800000	-2.86715046600000	-0.10657962600000
H	-0.29258031500000	-4.10298099600000	-0.50802983000000
H	-1.61077844100000	-4.48158646000000	0.61465485500000

Compound **2**

C	-0.92281865113485	-0.68196350397582	-2.51847394233612
C	-2.08128003624499	-1.03751857018352	-1.83560810262359
C	0.23564276384002	-0.32640839741601	-1.83560816887746
H	-2.97671461214215	-1.31221904994927	-2.37457998223184
H	1.13107717894423	-0.05170759789669	-2.37458003002657
C	-2.08246842271074	-1.03772656929015	-0.45053119593546
C	0.23683112089155	-0.32620043604607	-0.45053121777319
H	-2.97684059355872	-1.31198802443207	0.09202759844082
H	1.13120321490135	-0.05193879082207	0.09202750520305
C	-0.92281873126135	-0.68196330933291	0.24505574478360
C	-0.92281913712741	-0.68196309442483	1.67061712822992
N	-0.92281932079806	-0.68196293438282	2.82367300947904
I	-0.92281955459887	-0.68196267584775	-4.62221022233219

Compound **2** radical

C	-0.92281900380665	-0.68196274072175	-2.45271352004324
C	-2.09460683768496	-1.04148701906735	-1.83852274849791
C	0.24896911673010	-0.32243957627365	-1.83852262578682
H	-2.98637918630302	-1.31508841784013	-2.38774973928900

H	1.14074118739341	-0.04883697464817	-2.38774943057630
C	-2.09019527949380	-1.04010804523103	-0.44506463116853
C	0.24455790665639	-0.32381986185851	-0.44506455314435
H	-2.98027998617515	-1.31320064908232	0.10628549540307
H	1.13464258386460	-0.05072744506284	0.10628554260217
C	-0.92281865089932	-0.68196397844406	0.24053685329935
C	-0.92281886141187	-0.68196295415498	1.66731351071520
N	-0.92281894086973	-0.68196198761520	2.82014142248636

Compound **6**

C	1.46632523961147	0.09585499921863	-1.50614813252529
H	1.76404880862882	-0.71416548413035	-2.17447123736649
H	2.05984937078033	0.02839576033075	-0.59617029528222
H	1.71337088893911	1.03171514664972	-2.01103770458812
C	0.00199367305068	0.02319368369432	-1.18569252092622
C	-0.39216728201623	-0.09897497732130	0.14793294051821
C	-1.73524985050268	-0.16744376620345	0.47576643066176
C	-2.71341005355149	-0.11948816985544	-0.50230240093222
C	-2.35949162232208	0.00246069477518	-1.84714980489969
C	-0.99822240665059	0.07361104386250	-2.16311994986555
H	0.36582358095266	-0.13803228768327	0.91685362705446
H	-3.75966005788004	-0.17473087907302	-0.23860665954935
C	-3.44570374559207	0.05253400750080	-2.88134595351293
H	-3.34821620289108	-0.76693965238679	-3.59600365034217
H	-4.42439165742024	-0.01570787354633	-2.40977034221613
H	-3.40163854378289	0.98064408836534	-3.45425698868576
I	-0.43117280551751	0.26976423119670	-4.19654904108948
I	-2.30197486683616	-0.34472597439399	2.50152182754724

Compound **6-A** radical

C	1.45257545917058	0.09173589760592	-1.54677694867842
H	1.71107050032511	-0.70687361226392	-2.24420593006437
H	2.09483786141353	0.00615663206311	-0.67141321490072
H	1.67224656616135	1.03912525337006	-2.04270417712838

C	0.00298054456369	0.01561082005457	-1.15591178605651
C	-0.39230384960956	-0.09643351528103	0.18631707862524
C	-1.74273073276749	-0.16014703587224	0.50272059174365
C	-2.73332722184603	-0.11736352655530	-0.46912234925087
C	-2.37609605615434	-0.00514518528473	-1.82199292671893
C	-1.02564617437905	0.05291110817516	-2.06518449681069
H	0.36226736172623	-0.13242721846782	0.96029794234753
H	-3.77924917822825	-0.16977115115312	-0.19885259105650
C	-3.41296930478865	0.05184212669783	-2.90905999900497
H	-3.25016240132846	-0.73629348520945	-3.64610211193248
H	-4.41418485701308	-0.06345027595974	-2.49631245988527
H	-3.36806706277389	1.00538892212412	-3.43881211290136
I	-2.30995645447170	-0.32666475404341	2.53311449167307

Compound **6-B** radical

C	1.47096352632102	0.09682473720919	-1.50846307751470
H	1.76663838575901	-0.71475775258419	-2.17586754768160
H	2.06743698097914	0.03157569873663	-0.60021353431252
H	1.71531269185855	1.03199446829170	-2.01595173468566
C	0.00804215432076	0.02358574037845	-1.18173548672815
C	-0.38358094140954	-0.10042907795678	0.16264854212254
C	-1.72170377640519	-0.16467858611726	0.42773339936669
C	-2.72833566698880	-0.12126181174567	-0.49409853620659
C	-2.36677414151423	0.00268010828860	-1.84682033147471
C	-1.00129498771268	0.07301776314568	-2.15210573198252
H	0.37137177633852	-0.14067902899854	0.93719829097312
H	-3.77490208497001	-0.17753576945489	-0.22400931959858
C	-3.44849838031462	0.05346790171162	-2.88573067772288
H	-3.34957333084894	-0.76732821572858	-3.59873515324590
H	-4.42893337739478	-0.01282250113169	-2.41743863213166
H	-3.40083805574878	0.98101211607410	-3.45928337255310
I	-0.43324377226941	0.26802520988161	-4.18919909662378

Compound **8**

C	1.48577654801840	-0.36343795259855	-1.62997064324413
H	1.65509254335756	0.09262901037374	-2.60439301831320
C	-0.00091254157006	-0.26329381394714	-1.33405290203237
C	-0.40517825878862	-0.22392754405874	0.00019875185154
C	-1.74679290137027	-0.17525745392526	0.32233091464888
C	-2.71176785673514	-0.15826833963135	-0.66549567883745
C	-2.36246557105117	-0.20002726719081	-2.01899034015902
C	-0.99379661741874	-0.25887264855069	-2.32980867032303
H	0.33754342735088	-0.23465709981737	0.78189451716607
H	-3.75810242464802	-0.11295412498631	-0.40098469025462
C	-3.54340218956371	-0.17783706473569	-2.98745576209326
H	-4.41774093165801	-0.10412260164159	-2.33988552869793
I	-0.34494335462223	-0.36692843036023	-4.36015664830102
I	-2.33079590154916	-0.12362526293378	2.34995649379438
C	1.90211235441691	-1.83745529342843	-1.72449697635990
H	2.95352857163282	-1.91917757558648	-2.00692779850084
H	1.76915116367684	-2.33302025945829	-0.76030419700027
H	1.30837829858995	-2.37205564832048	-2.46654318160877
C	2.36988004915061	0.38429356352042	-0.63048836813298
H	2.04788642861097	1.41923909150342	-0.50495343231283
H	2.37940392147597	-0.09179199727144	0.35106810879957
H	3.39813757776479	0.39278820829328	-0.99549588792654
C	-3.59189017250019	1.06247331740721	-3.88756374869647
H	-2.81400487782460	1.05806026463008	-4.64754717811414
H	-4.55689169806691	1.10162078189714	-4.39699681622025
H	-3.48613177793044	1.97487907090733	-3.29812904588858
C	-3.72175596939238	-1.48322757017272	-3.77268160810081
H	-4.67217381413694	-1.45588514023465	-4.30959795199192
H	-2.93059900898464	-1.64417322381005	-4.50191660630894
H	-3.74163656223440	-2.34094658487255	-3.09812243584105

Compound **8-A** radical

C	1.33986120948687	-0.54254490056508	-1.74424250800627
H	1.33663547184436	-0.46268861518726	-2.83303993623072
C	-0.10585438944700	-0.45634215389366	-1.29444999667165
C	-0.46308256362164	-0.51436633085244	0.06417249257718
C	-1.79896314282071	-0.43111357766123	0.42186654052435
C	-2.81397758571939	-0.29170615923397	-0.51913466971977
C	-2.49470424782782	-0.23088651111987	-1.88101931974515
C	-1.15130997863191	-0.31888536614375	-2.16742298885152
H	0.30432339440089	-0.62241045725047	0.81781490154244
H	-3.84942000988954	-0.22882265251064	-0.21251274767476
C	-3.56334129864346	-0.08362812715404	-2.94149469428424
H	-4.52214696008973	-0.01012965367215	-2.42393684325814
I	-2.31700801147852	-0.51434922266853	2.47136982457581
C	1.95784598949357	-1.89310011996190	-1.36833512290298
H	2.98002636080560	-1.95990558390309	-1.74565602496205
H	1.99079179999103	-2.02081978829629	-0.28456202667056
H	1.38527293176460	-2.72099122733621	-1.78952046599484
C	2.16716638776320	0.61896632612567	-1.18453244724076
H	1.74090011193156	1.58198073522995	-1.47013019745246
H	2.21319214189510	0.57866582609869	-0.09451526249178
H	3.18915087401963	0.57292306344957	-1.56540610810141
C	-3.35671495675917	1.19638698506896	-3.75678067254720
H	-2.40553949454609	1.16307009235333	-4.29255923632181
H	-4.15558316615941	1.31388375038852	-4.49136804701481
H	-3.35191412175270	2.07867143628892	-3.11469559351570
C	-3.60182127821101	-1.31517733971354	-3.85189479555528
H	-4.40285753831614	-1.22164842844808	-4.58767148309402
H	-2.65792119646269	-1.42589427439539	-4.39023009003136
H	-3.77215673301946	-2.22716972503601	-3.27746748088049

Compound **8-B** radical

C	1.48996438544252	-0.36364398145717	-1.62880491883359
H	1.65594090252538	0.09457284943137	-2.60292994494709

C	0.00463496935972	-0.26129932677908	-1.32726446005977
C	-0.39833912090140	-0.21845860292432	0.01778957564055
C	-1.73499148872497	-0.17044091973297	0.27679299387026
C	-2.72802267026488	-0.15444804803895	-0.65632242978033
C	-2.37012449537872	-0.19962630939247	-2.01778950404781
C	-0.99719530997116	-0.25745415747318	-2.31636143623961
H	0.34011251086839	-0.22722582197215	0.80647862234957
H	-3.77497137681916	-0.10775003903356	-0.38645010804715
C	-3.54730673576523	-0.17968191004920	-2.99133469071261
H	-4.42345704110179	-0.10816677669402	-2.34593105303150
I	-0.34783240152489	-0.36467738876862	-4.35041710221803
C	1.90385872076745	-1.83807558599431	-1.72728001333642
H	2.95415661466085	-1.92015200815573	-2.01373522832363
H	1.77410135411838	-2.33500758737563	-0.76338899611538
H	1.30667328975459	-2.37062240404898	-2.46792854190357
C	2.37757484470282	0.38088813427167	-0.63048586818824
H	2.05960466615109	1.41701019719213	-0.50457858752728
H	2.38620166232396	-0.09579559034741	0.35085794278653
H	3.40554365690855	0.38551347487499	-0.99639891770278
C	-3.59620574480974	1.06172179090027	-3.89013717725389
H	-2.81698258597466	1.05989909445085	-4.64870361704504
H	-4.56071307954266	1.09888699583848	-4.40073849785145
H	-3.49379684026000	1.97354696010551	-3.29926373377043
C	-3.72131404413627	-1.48501321789753	-3.77767717777520
H	-4.67141674510513	-1.45923183022781	-4.31528944356293
H	-2.92916104502420	-1.64328519308846	-4.50630419781417
H	-3.73983385227884	-2.34331779761370	-3.10387148855898

Compound **10**

C	1.34384696700976	-0.35183532038151	-1.13557559168600
H	1.51337559745728	0.10497228046397	-2.11068564111280
C	-0.14180112047156	-0.25140670751514	-0.83477152979725
C	-0.55505376344592	-0.21042500568520	0.49751782428949

C	-1.89573348146259	-0.15996554553119	0.83423939769679
C	-2.85038015962518	-0.14492697192935	-0.16677583934192
C	-2.50781011772430	-0.18797776852409	-1.52240651673159
C	-1.13759592468911	-0.24705455839906	-1.82825848488771
H	0.18860557042173	-0.22012256879306	1.28180347915777
H	-3.89978888770319	-0.09895941097571	0.09672679434897
C	-3.68469094969175	-0.16637078008071	-2.49525848951942
H	-4.56089784832685	-0.09354943862531	-1.84809234995623
I	-0.48733208836815	-0.35606000777824	-3.86403831422377
H	-2.19661880781225	-0.12900579731029	1.87405333336138
C	1.76268588810549	-1.82500421104387	-1.23231658171270
H	2.81451364469709	-1.90589468355470	-1.51709756514556
H	1.63196108517231	-2.32260052793487	-0.26785698436482
H	1.16758663460133	-2.35989663059411	-1.97432556809226
C	2.23090677040896	0.39558143144107	-0.13815166106735
H	1.91020798074791	1.43196275758355	-0.01331581901147
H	2.24062756230457	-0.07938220603040	0.84478701238780
H	3.25965622194845	0.40267470276329	-0.50458017789774
C	-3.73560343621776	1.07457592587261	-3.39472651206384
H	-2.95627386861149	1.07359806542117	-4.15454358374665
H	-4.70097821799111	1.11238697024690	-3.90549560041431
H	-3.63269278653567	1.98714466336998	-2.80336556381045
C	-3.86435593935459	-1.47047385948053	-3.28248814244786
H	-4.81529611033998	-1.44243342700091	-3.82028811076971
H	-3.07263184112331	-1.63199983427072	-4.01228031262930
H	-3.88503695738009	-2.32953211172358	-2.60819467681147

Compound **10** radical

C	1.19712646386382	-0.53250501336928	-1.25096641344956
H	1.19144864311050	-0.45033597104559	-2.34065038379693
C	-0.24656016277343	-0.44745424833015	-0.79414219200671
C	-0.61110987152828	-0.50568374210453	0.56271974237209
C	-1.94598627683457	-0.42638081495209	0.93646969618839

C	-2.95199747218236	-0.28844349778325	-0.01800387317749
C	-2.64013080178634	-0.22542873970475	-1.38192411833047
C	-1.29506809271158	-0.31110320010763	-1.66530041752088
H	0.15807548738213	-0.61352134034036	1.31848289042412
H	-3.99064275862738	-0.22795598827101	0.28844309692757
C	-3.70450336162266	-0.07763918915836	-2.44669022064403
H	-4.66741156743555	-0.02080703240312	-1.93214770838240
H	-2.21058645244551	-0.47248551806995	1.98581691846280
C	1.81790144045128	-1.88379555137058	-0.88159382163781
H	2.83974026799630	-1.94992615136266	-1.26268219530453
H	1.85420383460038	-2.01530700292785	0.20245122754207
H	1.24358666412338	-2.71117812711882	-1.30375238799039
C	2.02870327125519	0.62694076963452	-0.69304384895825
H	1.60203241549971	1.59154952157752	-0.97611021771190
H	2.07841252592746	0.58550660346708	0.39759074828933
H	3.05029186865264	0.58093114232179	-1.07765309820282
C	-3.51150243971962	1.21477616576789	-3.24595319933849
H	-2.55672139716007	1.20073445661011	-3.77796939226987
H	-4.30894239381153	1.33209598058140	-3.98351439198763
H	-3.52070326523149	2.08963887793151	-2.59233036435534
C	-3.72832671403860	-1.29690092024862	-3.37386826128541
H	-4.52751459178374	-1.20192078971122	-4.11282649608710
H	-2.78040705027828	-1.39237423378779	-3.90983931946616
H	-3.89247411889176	-2.21868542972422	-2.81167928230218

Compound **12**

C	-1.24536267651124	-0.54997821861695	-5.28323369615997
C	-1.24536153052353	-0.54515217497381	-3.85850005654630
C	-1.24536081887848	-1.73456844312467	-3.13739668408323
C	-1.24536114195263	0.71744058013384	-3.16217917819099
C	-1.24536157606044	0.68828902029545	-1.76231470757649
C	-1.24536123540583	-0.49864337056675	-1.01963291341341
C	-1.24536065177636	-1.74386848826666	-1.74652586283805

C	-1.24536314517014	0.61359441793079	-5.99164083270837
C	-1.24536137707770	1.90839401743648	-3.94498119948010
Br	-1.24536252990242	2.35987576919247	-0.81330272873233
C	-1.24536169004188	-0.55983918923119	0.40416161746086
C	-1.24536093480704	-2.96954765490055	-1.02028162736032
C	-1.24536254048125	-2.98089175551814	0.34199133196759
C	-1.24536205785122	-1.75654004891287	1.05912849198575
H	-1.24536121355360	0.35940858852099	0.97038781321224
H	-1.24536082327071	-3.89440623812468	-1.58411582114493
H	-1.24536350007474	-3.91831274960909	0.88326047619952
H	-1.24536310500015	-1.77422282351947	2.14164282254211
C	-1.24536159327659	1.85705646375803	-5.30826305467083
H	-1.24536273578470	-1.50818484219393	-5.78845885829715
H	-1.24536127211591	-2.67663816302554	-3.67228938542622
H	-1.24536240433595	0.59732759108849	-7.07395837387208
H	-1.24536178257230	2.86605804699583	-3.44646653795720
H	-1.24536177557517	2.77735063723194	-5.87856753891007

Compound **12** radical

C	-1.24536150168872	-0.55053553401120	-5.30906334259779
C	-1.24536192152735	-0.57669125619601	-3.88477277004609
C	-1.24536169489045	-1.76661919368432	-3.15565622987674
C	-1.24536204980076	0.69032379059810	-3.18020040244718
C	-1.24536163366516	0.60798939471113	-1.80801703885112
C	-1.24536208612337	-0.52781317855319	-1.03366801705767
C	-1.24536153115807	-1.78237861203785	-1.76019122825030
C	-1.24536256350809	0.63165682927813	-5.98975265051760
C	-1.24536129627163	1.90288663844811	-3.92902518500854
C	-1.24536206784410	-0.54896964570079	0.39134058855897
C	-1.24536168609394	-2.99177676026651	-1.00747140097447
C	-1.24536109087278	-2.96997107000348	0.35654059542732
C	-1.24536183233422	-1.73595721471680	1.06306830182560
H	-1.24536291763345	0.39301843964690	0.92440304193471

H	-1.24536197711762	-3.93205078301456	-1.54603816814746
H	-1.24536147908533	-3.89837073465402	0.91361563264229
H	-1.24536236929688	-1.74564447917541	2.14567086783194
C	-1.24536204709356	1.87096145129045	-5.29255747003471
H	-1.24536113993865	-1.49495204622225	-5.84040941074008
H	-1.24536221420136	-2.70898745375806	-3.69059643106722
H	-1.24536305193690	0.63380909814054	-7.07243504128033
H	-1.24536045825925	2.84337841180589	-3.39334756805860
H	-1.24536166365834	2.79544439207522	-5.85603394926492

Compound **13**

H	-0.21568911362718	-0.24080524410772	-3.41819389140952
C	-0.21568897983227	0.25563853409553	-2.45559226691912
C	-0.21568916218220	1.62152520720675	-2.37252324102421
C	-0.21568875867457	2.25934671347749	-1.11494470020190
C	-0.21568886648093	1.51356065706877	0.03482531713082
C	-0.21568912750180	0.09228716934747	0.00277608432809
C	-0.21568897246119	-0.53677482933000	-1.28408700093530
C	-0.21568889729713	-1.95077930598757	-1.35785323209096
C	-0.21568897439983	-2.71807758050216	-0.22334300540386
C	-0.21568894769694	-2.09945121103318	1.04357958986540
C	-0.21568927343202	-0.73298057299843	1.15272501902740
H	-0.21568902649430	2.22800169118040	-3.26923697605201
H	-0.21568875470113	3.33930619579944	-1.06658063489124
I	-0.21568871678503	2.56151435335307	1.87514167111993
H	-0.21568935609118	-0.27136128887560	2.13013701223421
H	-0.21568878789606	-2.70952436940983	1.93803091245106
H	-0.21568873836649	-3.79834732079373	-0.29455676168954
H	-0.21568882607974	-2.41391819049071	-2.33727904653924

Compound **13** radical

H	-0.21568960864941	-0.22244962577791	-3.43123726966533
C	-0.21568935611188	0.26504154151995	-2.46387581732554

C	-0.21568893710425	1.63351776050564	-2.38220109866189
C	-0.21568821248094	2.27976317089226	-1.11417322351747
C	-0.21568852434085	1.48024396324525	-0.02567920012403
C	-0.21568893581768	0.08457796128026	-0.00938617619370
C	-0.21568907986926	-0.54538544969588	-1.30074378688247
C	-0.21568882582602	-1.95939478381603	-1.35768711405334
C	-0.21568856428023	-2.70938130086823	-0.20973030755321
C	-0.21568924404318	-2.08395592561091	1.05624130517781
C	-0.21568933840382	-0.71674999522940	1.15731748097018
H	-0.21568943691492	2.23787515813410	-3.28115034579548
H	-0.21568828492067	3.36120297456813	-1.05058577125470
H	-0.21568985922575	-0.23161207506439	2.12493419883294
H	-0.21568919986278	-2.69238662384227	1.95176192339977
H	-0.21568775313972	-3.79043472342940	-0.26958073624680
H	-0.21568915900865	-2.43925560081118	-2.32929614510672

12. NMR spectra

2-bromo-1,3-bis(*N*-*tert*-butylmethanimino)benzene **S2**

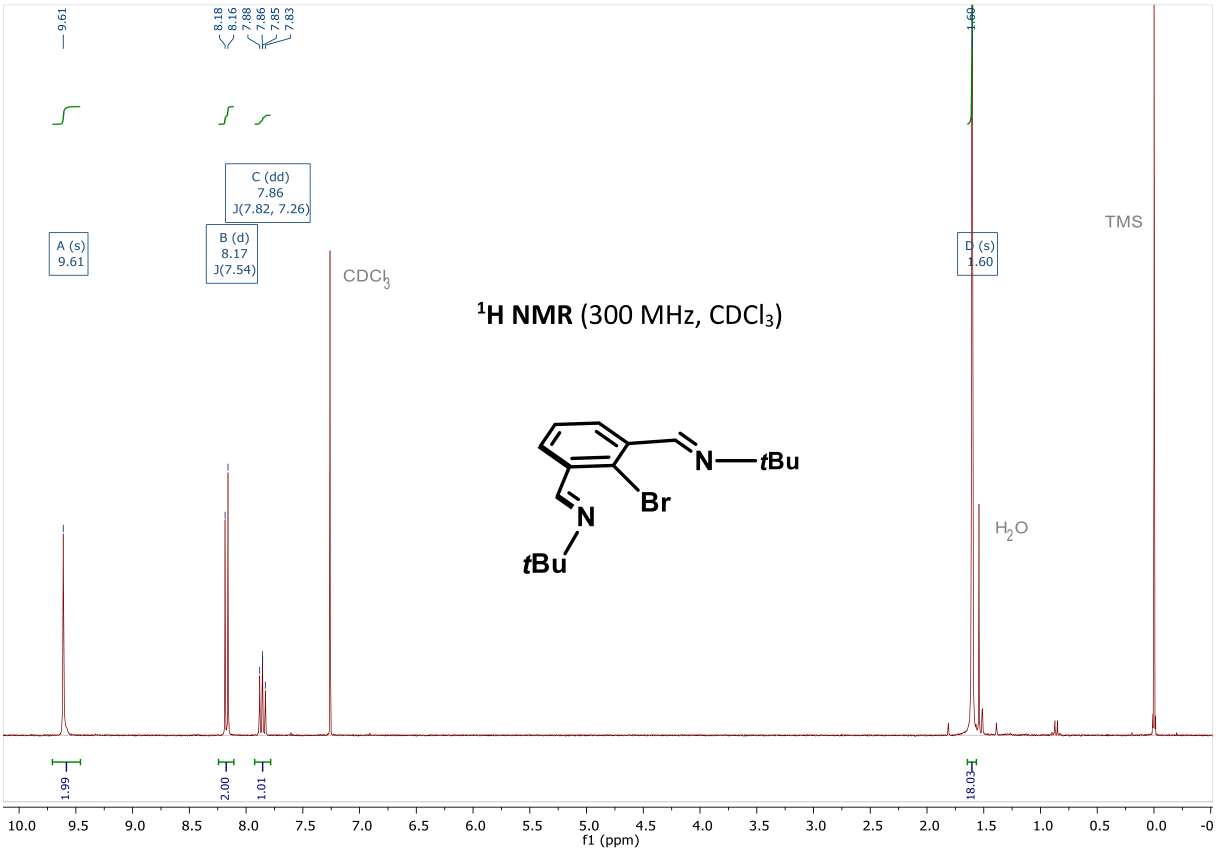

Dichlorobismuthine **1a-Cl$_2$**

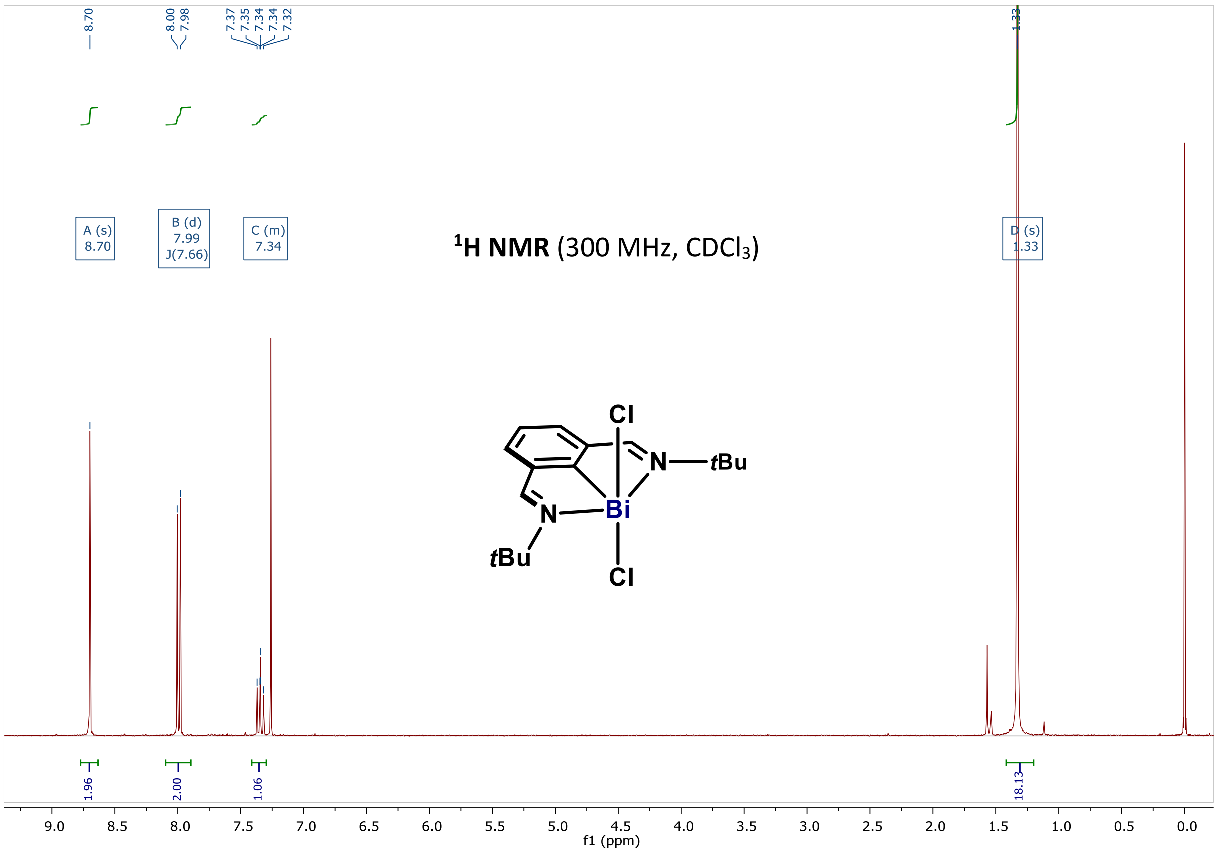

Bismuthinidene **1a**

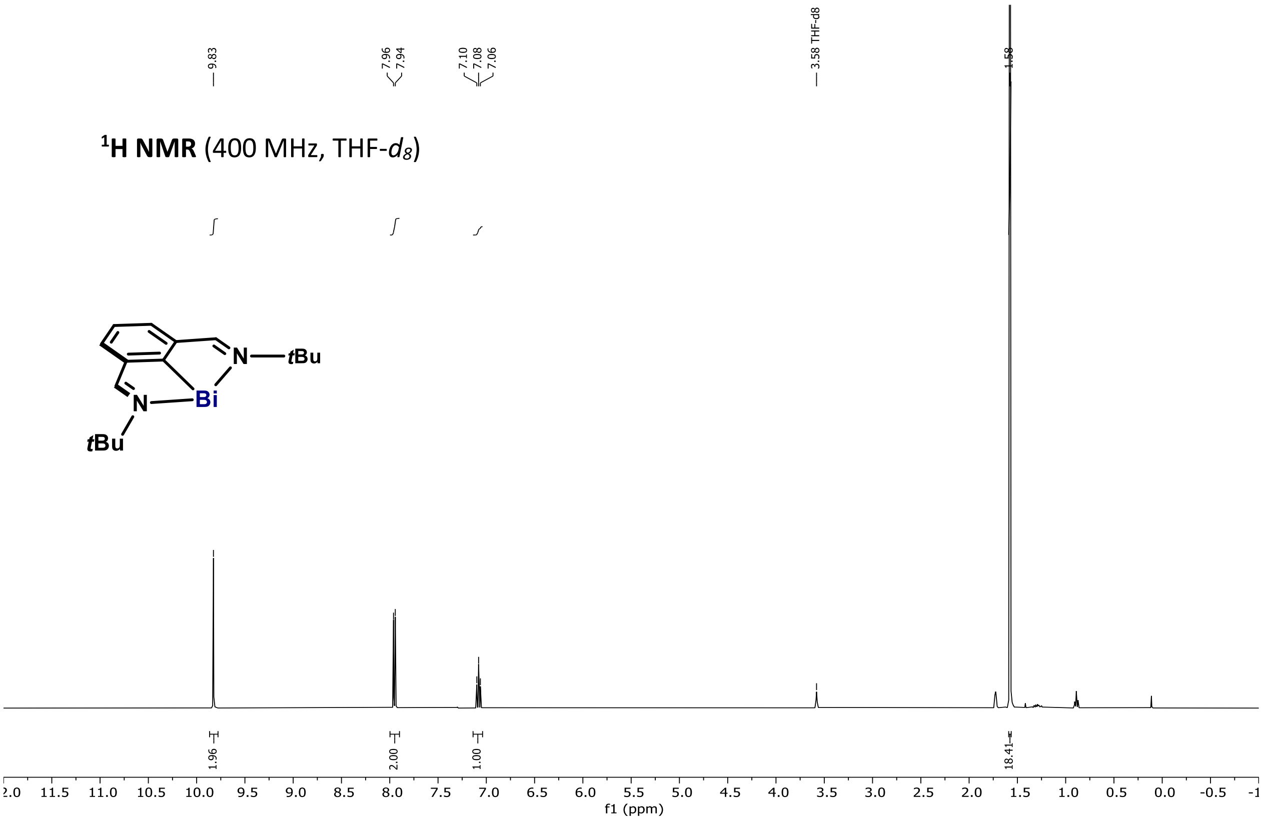

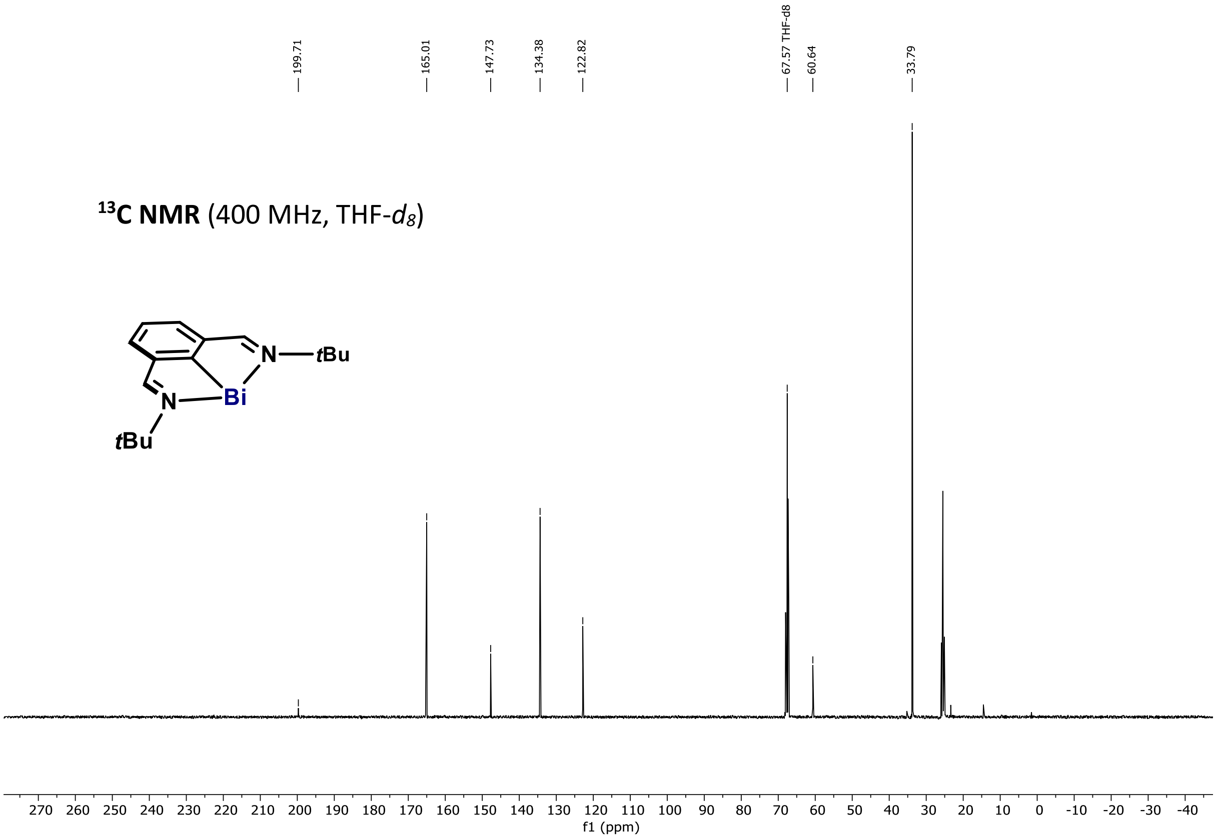

199.71
165.01
147.73
134.38
122.82
67.57 THF-d8
60.64
33.79
13C NMR (400 MHz, THF-d8)
N
tBu
Bi
N
tBu
270 260 250 240 230 220 210 200 190 180 170 160 150 140 130 120 110 100 90 80 70 60 50 40 30 20 10 0 -10 -20 -30 -40
f1 (ppm)

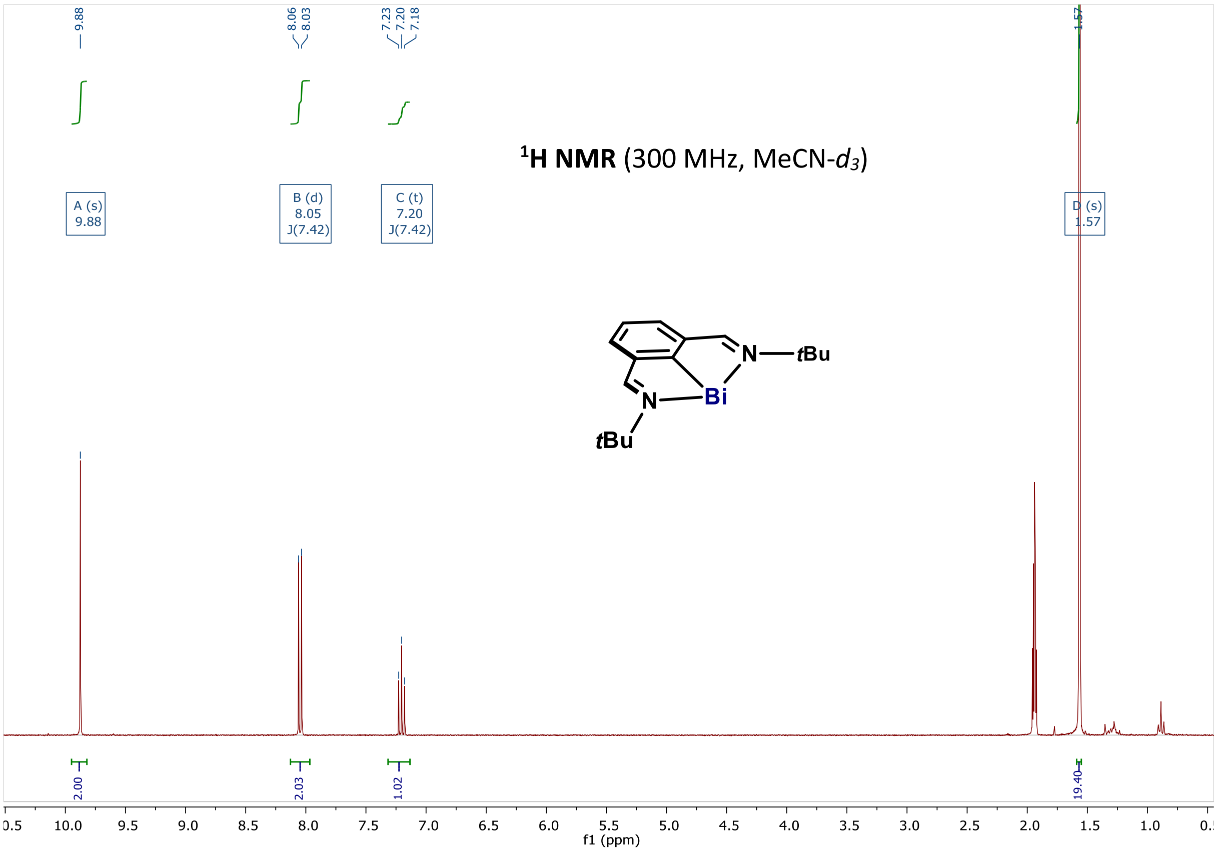

4-iodo-2,6-dimethyl aniline (**S3**)

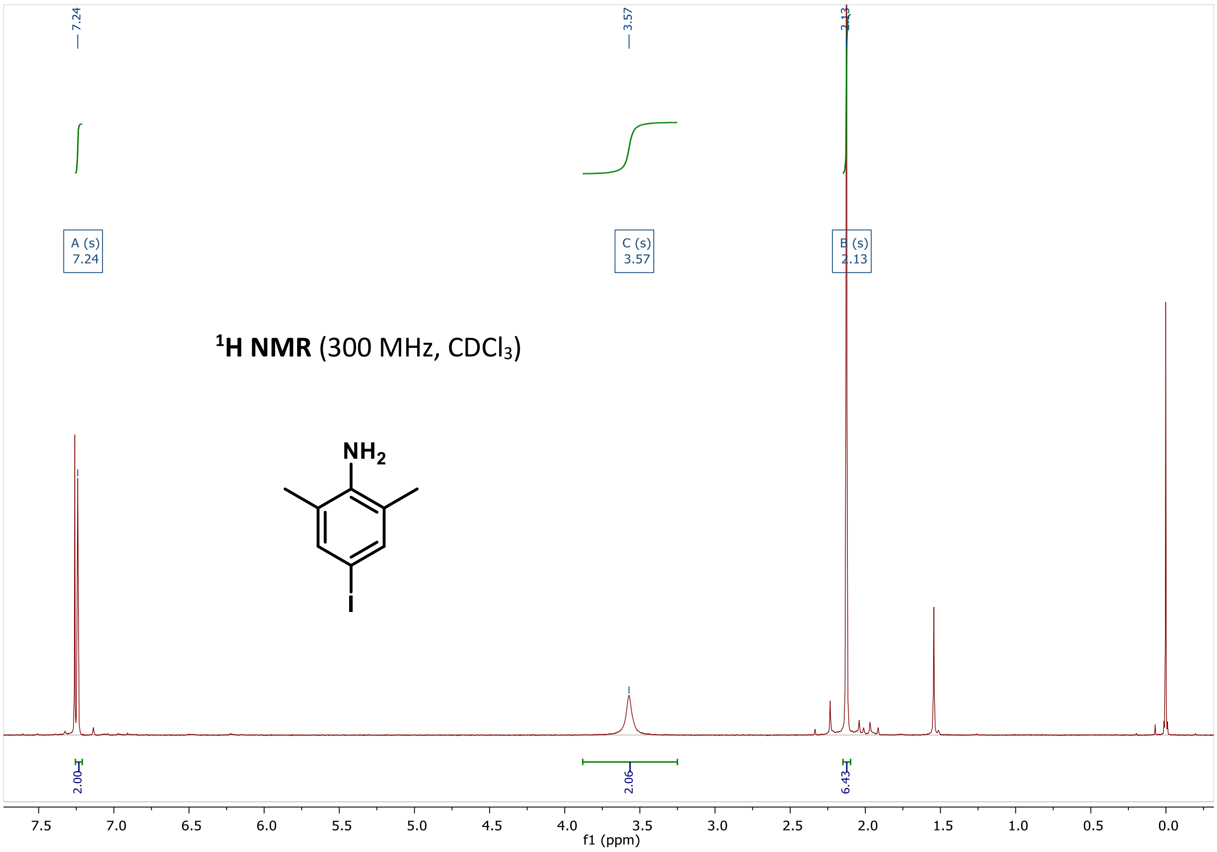

2,5-diiodo-1,3-dimethylbenzene (**6**)

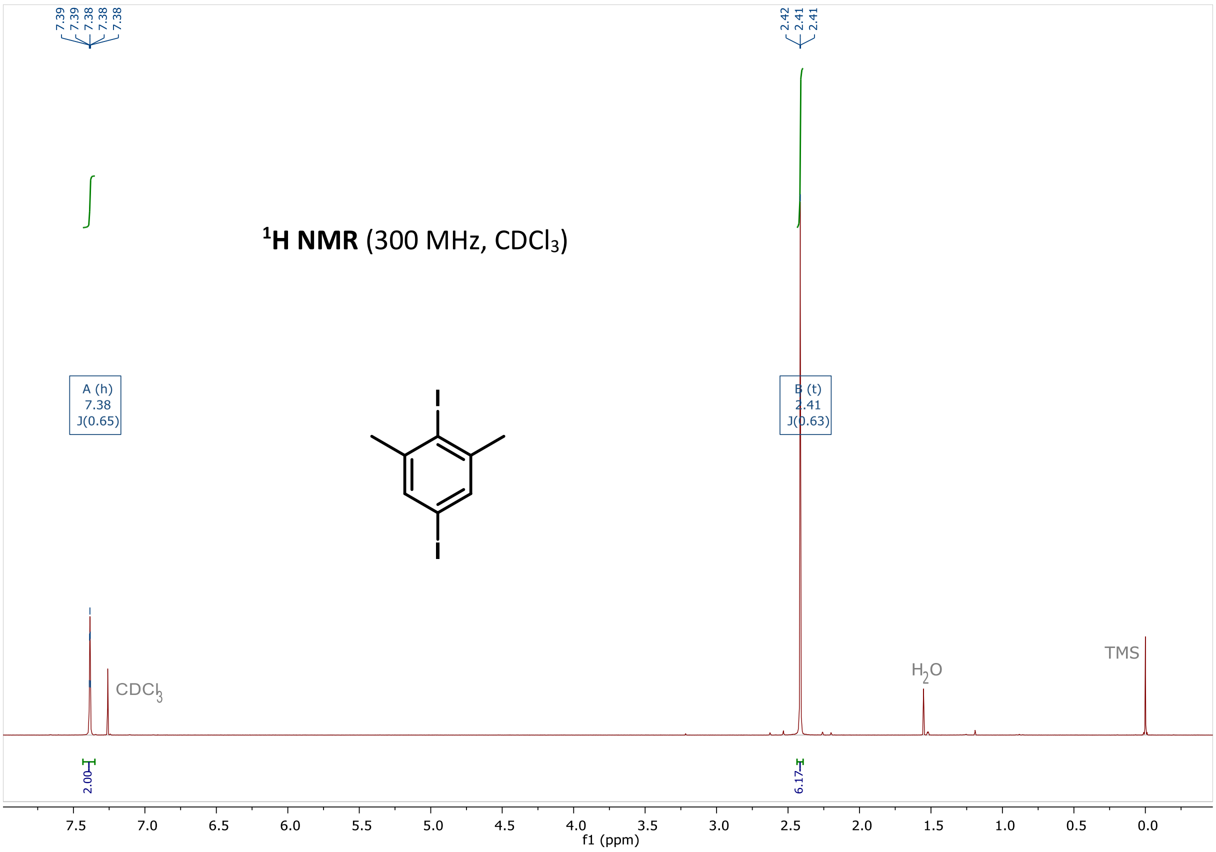

4-iodo-2,6-diisopropylanilinium chloride (**S4**)

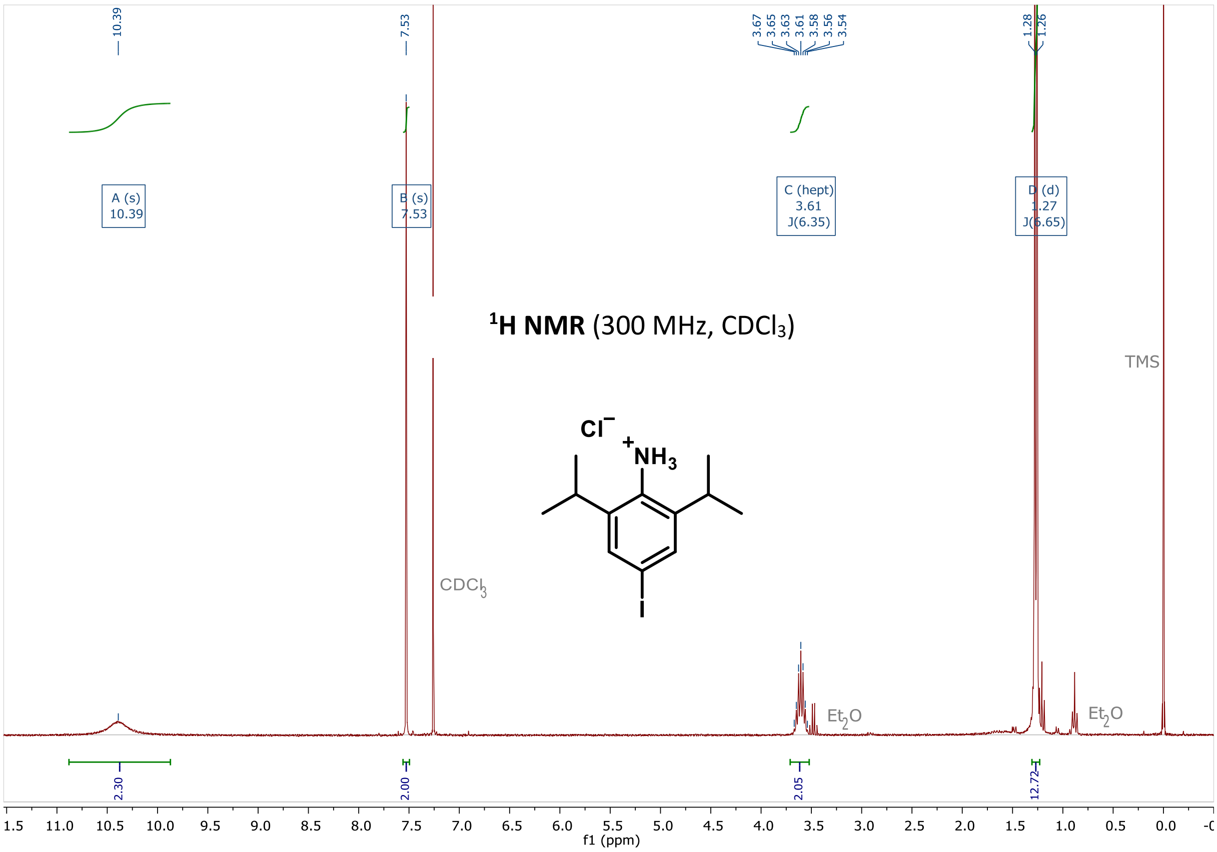

2,5-diiodo-1,3-diisopropylbenzene (**8**)

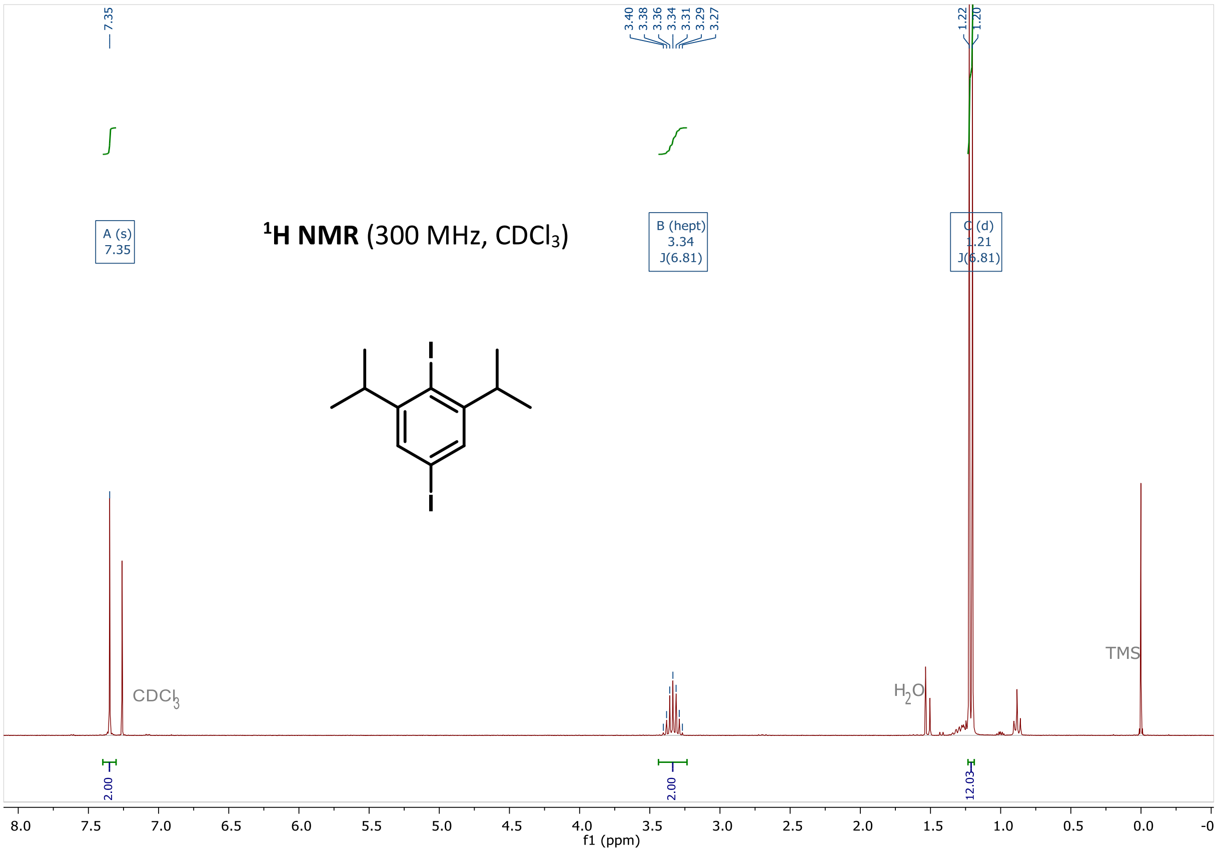

2 -iodo-1,3-diisopropylbenzene (**10**)

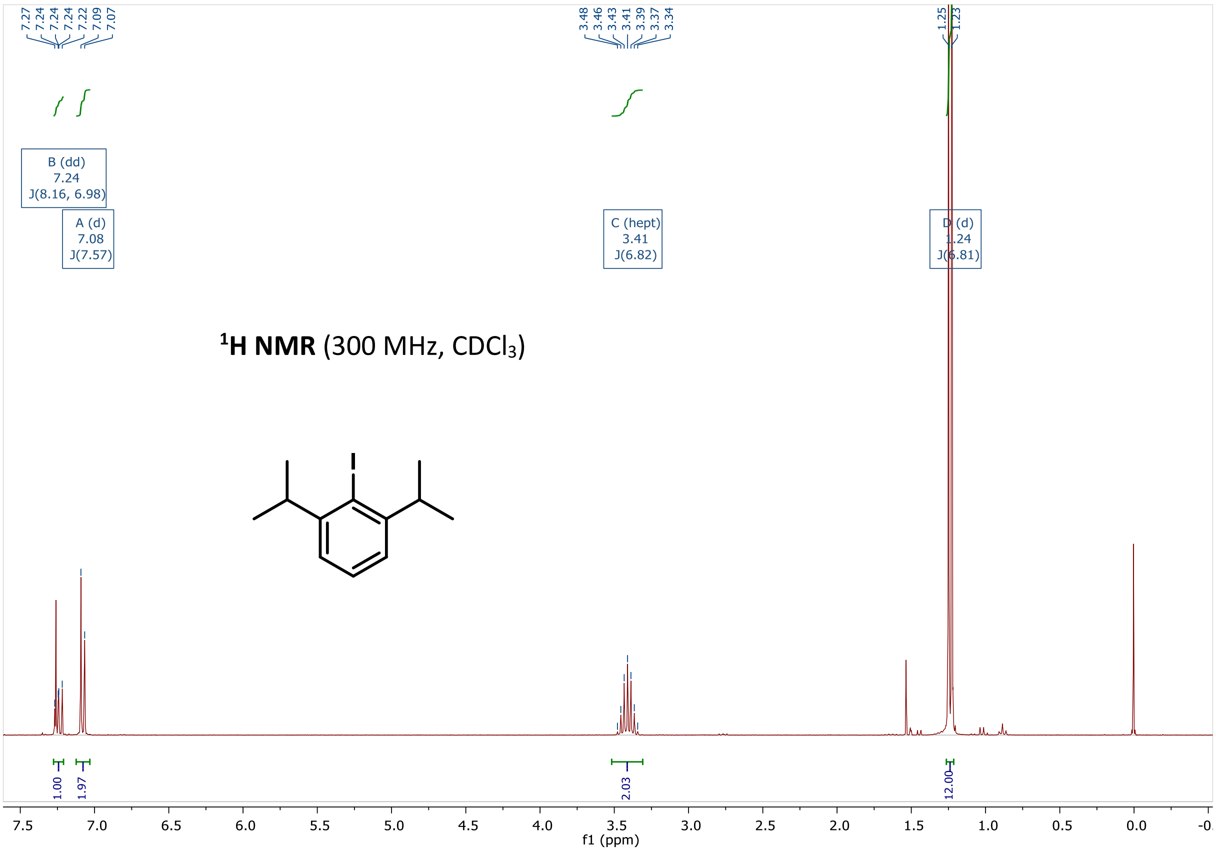

5-fluoro-2-iodo-1,3-dimethylbenzene (**S5**)

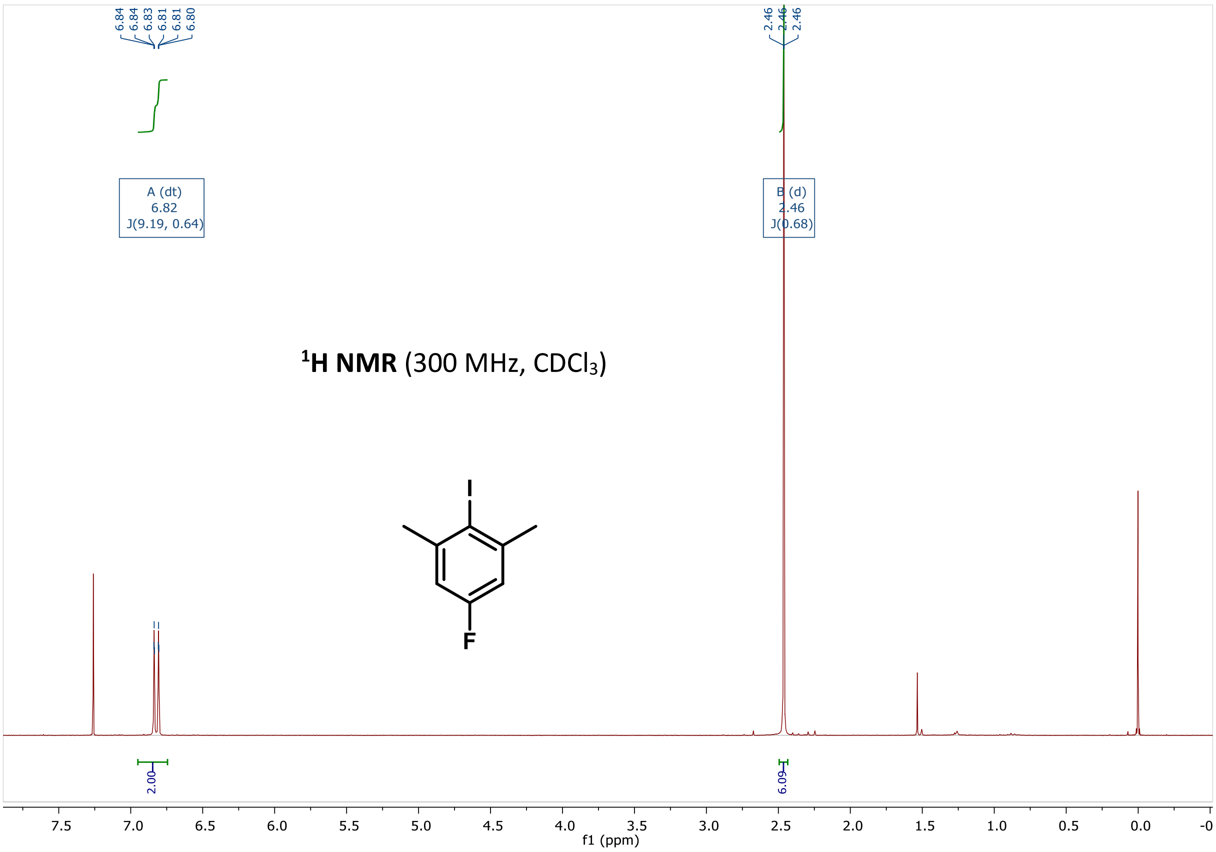

[(2,6-(*t*BuNCH)$_2$C$_6$H$_3$)Bi(4-cyanophenyl)(iodide)] (3a)

1H{off,off},1D, 600.22 MHz,CD3CN,298.0K, pulse sequence: zg30

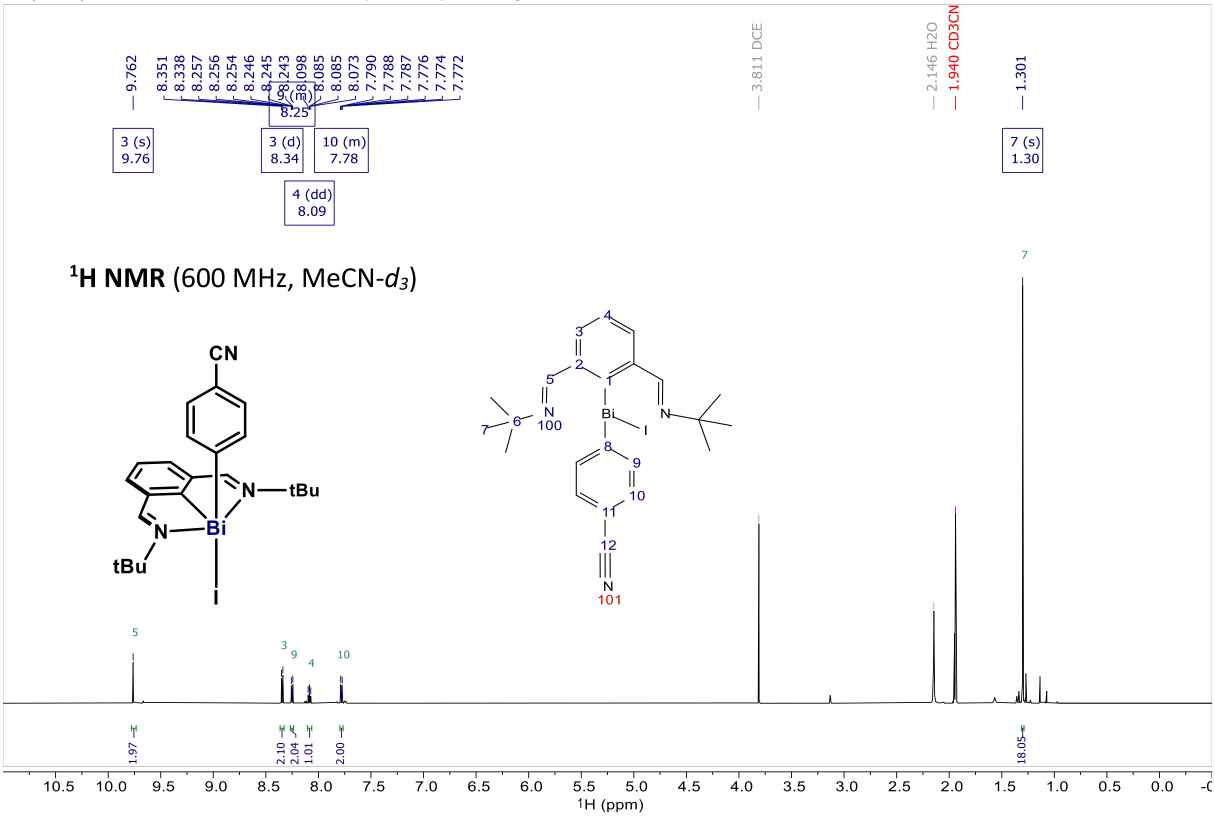

13C{1H,off},1D, 150.94 MHz,CD3CN,298.0K, pulse sequence: zgdc30

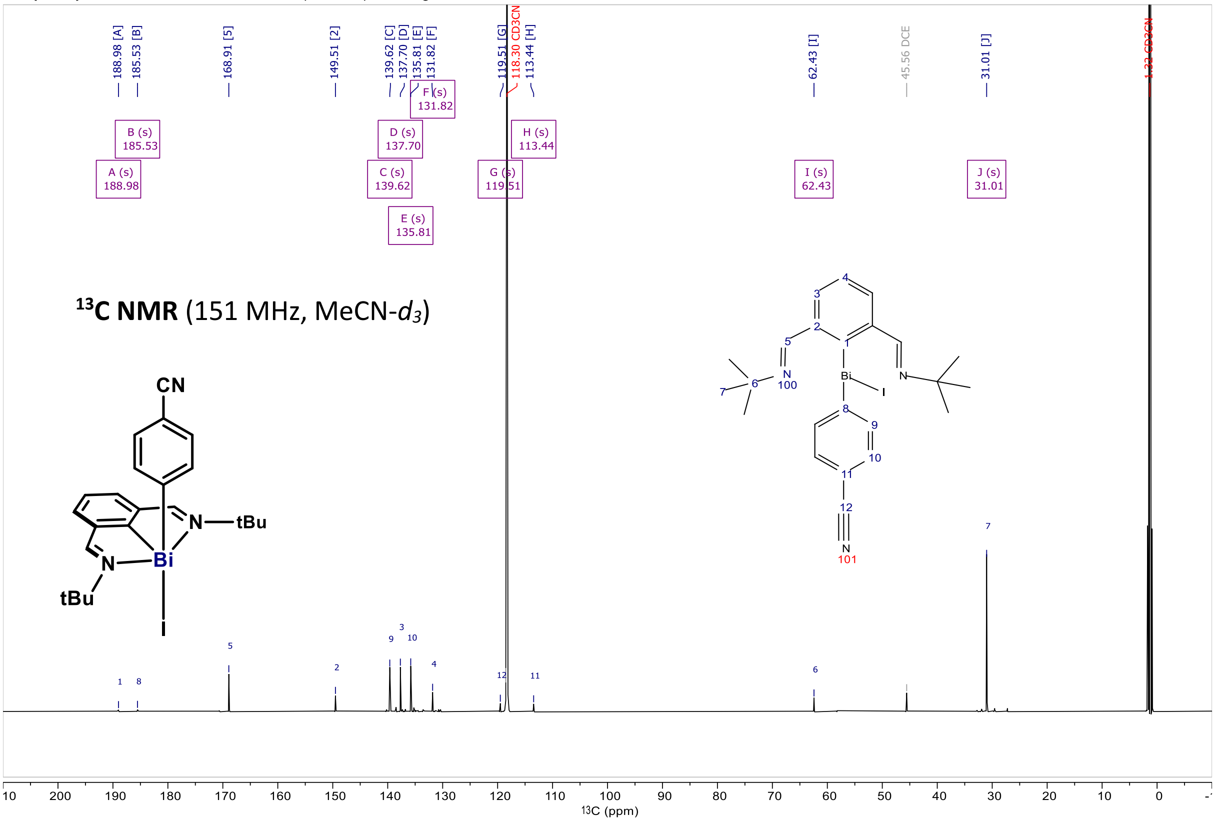

^1H-^{13}C-edited HSQC

1H{13C,off},HSQC-EDITED, 600.22 MHz,CD3CN,298.0K, pulse sequence: hsqcedetgpsisp2.3

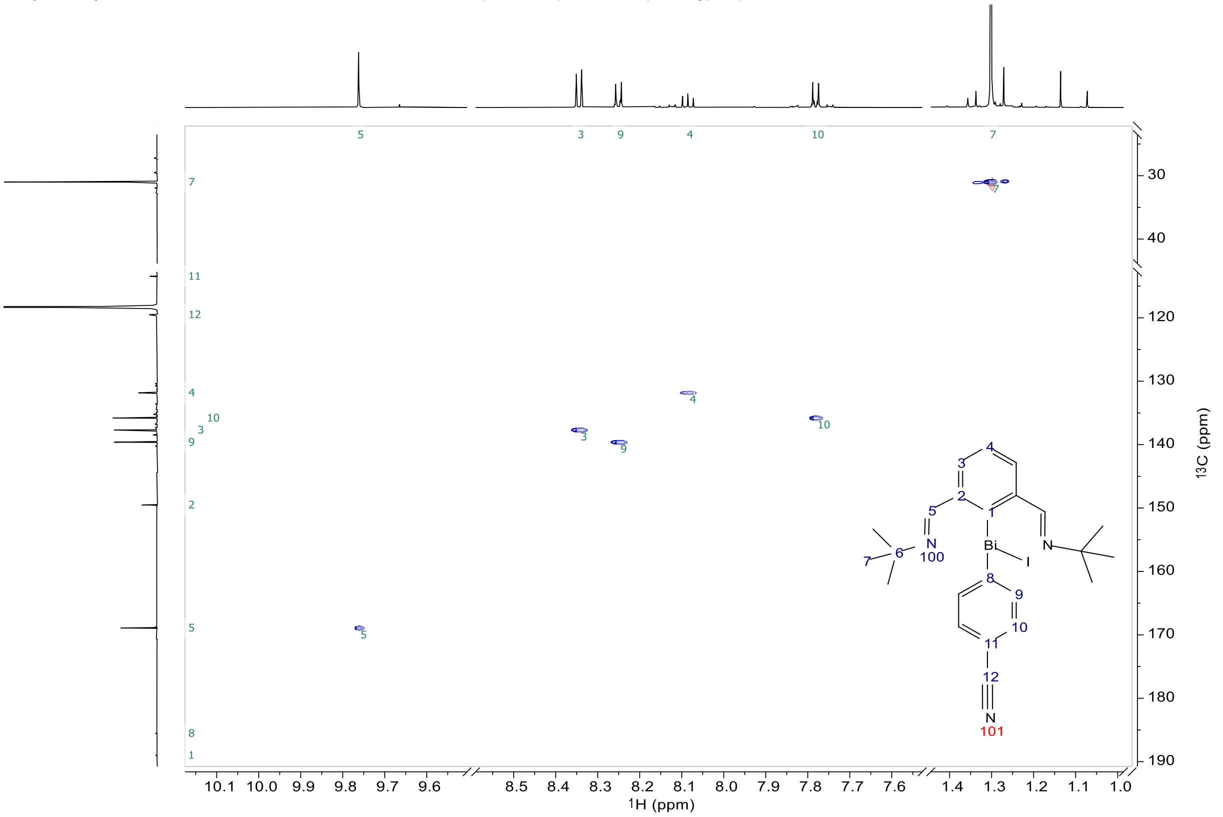

^1H-^{13}C HMBC

1H{13C,off},HMBC, 600.22 MHz,CD3CN,298.0K, pulse sequence: hmbcetgpl3nd

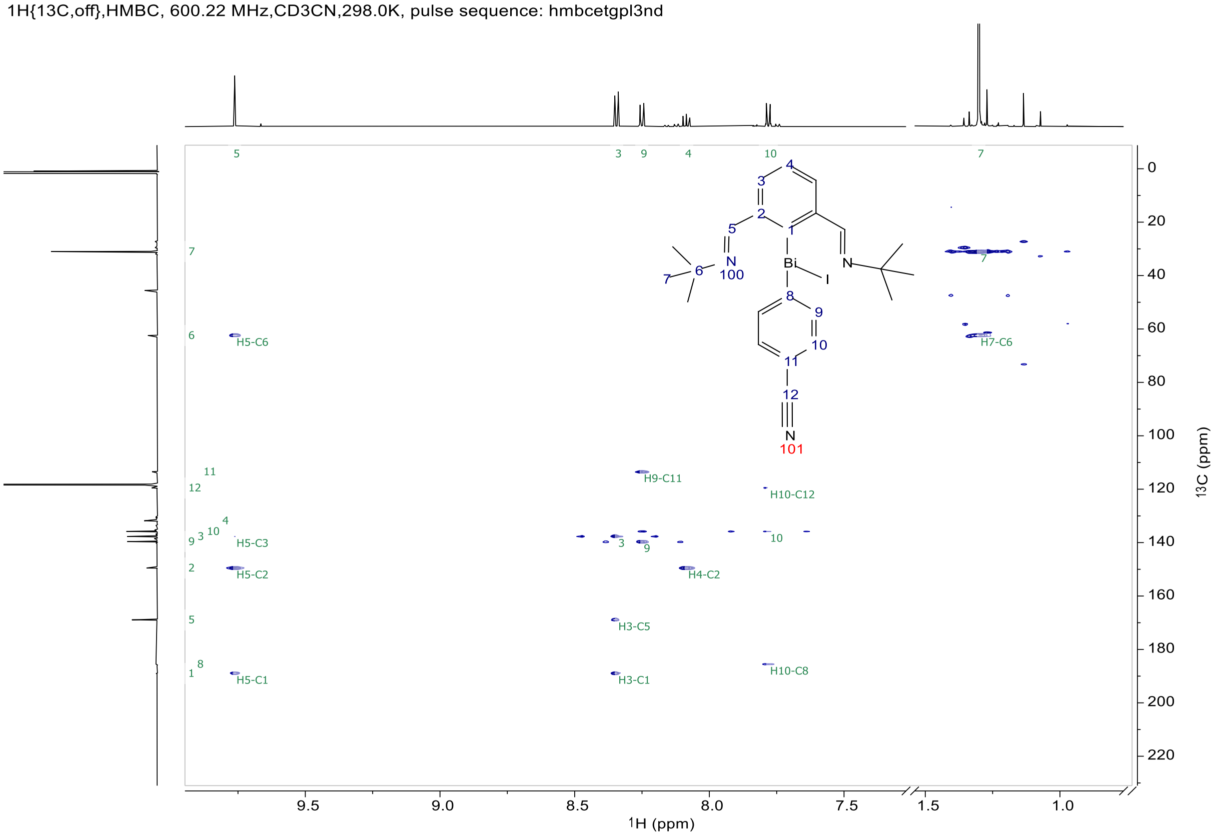

^{1}H-^{1}H COSY

1H{off,off},COSY, 600.22 MHz,CD3CN,298.0K, pulse sequence: cosygpppqf

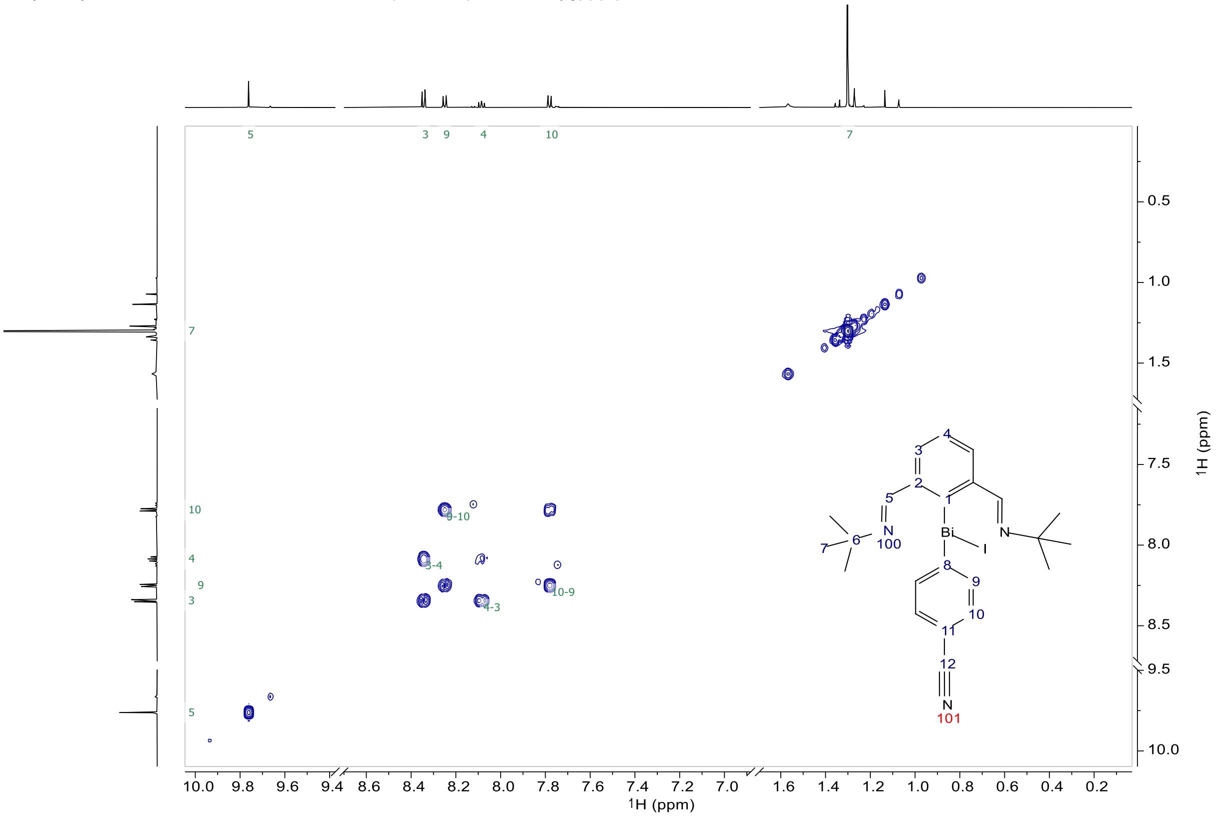

^{1}H-^{1}H NOESY

1H{off,off},NOESY, 600.22 MHz,CD3CN,298.0K, pulse sequence: noesygpph

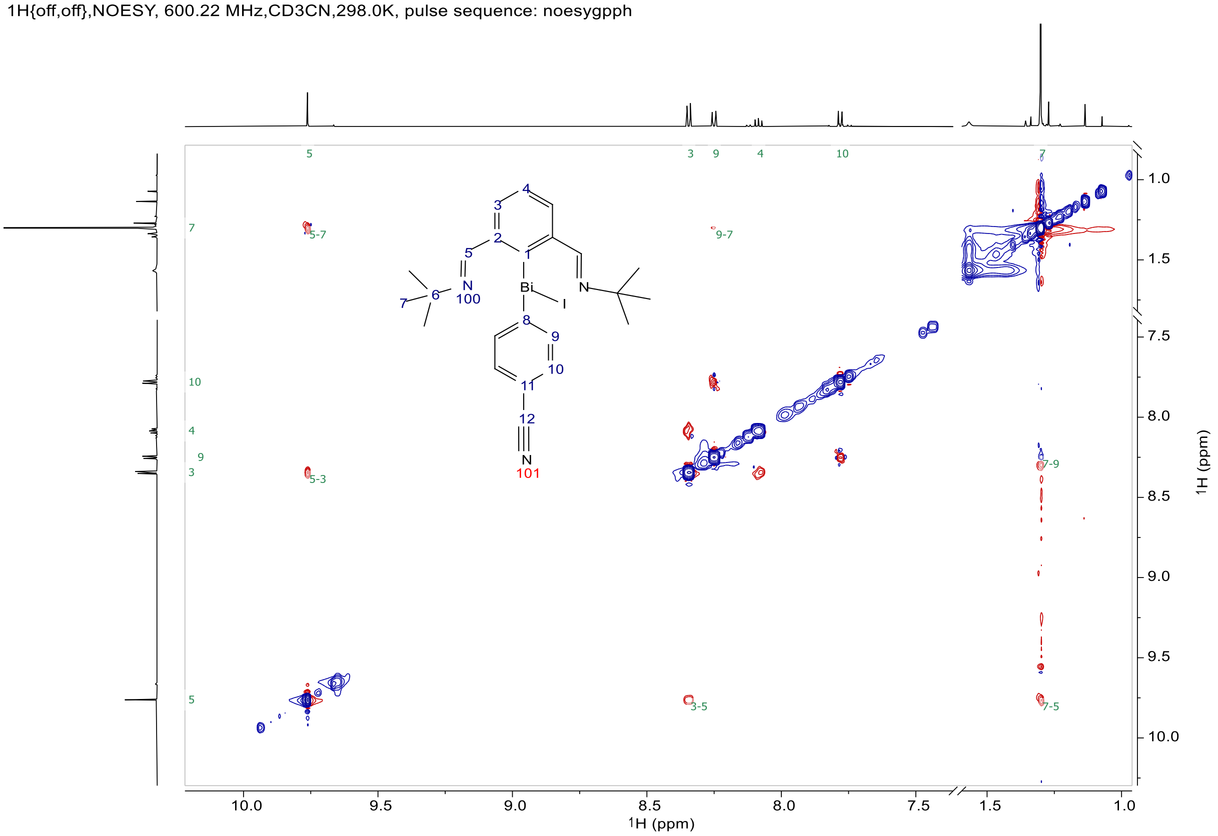

^{1}H-^{15}N HMBC

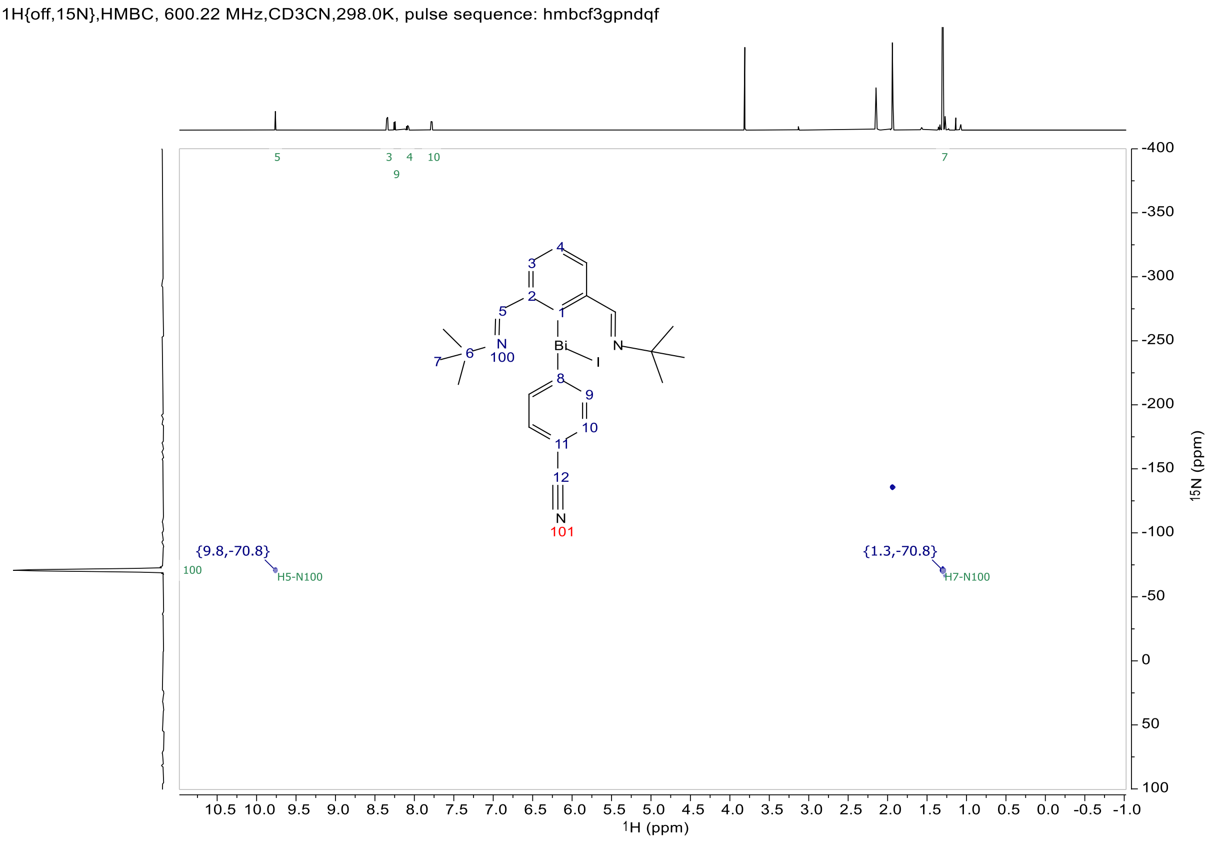

1H{off,15N},HMBC, 600.22 MHz,CD3CN,298.0K, pulse sequence: hmbcf3gpndqf
5
3
4
10
9
7
{9.8,-70.8}
100
H5-N100
{1.3,-70.8}
H7-N100
Bi
I
N
100
101
1H (ppm)
15N (ppm)

[(2,6-(*t*BuNCH)$_2$C$_6$H$_3$)Bi(4-fluorophenethyl)(phthalimide)] (18a)

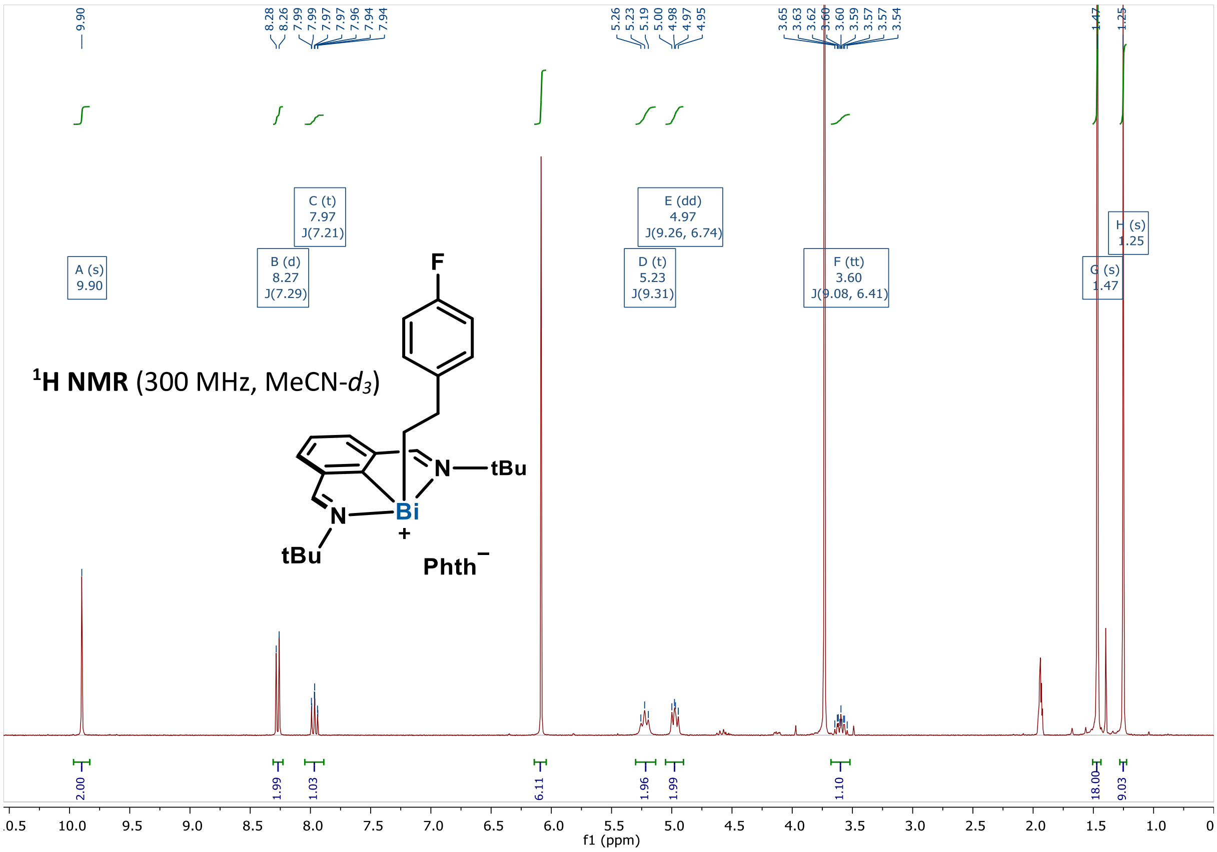

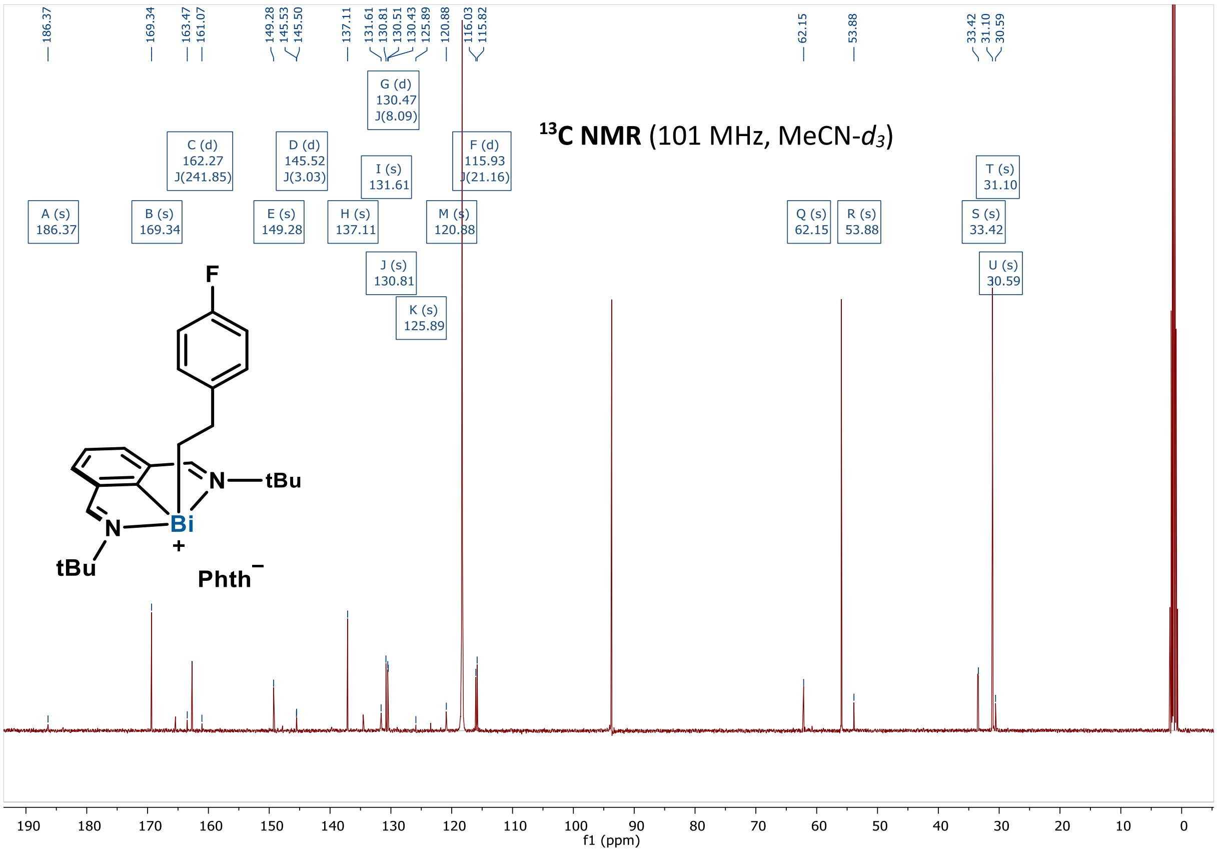

[(2,6-(*t*BuNCH)$_2$C$_6$H$_3$)Bi(*N*-Boc-3-azetidinyl)(iodide)] (18b)

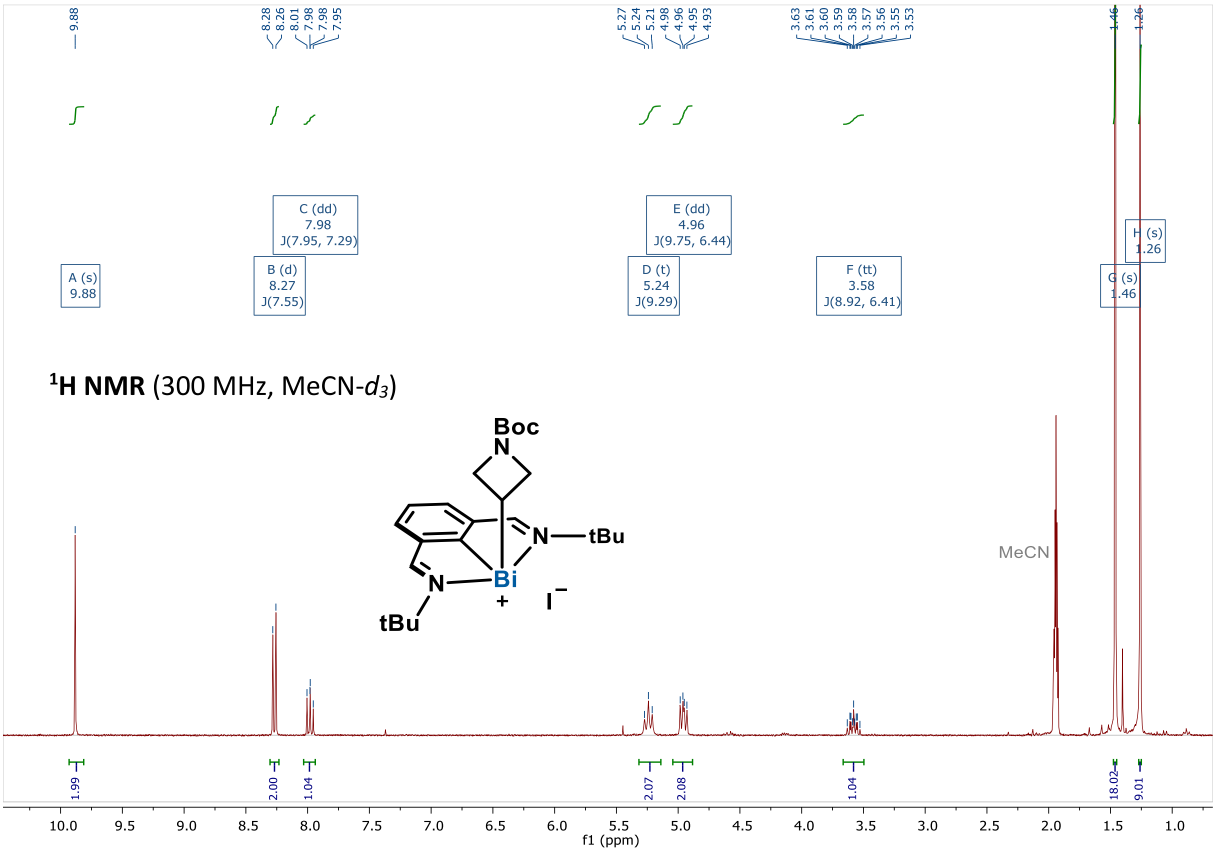

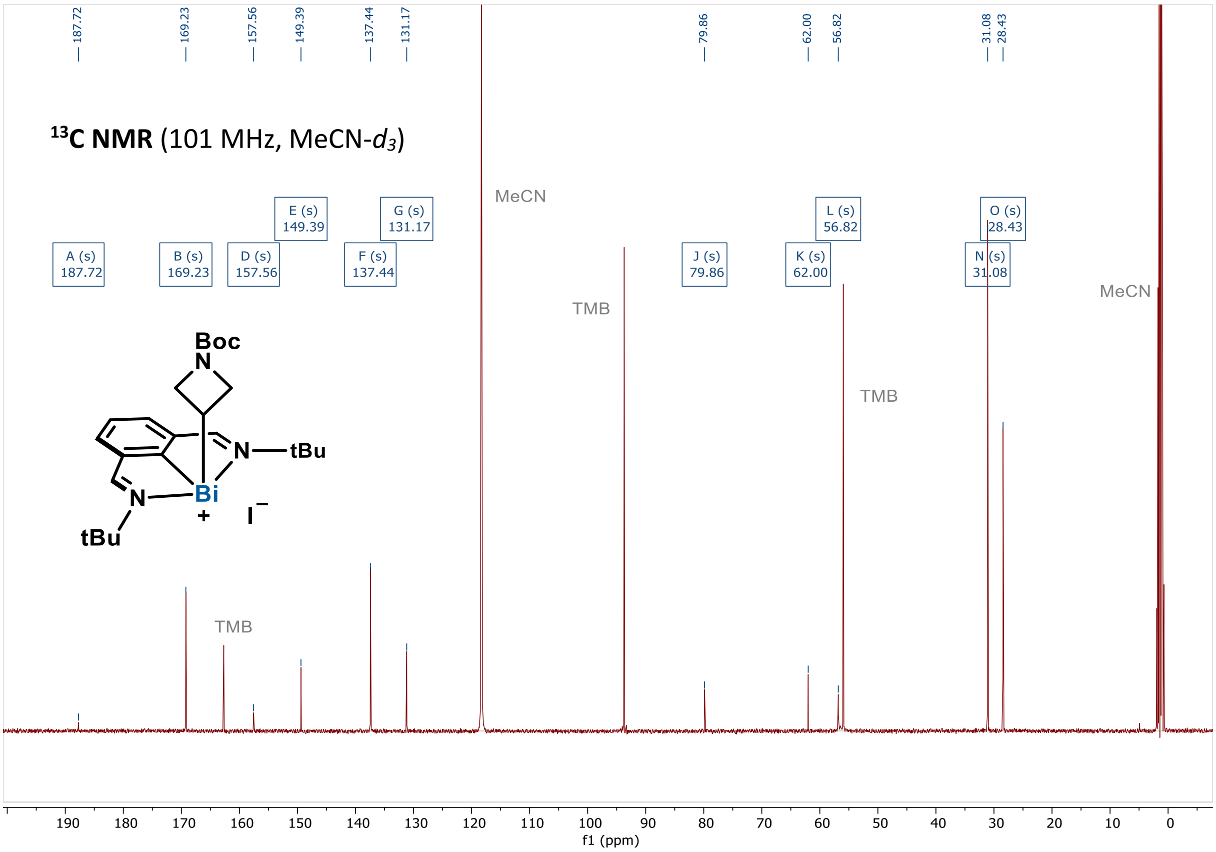

[(2,6-(*t*BuNCH)$_2$C$_6$H$_3$)Bi(*N*-Boc-3-azetidinyl)(bromide)] (18c)

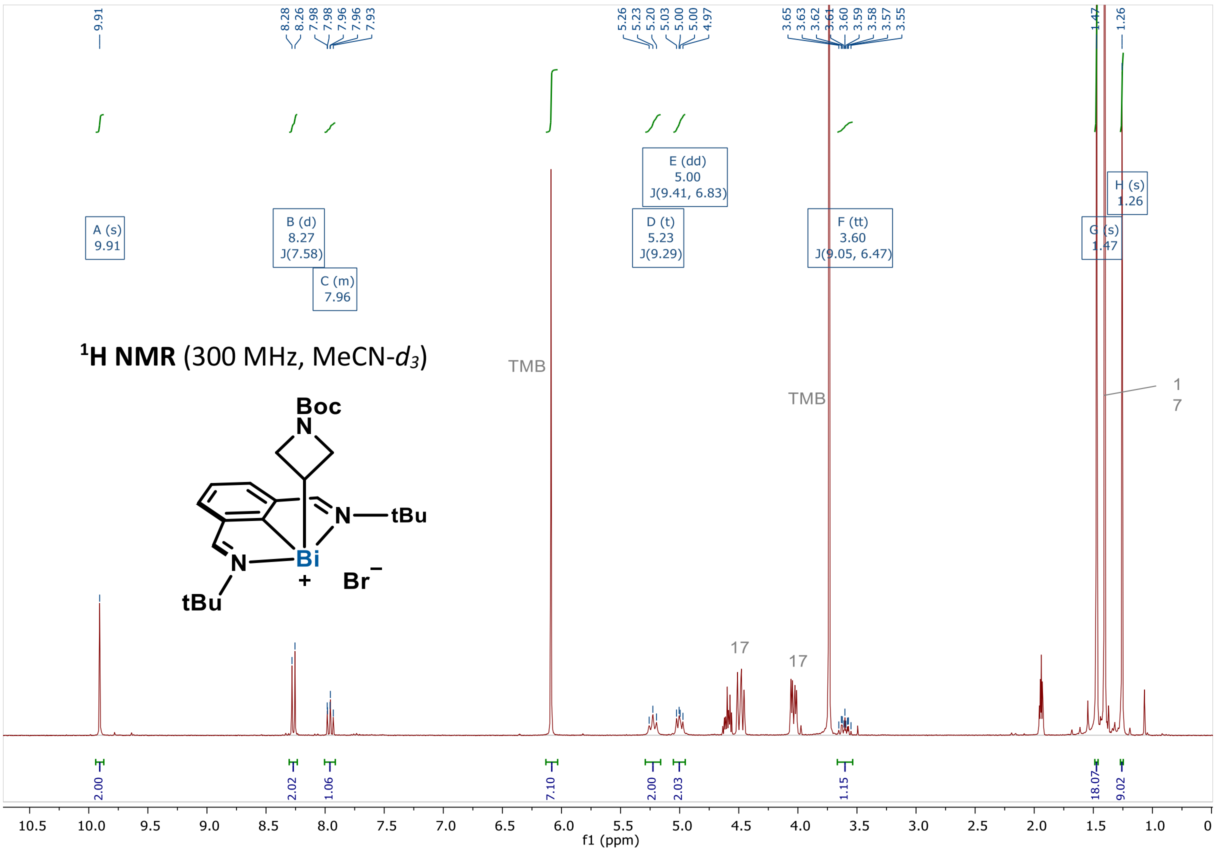

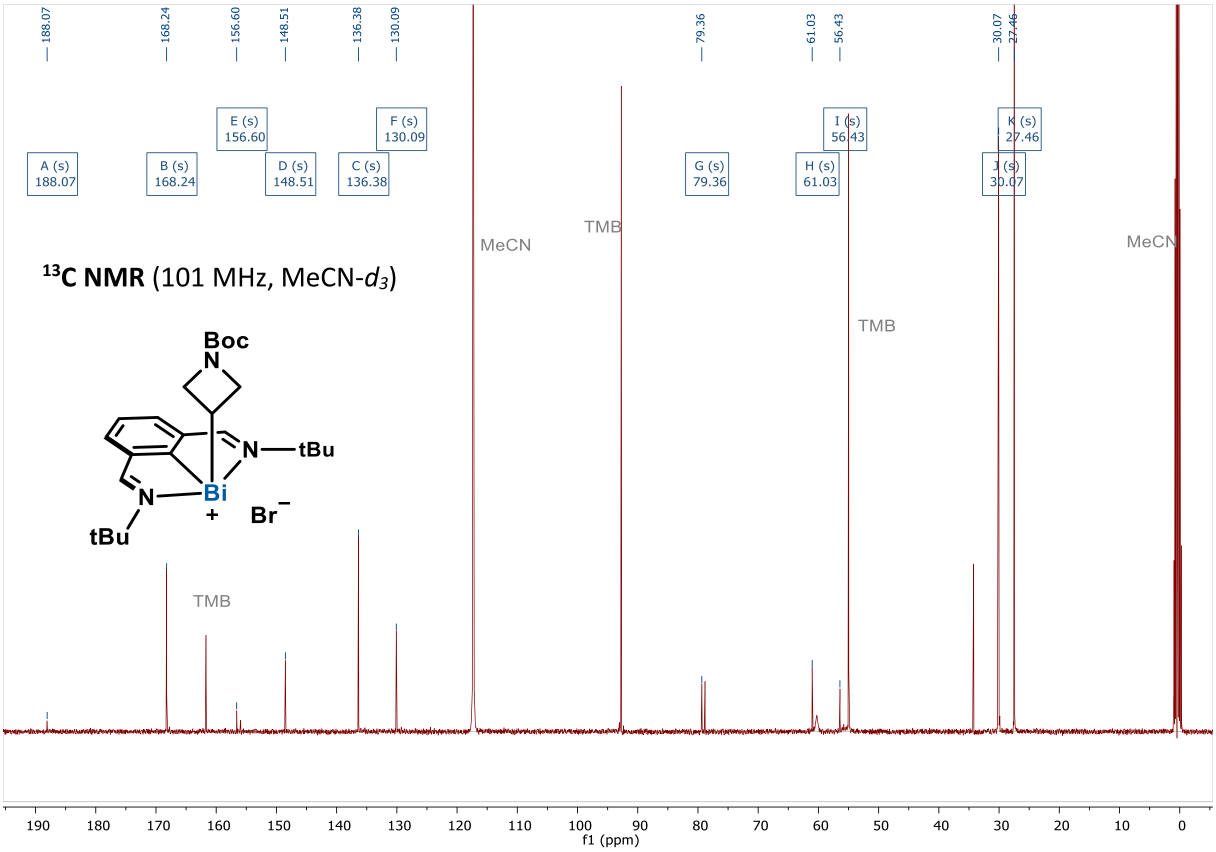

12. References

1. Vránová, I.; Alonso, M.; Lo, R.; Sedlák, R.; Jambor, R.; Růžička, A.; Dr Proft, F.; Hobza, P.; Dostál, L. From Dibismuthenes to Three- and Two-Coordinated Bismuthinidenes by Fine Ligand Tuning: Evidence for Aromatic BiC3N Rings through a Combined Experimental and Theoretical Study. *Chem. Eur. J.* **2015**, *21*, 16917–16928.
2. Wang, F.; Planas, O.; Cornella, J. Bi(I)-Catalyzed Transfer-Hydrogenation with Ammonia-Borane. *J. Am. Chem. Soc.* **2019**, *141*, 4235–4240.
3. Mato, M.; Bruzzese, P. C.; Takahashi, F.; Leutzsch, M.; Reijerse, E. J.; Schnegg, A.; Cornella, J. *J. Am. Chem. Soc.* **2023**, *145*, 18742–18747.
4. van Stokkum, I. H. M.; Larsen, D. S.; van Grondelle, R. Global and target analysis of time-resolved spectra. *BBA – Bioenergetics* **2004**, *1657*, 82–104.
5. Snellenburg, J. J.; Laptenok, S.; Seger, R.; Mullen, K. M.; van Stokkum, I. H. M. Glotaran: A Java-Based Graphical User Interface for the R Package TIMP. *J. Stat. Soft.* **2012**, *49*, 1–22.
6. Mullen, K. M.; van Stokkum, I. H. M. TIMP: An R Package for Modeling Multi-way Spectroscopic Measurements. *J. Stat. Soft.* **2007**, *18*, 1–46.
7. Neese, F. Software update: The ORCA program system—Version 5.0. *WIREs Comput. Mol. Sci.* **2022**, *12*, e1606.
8. Neese, F.; Wennmohs, F.; Becker, U.; Riplinger, C. The ORCA quantum chemistry program package. *J. Chem. Phys.* **2020**, *152*, 224108.
9. Kutzelnigg, W.; Liu, W. Quasirelativistic theory equivalent to fully relativistic theory. *J. Chem. Phys.* **2005**, *123*, 241102.
10. Becke, A. D. Density-functional thermochemistry. III. The role of exact Exchange. *J. Chem. Phys.* **1993**, *98*, 5648–5652.
11. Lee, C.; Yang, W.; Parr, R. G. Development of the Colle-Salvetti correlation-energy formula into a functional of the electron density. *Phys. Rev. B* **1988**, *37*, 785.
12. Pollak, P.; Weigend, F. Segmented Contracted Error-Consistent Basis Sets of Double- and Triple-ζ Valence Quality for One- and Two-Component Relativistic All-Electron Calculations. *J. Chem. Theory Comput.* **2017**, *13*, 3696–3705.
13. Neese, F. An improvement of the resolution of the identity approximation for the formation of the Coulomb matrix. *J. Comput. Chem.* **2003**, *24*, 1740–1747.
14. Neese, F.; Wennmohs, F.; Hansen, A.; Becker, U. Efficient, approximate and parallel Hartree–Fock and hybrid DFT calculations. A 'chain-of-spheres' algorithm for the Hartree–Fock exchange. *Chem. Phys.* **2009**, *356*, 98–109.
15. Grimme, S.; Antony, J.; Ehlrich, S.; Krieg, H. A consistent and accurate ab initio parametrization of density functional dispersion correction (DFT-D) for the 94 elements H-Pu. *J. Chem. Phys.* **2010**, *132*, 154104.
16. Marenich, A. V.; Cramer, C. J.; Truhlar, D. G. Universal Solvation Model Based on Solute Electron Density and on a Continuum Model of the Solvent Defined by the Bulk Dielectric Constant and Atomic Surface Tensions. *J. Phys. Chem. B* **2009**, *113*, 6378–6396.
17. Garcia-Ratés, M.; Neese, F. Effect of the Solute Cavity on the Solvation Energy and its Derivatives within the Framework of the Gaussian Charge Scheme. *J. Comput. Chem.* **2019**, *41*, 922–939.

18. Guo, Y.; Sivalingam, K.; Neese, F. Approximations of density matrices in N-electron valence state second-order perturbation theory (NEVPT2). I. Revisiting the NEVPT2 construction. *J. Chem. Phys.* **2021**, *154*, 214111.

19. Schapiro, I.; Sivalingam, K.; Neese, F. Assessment of n-Electron Valence State Perturbation Theory for Vertical Excitation Energies. *J. Chem. Theory Comput.* **2013**, *9*, 3567–3580.

20. Heß, B. A.; Marian, C. M.; Wahlgren, U.; Gropen, O. A mean-field spin-orbit method applicable to correlated wavefunctions. *Chem. Phys. Lett.* **1996**, *251*, 365–371.

21. Neese, F. Efficient and accurate approximations to the molecular spin-orbit coupling operator and their use in molecular *g*-tensor calculations. *J. Chem. Phys.* **2005**, *122*, 034107.

22. Neese, F.; Hansen, A.; Liakos, D. G. Efficient and accurate approximations to the local coupled cluster singles doubles method using a truncated pair natural orbital basis. *J. Chem. Phys.* **2009**, *131*, 064103.

23. Riplinger, C.; Neese, F. An efficient and near linear scaling pair natural orbital based local coupled cluster method. *J. Chem. Phys.* **2013**, *138*, 034106.

24. Riplinger, C.; Sandhoefer, B.; Hanse, A.; Neese, F. Natural triple excitations in local coupled cluster calculations with pair natural orbitals. *J. Chem. Phys.* **2013**, *139*, 134101.

25. Riplinger, C.; Pinski, P.; Becker, U.; Valeev, E. F.; Neese, F. Sparse maps—A systematic infrastructure for reduced-scaling electronic structure methods. II. Linear scaling domain based pair natural orbital coupled cluster theory. *J. Chem. Phys.* **2016**, *144*, 024109.

26. Saitow, M. Becker, U.; Riplinger, C.; Valeev, E. F.; Neese, F. A new near-linear scaling, efficient and accurate, open-shell domain-based local pair natural orbital coupled cluster singles and doubles theory. *J. Chem. Phys.* **2017**, *146*, 164105.

27. Stoychev, G. L.; Auer, A. A.; Neese, F. Automatic Generation of Auxiliary Basis Sets. *J. Chem. Theory Comput.* **2017**, *13*, 554–562.

28. Pyykko, P. Relativistic effects in structural chemistry. *Chem. Rev.* **1988**, *88*, 563–594.

29. McCusker, J. K. Electronic structure in the transition metal block and its implications for light harvesting. *Science*, **2019**, *363*, 484–488.
